# Burden and trends of thyroid cancer among women of childbearing age, 1990–2021: A serial cross-sectional analysis

**DOI:** 10.1097/MD.0000000000045930

**Published:** 2026-05-12

**Authors:** Hongzhou Liu, Lang Xie, Yi Shen, Yangfan Du, Jia Liu, Linlin Wang, Xiaojing Li, Shuai Jin, Hongmei Liu, Song Dong

**Affiliations:** a Department of Endocrinology, Aerospace Center Hospital, Beijing, China; b Department of Endocrinology, First Hospital of Handan City, Handan, Hebei Province, China; c Department of Hospital Infection Management and Preventive Health Care, Zhejiang Provincial People's Hospital Bijie Hospital, Bijie, China; d School of Biology and Engineering (School of Health Medicine Modern Industry), Guizhou Medical University, Guiyang, China.

**Keywords:** AAPC, global burden of disease study, high body mass index, thyroid cancer, women of childbearing age

## Abstract

Thyroid cancer, particularly prevalent among women of childbearing age (WCBA), presents a growing global public health concern. This study examines the global, regional, and national burden of WCBA thyroid cancer from 1990 to 2021, with an emphasis on socioeconomic factors, including high body mass index. Data were drawn from the Global Burden of Diseases, Injuries, and Risk Factors Study 2021. We analyzed incidence, prevalence, disability-adjusted life years, and mortality rates for WCBA thyroid cancer, focusing on age-standardized rates. Temporal trends were quantified using the average annual percentage change. Inequalities were assessed using the concentration index; locally estimated scatterplot smoothing modeled age-standardized rate–sociodemographic index (SDI) relationships; decomposition identified contributions of population growth, aging, and epidemiologic change. From 1990 to 2021, the global incidence of WCBA thyroid cancer increased with an average annual percent change (AAPC) of 1.4% (95% confidence interval [CI]: 1.32–1.48), while prevalence rose at an AAPC of 1.44% (95% CI: 1.37–1.52). Conversely, the age-standardized mortality rate declined annually by 0.28% (95% CI: −0.4 to −0.16), and disability-adjusted life years showed no significant change (AAPC = −0.01, 95% CI: −0.18 to 0.17). The highest increase in disease burden was observed in low and low-middle SDI regions, with mortality rates linked to high body mass index rising by 2.19% annually. In contrast, high-SDI regions experienced a decrease in burden, with annual reductions in mortality rates ranging from 1.13% to 0.34%. Rising WCBA thyroid cancer incidence and prevalence, particularly in low SDI regions, highlight the urgent need for targeted prevention and health management strategies.

## 1. Introduction

Thyroid cancer, a common endocrine malignancy, is notably female-dominated, particularly prevalent among women of childbearing age (WCBA), and is the second most commonly diagnosed malignancy during pregnancy, following breast cancer.^[1,2]^ The Global Burden of Diseases (GBD), Injury, and Risk Factors Study 2019 estimated approximately 2,34,000 thyroid cancer cases worldwide, with an incidence rate of about 2.8 per 1,00,000.^[2]^ Similarly, the GBD 2017 study highlighted that women aged 15 to 49 years are at peak risk for thyroid cancer, with rising incidence rates.^[3,4]^ According to the United Nations’ 2030 global maternal mortality goals,^[5]^ women with thyroid cancer are at increased risk of adverse pregnancy outcomes due to pregnancy-related complications.^[6]^ Although some research has begun to focus on the WCBA group,^[7–9]^ comprehensive global analyses of thyroid cancer burden in this population remain limited, indicating a need for more thorough investigation.

The incidence of thyroid cancer in women varies significantly around the world, with up to a 15-fold difference in incidence between regions.^[10,11]^ Delayed cancer screening and treatment in areas with inadequate healthcare resources may exacerbate the disease burden. While global thyroid cancer mortality is generally declining, trends differ across countries.^[12]^ Women in developing countries may face heightened cancer risks due to rapid urbanization, unhealthy lifestyles, and environmental pollution. Over the past decades, thyroid cancer incidence has demonstrated region-specific patterns.^[13,14]^ Therefore, a detailed study of the WCBA thyroid cancer burden across different regions and countries is crucial for developing effective prevention and control strategies.

The GBD framework offers a unique opportunity to analyze trends in disease burden over the past 30 years, systematically assessing cancer burden across various development levels, regions, and countries and territories. Given the importance of WCBA for reproductive health and family planning, targeted interventions for this age group could significantly improve global women’s health. Consequently, there is a pressing need to thoroughly examine and analyze the disease burden and trends among WCBA thyroid cancer patients. Using the latest data from the GBD 2021 study, we investigated the incidence, prevalence, disability-adjusted life years (DALYs), and mortality of WCBA thyroid cancer at global, regional, and national levels from 1990 to 2021, providing crucial insights for developing prevention, screening, and treatment strategies that ultimately enhance reproductive and population health.

## 2. Materials and methods

### 2.1. Study population and data process

This study analyzed data on thyroid cancer in women from GBD 2021. GBD 2021 provides up-to-date epidemiological data on 371 diseases and injuries across 7 super-regions, 21 regions, and 204 countries and territories for the period 1990 to 2021. It also estimates the burden of 88 risk factors and assesses the strength of evidence in different regions.^[15]^ These data are freely available through the Global Data Outcomes platform at https://vizhub.healthdata.org/gbd-results/. The GBD 2021 program involved nearly 12,000 collaborators from 164 countries, who comprehensively assessed global health status and disease burden by collecting, reviewing, and analyzing extensive data. GBD 2021 classifies causes into 4 levels, ranging from level 1 (communicable, maternal, neonatal, and nutritional diseases) to level 4 (latent tuberculosis infection). Thyroid cancer is classified as a level 3 cause in GBD 2021.^[16]^ GBD 2021 also devises an assessment framework for all malignant neoplasms, benign neoplasms, and myeloproliferative and other hematopoietic neoplasms, excluding non-melanoma skin cancers. Cancer definitions and identifications are based on the International Classification of Diseases (ICD) codes, with ICD-9 and ICD-10 codes for thyroid cancer being 193 to 193.9 and C73, respectively.^[16]^ Additionally, according to the World Health Organization, WCBA was defined as those between 15 and 49 years old.^[17]^ This study utilized publicly available aggregated data from the GBD 2021 study, which does not contain any private or identifiable patient information. And, the study has been approved by the ethics committee of First Hospital of Handan City (2021-013).

Data sources for this study are available through the GBD 2021 Data Input Sources Tool, accessible via the Institute for Health Metrics and Evaluation website (https://ghdx.healthdata.org/gbd-2021/sources). The status of GBD data collection, modeling, analysis, and dissemination, as well as details on the thyroid cancer model, can be found on the webpage. In this study, we extracted estimates for the incidence, prevalence, DALYs, and deaths related to thyroid cancer in women aged 15 to 49 years (in 5-year age groups), along with their 95% uncertainty intervals. Incidence was defined as the number of new cases in the population, prevalence as the total number of cases, and deaths as the number of fatalities in the population. DALYs were calculated as the sum of years of life lost (YLL) and years lived with disability (YLD): DALY = YLL + YLD, where YLL = N × L (N = number of deaths; L = standard remaining life expectancy at the age of death from the GBD reference life table) and YLD = Σ(*P*_k_ × DW_k_) across sequelae (*P*_k_ = prevalence; DW_k_= disability weight), with multiplicative comorbidity adjustment.

The sociodemographic index (SDI), introduced by the Institute for Health Metrics and Evaluation in 2015, is a comprehensive measure used to assess the development levels of countries and regions. It emphasizes the relationship between social development and health outcomes. The SDI is calculated using the geometric mean of 3 indicators: fertility rates among women under 25, average years of education for individuals aged 15 and older, and lag-distributed income per capita, all scaled from 0 to 1. For the GBD 2021 study, these values were multiplied by 100, where an SDI of 0 indicates the minimum level of development, and an SDI of 100 indicates the maximum. Based on SDI estimates for 2021, 204 countries and territories were classified into 5 categories: low, low-middle, middle, high-middle, and high.^[16]^

### 2.2. Cross-country inequalities analysis

We employed a concentration index to measure the inequality in thyroid cancer burden among WCBA, specifically related to the SDI across different countries.^[18]^ The health inequality concentration index was calculated by ranking populations according to their SDI and burden of disease indicators, then fitting a Lorenz curve and integrating the area under the curve.^[19]^

### 2.3. Decomposition analysis

A decomposition analysis was conducted to identify the main factors driving changes in the burden of thyroid cancer among WCBA from 1990 to 2021. This analysis aimed to quantify the independent effects of population growth, aging, and epidemiological changes on the disease burden. The methodology involved assessing the contribution of each factor individually by holding the other factors constant.^[20]^

### 2.4. Estimated burden attributable to high body mass index (BMI)

Risk attribution followed the GBD comparative risk assessment framework. Per the GBD 2021 Risk Factors Collaborators, thyroid cancer had a single modeled risk-outcome pair – high body mass index (BMI) – therefore we report only BMI-attributable burden for this outcome.^[15]^. In GBD 2021, high BMI is defined as a BMI above the theoretical minimum risk exposure level of 20 to 25 kg/m² for adults aged ≥20 years. This risk-outcome inclusion was determined through systematic reviews of cohort evidence and assessment against established causal criteria.^[15]^

### 2.5. Data analysis

Between 1990 and 2021, we examined trends in the disease burden of WCBA thyroid cancer by analyzing incidence, prevalence, DALYs, mortality rates, and their age-standardized rates (ASRs). The ASRs, expressed per 1,00,000 WCBA, are detailed in a previous study.^[21]^ We used a Joinpoint regression model, with a maximum of 4 Joinpoints, to assess trends in thyroid cancer burden over time. This model identified significant trend changes, dividing the overall trend into segments, which were further evaluated using the annual percentage change and 95% confidence intervals (CI) to determine the epidemiologic trends in each segment.^[22]^ The Joinpoint regression analysis also provided the average annual percentage change (AAPC), reflecting the overall trend in disease burden across the entire study period. The statistical significance of annual percentage change and AAPC was determined based on whether their 95% CIs were entirely above or below 0. If the 95% CI included 0, the change in ASR was not considered statistically significant.^[23]^

Locally estimated scatterplot smoothing regression is a widely used nonparametric method ideal for fitting smooth curves to data, effectively modeling the relationship between 2 variables. This approach has been extensively utilized in previous GBD studies.^[24,25]^ In our study, we employed the locally estimated scatterplot smoothing method to analyze the relationship between disease burden indicators and SDI across GBD regions, with a particular focus on change trends from 1990 to 2021. The authors did not use generative AI or AI-assisted tools to write, analyze, or create content in this manuscript. This observational study was reported in accordance with the Strengthening the Reporting of Observational Studies in Epidemiology (STROBE) guideline. Data processing and analysis were performed using R version 4.3.0, along with Zstats software (www.medsta.cn/software).

## 3. Results

### 3.1. Global, regional, and national burden of thyroid cancer in WCBA

In 2021, approximately 67,558 new cases of thyroid cancer were reported globally among WCBA, with an ASR of 3.37 per 100,000. The total number of prevalent cases reached 6,11,351, with an ASR of 30.46 per 1,00,000. DALYs amounted to 2,06,508, and there were 3260 deaths, corresponding to ASRs of 10.38 and 0.16 per 1,00,000, respectively. Regarding SDI classification, both incidence and prevalence followed similar patterns, with the highest case numbers in the middle SDI group and the lowest in the low SDI group. However, the highest age-standardized incidence and prevalence rates were observed in both the high and low SDI groups. Although the number of DALYs and deaths was highest in the low-middle SDI group, the highest ASRs were found in the low SDI group.

Among the 7 GBD super-regions, the Southeast Asia, East Asia, and Oceania region recorded the most new thyroid cancer cases, while North Africa and the Middle East had the highest age-standardized incidence rates. The distribution of prevalence mirrored that of incidence. South Asia had the highest numbers of DALYs and deaths, with 84,402 and 1380 cases, respectively, and also had the highest age-standardized DALYs and deaths rates, at 17.31 and 0.29 per 1,00,000, respectively. Among the 21 GBD regions, South Asia reported the highest incidence and prevalence case counts, while high-income Asia Pacific showed the highest ASRs for both indicators. At the national level, India had the highest case numbers across all 4 thyroid cancer burden indicators in WCBA, while Cabo Verde had the highest ASRs (Tables 1, S1–S4, Supplemental Digital Content, https://links.lww.com/MD/R69).

**Table 1:** Incidence of WCBA thyroid cancer in 1990 and 2021, and its ASR estimated AAPC from 1990 to 2021 (global and regions).

Location	Number, 95% UI		Age-standardized incidence rate (per 1,00,000), 95% UI		AAPC of incidence rate, No. (95% CI)
	1990	2021	1990	2021	
Global	26,302 (23,183–30,421)	67,558 (55,974–83,415)	2.18 (1.93–2.51)	3.37 (2.78–4.16)	1.4 (1.32–1.48)*
SDI regions					
Low SDI	1532 (1079–2144)	6009 (4229–9441)	1.53 (1.08–2.14)	2.42 (1.71–3.77)	1.44 (1.18–1.69)*
Low-middle SDI	3538 (2712–4875)	13,681 (10,073–19,367)	1.44 (1.11–1.97)	2.8 (2.07–3.93)	2.13 (1.89–2.36)*
Middle SDI	6410 (5165–7995)	22,327 (17,413–27,878)	1.66 (1.34–2.06)	3.38 (2.63–4.23)	2.33 (2.22–2.44)*
High-middle SDI	6462 (5517–7414)	12,094 (9893–15,317)	2.5 (2.14–2.87)	3.34 (2.72–4.25)	0.92 (0.63–1.2)*
High SDI	8327 (7657–9027)	13,395 (12,062–15,305)	3.53 (3.24–3.82)	4.75 (4.26–5.44)	1.02 (0.79–1.24)*
GBD super regions					
Central Europe, Eastern Europe, and Central Asia	2992 (2767–3252)	3587 (3203–4022)	2.9 (2.69–3.16)	3.03 (2.71–3.41)	−0.11 (−0.92 to 0.71)
High-income	8293 (7618–9029)	11,367 (10,450–12,453)	3.4 (3.12–3.7)	4.06 (3.73–4.45)	0.57 (0.2–0.94)*
Latin America and Caribbean	1191 (1093–1307)	4003 (3489–4618)	1.41 (1.29–1.54)	2.42 (2.11–2.8)	1.83 (1.52–2.13)*
North Africa and Middle East	1634 (1172–2416)	8156 (6023–10,847)	2.48 (1.79–3.67)	5.06 (3.74–6.73)	2.34 (2.19–2.48)*
South Asia	3674 (2742–5175)	16,419 (11,890–23,304)	1.53 (1.15–2.15)	3.38 (2.45–4.78)	2.54 (2.13–2.96)*
Southeast Asia, East Asia, and Oceania	7325 (5342–8974)	19,920 (15,182–26,806)	1.84 (1.35–2.25)	3.4 (2.58–4.58)	2.02 (1.87–2.17)*
Sub-Saharan Africa	1194 (859–1632)	4107 (2783–6843)	1.23 (0.89–1.67)	1.62 (1.11–2.67)	0.85 (0.66–1.04)*
GBD regions					
Central Asia	286 (240–341)	486 (395–593)	2.07 (1.74–2.48)	1.93 (1.57–2.35)	−0.58 (−1.56 to 0.42)
Central Europe	1135 (971–1306)	943 (778–1112)	3.5 (3–4.03)	2.9 (2.39–3.43)	−0.49 (−1.22 to 0.24)
Eastern Europe	1571 (1427–1757)	2157 (1841–2536)	2.77 (2.51–3.1)	3.58 (3.05–4.22)	0.42 (−0.84 to 1.69)
Australasia	149 (104–210)	317 (212–460)	2.72 (1.9–3.82)	3.87 (2.58–5.62)	1.25 (0.04–2.48)*
High-income Asia Pacific	1797 (1414–2268)	2689 (2086–3508)	3.67 (2.88–4.64)	5.57 (4.31–7.34)	1.51 (0.96–2.07)*
High-income North America	2483 (2293–2696)	4256 (3912–4630)	3.21 (2.97–3.49)	4.6 (4.23–5.01)	1.13 (0.18–2.08)*
Southern Latin America	249 (187–327)	457 (339–608)	2.09 (1.57–2.73)	2.46 (1.82–3.28)	0.69 (−0.29 to 1.68)
Western Europe	3614 (3136–4174)	3648 (3115–4284)	3.61 (3.13–4.17)	3.23 (2.75–3.81)	−0.33 (−0.85 to 0.18)
Andean Latin America	135 (94–192)	644 (420–968)	1.71 (1.2–2.41)	3.72 (2.43–5.58)	2.72 (1.79–3.67)*
Caribbean	142 (113–178)	288 (217–377)	1.73 (1.38–2.17)	2.34 (1.76–3.07)	0.98 (0.44–1.52)*
Central Latin America	541 (478–618)	1993 (1650–2382)	1.59 (1.41–1.81)	2.86 (2.37–3.42)	1.87 (1.6–2.14)*
Tropical Latin America	373 (327–428)	1078 (951–1238)	1.08 (0.95–1.24)	1.64 (1.44–1.88)	1.33 (1.12–1.55)*
East Asia	4777 (3411–6166)	10,949 (7890–16,774)	1.63 (1.16–2.09)	2.8 (2.01–4.31)	1.8 (1.59–2.02)*
Oceania	12 (7–20)	36 (19–66)	0.93 (0.52–1.5)	1.09 (0.57–2.01)	0.5 (0.33–0.67)*
Southeast Asia	2536 (1737–3274)	8935 (6220–12,134)	2.45 (1.7–3.13)	4.67 (3.25–6.36)	2.1 (1.85–2.35)*
Central Sub-Saharan Africa	41 (21–76)	148 (69–296)	0.41 (0.21–0.75)	0.54 (0.26–1.08)	0.88 (0.72–1.04)*
Eastern Sub-Saharan Africa	880 (591–1259)	3141 (2009–5644)	2.33 (1.57–3.33)	3.23 (2.08–5.78)	1.06 (0.99–1.13)*
Southern Sub-Saharan Africa	158 (116–212)	392 (271–571)	1.42 (1.05–1.9)	1.86 (1.29–2.7)	0.87 (0.17–1.57)*
Western Sub-Saharan Africa	116 (78–168)	426 (278–664)	0.32 (0.22–0.45)	0.41 (0.27–0.63)	0.81 (0.74–0.89)*

AAPC = average annual percentage change, ASR = age-standardized incidence rate, BMI = body mass index, CI = confidence interval, DALY = disability-adjusted life year, GBD = global burden of disease, SDI = sociodemographic index, UI = uncertainty interval, WCBA = women of childbearing age.

* *P* < .05.

### 3.2. Global, regional, and national trends WCBA thyroid cancer burden

**Figure 1.**
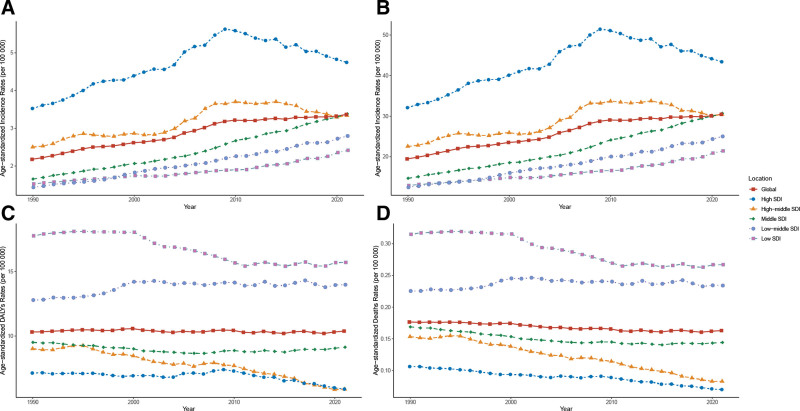
Changes in age-standardized incidence (A), prevalence (B), DALYs (C), and mortality (D) rates of thyroid cancer in WCBA at different SDI levels, 1990 to 2021. DALYs = disability-adjusted life years, SDI = sociodemographic index, WCBA = women of childbearing age.

Globally, the age-standardized incidence rate of thyroid cancer in WCBA significantly increased between 1990 and 2021 (AAPC = 1.4, 95% CI: 1.32–1.48). Similarly, the ASR for prevalence also trended upward (AAPC = 1.44, 95% CI: 1.37–1.52). In contrast, the age-standardized DALYs rate showed no significant change (AAPC = −0.01, 95% CI: −0.18 to 0.17), while the age-standardized mortality rate significantly decreased (AAPC = −0.28, 95% CI: −0.40 to −0.16). From an SDI perspective, age-standardized incidence and prevalence rates increased across all 5 SDI categories, with the most significant rise observed in the middle SDI category. Conversely, age-standardized DALYs and mortality rates generally declined across SDI categories, except at the low-middle SDI level, where DALYs increased significantly, though mortality rates remained stable (Fig. 1 and Tables 1, S1−S4, Supplemental Digital Content, https://links.lww.com/MD/R69).

**Figure 2.**
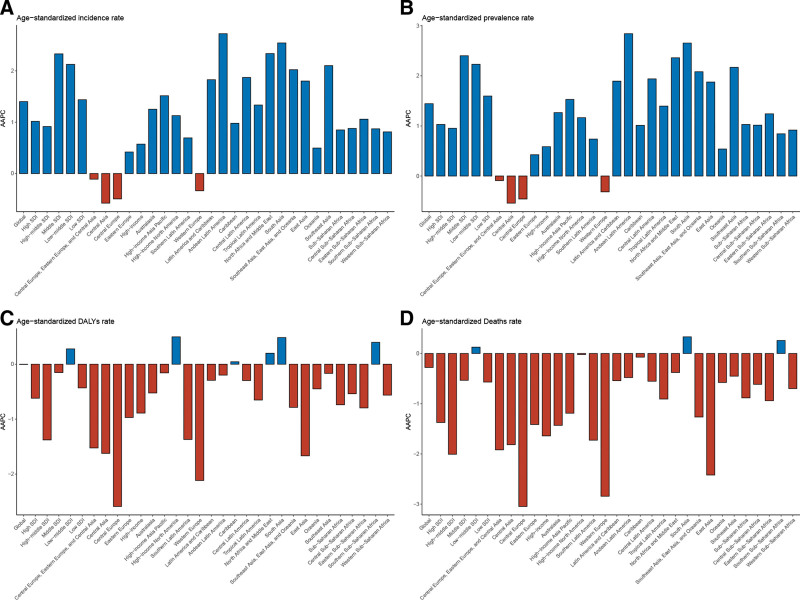
AAPC of age-standardized incidence (A), prevalence (B), DALYs (C), and mortality (D) rates of WCBA thyroid cancer, globally and regionally, 1990 to 2021. AAPC = average annual percentage change, DALYs = disability-adjusted life years, WCBA = women of childbearing age.

In the 7 GBD super regions, age-standardized incidence and prevalence rates followed similar trends. In Central Europe, Eastern Europe, and Central Asia, these rates remained stable, while they increased across the other 6 super regions. Age-standardized DALYs showed no significant change in North Africa and the Middle East, but increased in South Asia and decreased in other regions. Age-standardized mortality rates significantly decreased across 6 super regions, except in South Asia, where no significant change was observed. Among the 21 GBD regions, age-standardized incidence and prevalence rates increased significantly in all regions except Central Asia, Central Europe, Eastern Europe, Southern Latin America, and Western Europe. For age-standardized DALYs, no significant changes were observed in 7 regions, including North Africa and the Middle East, while South Asia and high-income North America experienced significant increases, with other regions showing significant decreases. Regarding age-standardized mortality rates, 4 regions (South Asia, the Caribbean, high-income North America, and Southern Sub-Saharan Africa) saw no significant changes, while other regions experienced significant declines. At the national level, Cabo Verde showed the largest increase in age-standardized prevalence, incidence, DALYs, and mortality, while Switzerland had the largest decrease (Fig. 2, Tables 1, S1–S4, Supplemental Digital Content, https://links.lww.com/MD/R69).

### 3.3. Correlation analysis of ASRs, AAPC, and SDI in WCBA thyroid cancer

**Figure 3.**
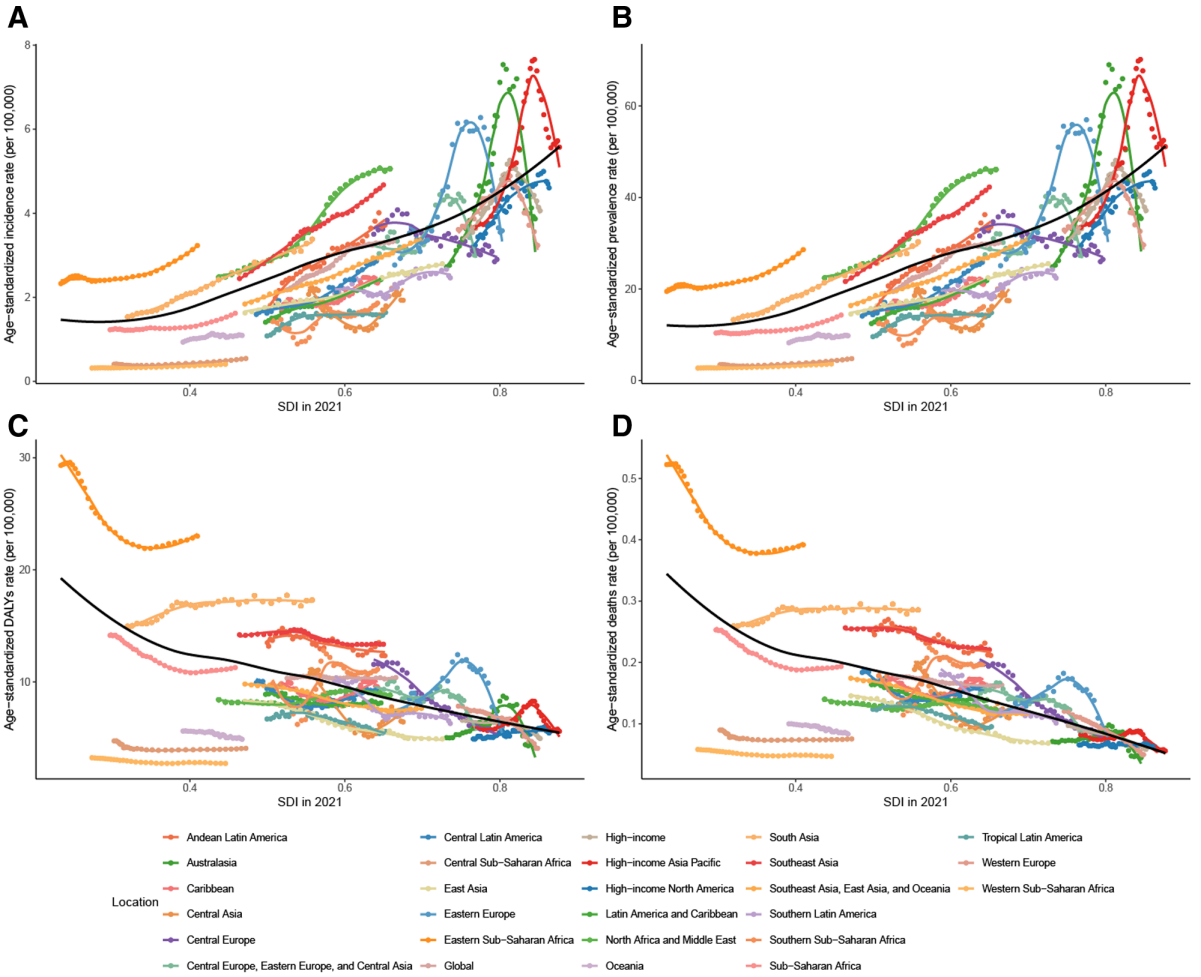
LOESS regression analysis of age-standardized incidence (A), prevalence (B), DALYs (C), and mortality (D) rates of thyroid cancer with SDI for WCBA in the GBD regions. DALYs = disability-adjusted life years, GBD = Global Burden of Diseases, LOESS = locally estimated scatterplot smoothing, SDI = sociodemographic index, WCBA = women of childbearing age.

In 2021, age-standardized incidence and prevalence rates of WCBA thyroid cancer at both the regional and national levels increase with increasing SDI, while ASRs of DALYs and mortality tend to decrease. From 1990 to 2021, age-standardized incidence and prevalence rates of WCBA thyroid cancer in the GBD super-regions and regions both increased with SDI, while ASRs of DALYs and mortality trended downward. Regionally, most regions showed a consistent upward trend, except for high-income Asia Pacific, Australasia, and Eastern Europe, which showed an inverted U-shaped distribution of age-standardized incidence and prevalence rates over time. For age-standardized DALYs rates and mortality rates, these 3 regions continued to show an inverted U-shaped distribution, while ASRs showed a decreasing trend in most other regions. In 2021, among the GBD super-regions and territories, the AAPC of ASRs showed a wavy distribution with increasing SDI, peaking when the SDI was about 0.6 and then decreasing with increasing SDI when the SDI is about 0.8 the ASRs are lowest and then rise again. At the national level, the fitted curves tend to increase and then decrease as the SDI increases. ASRs for age-standardized incidence and prevalence peaked at an SDI of 0.53 and then declined. In contrast, ASRs for DALYs and mortality rose less and declined more rapidly after peaking (Figs. 3 and S1−S4, Supplemental Digital Content, https://links.lww.com/MD/R69).

### 3.4. Decomposition analysis of incidence, prevalence, DALYs and deaths

Decomposition analyses revealed significant regional variations in the contributions of population aging, population growth, and epidemiological changes to the incidence, prevalence, DALYs, and mortality of WCBA thyroid cancer from 1990 to 2021. Globally, the relative increases in incidence and prevalence were primarily driven by epidemiological changes and population growth, with increases in both metrics exceeding 150%. As SDI levels rose, the relative increases in these indicators declined, while the contribution of population aging became more pronounced at higher SDI levels. Most regions exhibited positive growth in both indicators, with the highest relative growth observed in North Africa and the Middle East. Despite the negative growth in epidemiological changes, the overall values for DALYs and mortality remained positive globally, largely due to population growth. The overall relative changes were closely associated with SDI levels, with a downward trend in DALYs and mortality growth observed at both the high-middle and high SDI levels. In most regions, total relative changes increased, with population growth being the primary driving factor, while epidemiological changes contributed negatively (Fig. S4 and Table S5, Supplemental Digital Content, https://links.lww.com/MD/R69).

### 3.5. Socioeconomic inequality in the burden of WCBA thyroid cancer

From 1990 to 2021, the concentration index for age-standardized incidence and prevalence of thyroid cancer among WCBA decreased from 0.22 (95% CI: 0.18–0.25) and 0.23 (95% CI: 0.19–0.27) to 0.14 (95% CI: 0.10–0.18) and 0.14 (95% CI: 0.10–0.18), respectively. This trend suggests a reduction in inequities related to thyroid cancer incidence and prevalence in this population over the examined period. In contrast, the concentration indices for age-standardized DALYs and mortality were −0.14 (95% CI: −0.19 to −0.10) and −0.16 (95% CI: −0.21 to −0.11) in 1990, which increased to −0.18 (95% CI: −0.22 to −0.13) and −0.21 (95% CI: −0.26 to −0.16) by 2021. These changes indicate a growing inequity and a higher disease burden among the low SDI population (Fig. S5, Supplemental Digital Content, https://links.lww.com/MD/R69).

### 3.6. WCBA thyroid cancer burden attributable to high BMI

**Figure 4.**
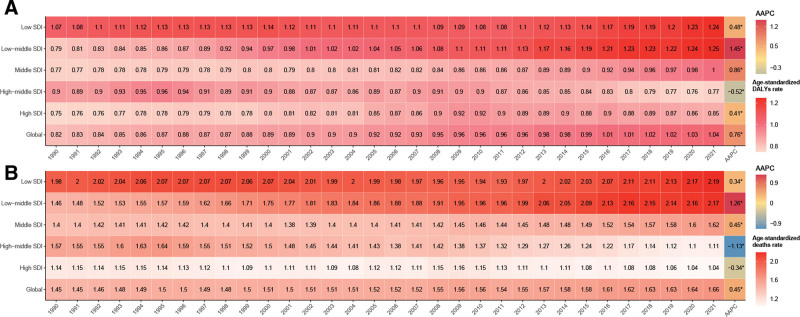
AAPC of age-standardized DALYs (A) and deaths (B) rates for WCBA thyroid cancer attributable to high BMI, 1990 to 2021. AAPC = average annual percentage change, BMI = body mass index, DALYs = disability-adjusted life years, WCBA = women of childbearing age.

From 1990 to 2021, age-standardized DALYs and mortality rates for WCBA thyroid cancer attributable to high BMI increased significantly (Fig. S6, Supplemental Digital Content, https://links.lww.com/MD/R69). The AAPC for DALYs was 0.76 (*P* < .05), while for mortality, it was 0.45 (*P* < .05). Over the past 3 decades, low-SDI regions exhibited the highest burden of age-standardized DALYs, whereas low-middle SDI regions experienced the fastest growth, with an AAPC of 1.45. Conversely, high-middle SDI regions showed the lowest growth in DALYs, with an AAPC of −0.52. The trend in mortality rates mirrored that of DALYs, with low-SDI regions showing the highest age-standardized mortality rates attributable to high BMI, coupled with the highest growth rate of 2.19. In contrast, mortality rates in high-middle and high SDI regions trended downward, with AAPCs of −1.13 and −0.34, respectively. Overall, the burden of thyroid cancers in WCBA attributable to high BMI was inversely related to SDI level, with lower SDI levels experiencing greater burdens (Fig. 4).

## 4. Discussion

In this study, we systematically assessed the incidence, prevalence, DALYs, and mortality of thyroid cancer among WCBA at global, regional, and national levels from 1990 to 2021, along with the trends in their ASRs. The findings indicate that, despite minimal global changes in DALYs and a slight decline in mortality, both incidence and prevalence showed an upward trend, with significant regional and SDI-level heterogeneity. Although disparities in incidence and prevalence have narrowed, the disease burden remains substantially higher in low-SDI regions, particularly where the impact of high BMI has intensified most rapidly.

Consistent with previous research, thyroid cancer incidence is rising across most regions.^[4,26,27]^ Traditional risk factors, such as ionizing radiation exposure, family history, and long-term hypothyroidism, are pivotal in the pathogenesis of WCBA thyroid cancer.^[28]^ Our decomposition analysis revealed that changes in prevalence significantly contributed to the increasing incidence and prevalence of thyroid cancer. Additionally, lifestyle changes and environmental shifts linked to rapid social development have been implicated in the rising rates of thyroid cancer.^[29,30]^ While earlier studies have identified specific risk factors for thyroid cancer in women, limitations in the strength of evidence and statistical power underscore the need for further research to explore these variables in greater depth for targeted early prevention.^[29,31,32]^

Socioeconomic development has led to increased thyroid cancer screening, with improvements in routine surgical exams and cancer registries contributing to the earlier detection and rising incidence of thyroid cancer in women. Some studies attribute this rise primarily to the increased detection of asymptomatic, small, and subclinical papillary carcinomas.^[33,34]^ Others suggest that the surge in thyroid cancer cases in recent years may be due to overdiagnosis.^[26,35]^ As a result, professional organizations have recommended caution when performing biopsies on small thyroid nodules unless imaging indicates malignant potential.^[36–39]^ Additionally, the global increase in WCBA obesity rates alters inflammatory, metabolic, and hormonal pathways, which may also contribute to this rise.^[40,41]^

Although the concurrent rise in incidence and prevalence, alongside a modest decline in mortality, aligns with improved detection and management, overdiagnosis likely contributes to the increasing incidence of thyroid cancer.^[42,43]^ However, due to the ecological study design and absence of staging or treatment data, we cannot establish causality for early diagnosis. Increased diagnostic intensity may result in overdiagnosis and longer survival without necessarily reducing mortality, whereas advances in risk-adaptive therapy can lower mortality independent of shifts in diagnostic stage.^[38]^ Therefore, we avoid inferring causal relationships and instead describe the influence of SDI on early diagnosis. We recommend integrating risk-stratified clinical case finding within reproductive healthcare rather than implementing population-wide screening for asymptomatic WCBA, alongside active monitoring for low-risk papillary microcarcinoma to reduce overdiagnosis.^[44,45]^ In high-SDI settings, emphasis should be placed on adherence to imaging and biopsy thresholds, multidisciplinary review, and standardized monitoring protocols; in low- and lower-middle-SDI settings, priorities include improving access to high-quality ultrasound and timely pathology services, integrating case detection into primary and maternal care, and establishing clear referral pathways.^[46,47]^ Across all SDI levels, primary prevention through promotion of healthy body weight, provider education, and registry-driven quality metrics can support timely and appropriate diagnosis while minimizing unnecessary procedures.^[48,49]^

It was also found that the age-standardized incidence and prevalence of WCBA thyroid cancer in some regions with higher SDI showed an inverted U-shaped distribution, possibly related to changes in diagnostic criteria. In contrast, in regions with lower SDI, DALYs and mortality rates were higher than global levels despite lower incidence and prevalence rates, suggesting that these countries may face greater challenges in healthcare resource allocation, disease management, and treatment.^[50,51]^

This study found that age-standardized DALYs and mortality rates of thyroid cancer in WCBA were negatively correlated with SDI. This contrasts with some studies, which may be due to this study’s focus on women of reproductive age.^[52,53]^ Additionally, the WCBA group may be more likely to access relevant screening, facilitating early detection and treatment.^[54]^ Over the past 30 years, age-standardized DALYs and mortality rates of thyroid cancer in WCBA have decreased significantly in most regions, but in some, such as the low-middle SDI subgroup, a significant increase has been observed, likely related to lifestyle changes and declining environmental quality due to rapid urbanization and industrialization. In South Asia and Southern Sub-Saharan Africa, inadequate healthcare services may also contribute to the rise in ASRs in these regions.^[3,55]^

This study found that the major changes in DALYs and deaths of thyroid cancer in WCBA were caused by population growth. Population growth for the WCBA thyroid cancer population is an issue that cannot be ignored, as thyroid cancer is one of the most common cancers in this group.^[56]^ Despite its relatively good prognosis, thyroid cancer carries a psychological and economic burden that exceeds even that of other malignant cancers.^[57–59]^ Therefore, as a key group for future population growth, WCBA plays an important role in society and the economy, especially in regions and countries with low SDI.

Pregnancy is a critical consideration for the WCBA group. While surgical treatment generally does not significantly impact maternal and infant health or future fertility, hormonal changes during pregnancy may influence the progression of thyroid cancer. Therefore, close monitoring is essential to minimize potential effects on the fetus.^[6,60]^ Research indicates that the fertility of women with thyroid cancer following radioiodine treatment is comparable to that of women without the disease, with little association with recurrence or adverse pregnancy outcomes.^[61–63]^ However, treated WCBA patients with thyroid cancer tend to experience delayed childbearing during their reproductive years, often linked to specific syndromes, compared to the general population. Consequently, for women with thyroid cancer, both pre-pregnancy and during pregnancy, vigilant clinical monitoring, disease progression control, and timely treatment adjustments are crucial.

In recent years, the disease burden associated with high BMI has become increasingly severe, particularly concerning cancer.^[15,64]^ This study identified a significant increase in the burden of WCBA thyroid cancer attributable to high BMI, especially in regions with low and low-medium SDI levels. The AAPC for DALYs and mortality rates rose considerably in these regions, while it decreased in areas with higher SDI. Given the complex, nonlinear relationship between high BMI and SDI, SDI can only partially explain the temporal variations in the burden related to high BMI.^[65]^ Hence, cross-sectoral efforts are essential to prevent excessive weight gain in WCBA.^[66]^ Simultaneously, prevention and management strategies targeting high BMI should be reinforced, particularly in low SDI regions, to reduce the associated disease burden.

Although our analysis is based on the latest GBD 2021 data and methodologies, there are inherent limitations. First, the original data from GBD 2021 do not cover all countries or regions globally, and the subtypes of thyroid cancer cases were not distinguished, which may lead to varying contributions to the overall disease burden. Additionally, GBD’s risk factor identification relies heavily on reviews rather than prospective studies. Lastly, variations in diagnostic and testing protocols for thyroid cancer across different countries and over time may affect the comparability of the results. To address these limitations, future studies should develop and implement multiple methodologies to further validate the findings of this research.

## 5. Conclusion

In summary, thyroid cancer among WCBA represents a significant global public health challenge. Despite a global decline in age-standardized DALY rates and mortality for WCBA thyroid cancer between 1990 and 2021, the incidence and prevalence have continued to rise, with marked regional disparities. Notably, the impact of high BMI on the thyroid cancer burden in the WCBA population warrants particular attention. Therefore, it is essential to develop targeted preventive measures and health management strategies tailored to the characteristics of different populations and regions.

## Acknowledgments

We thank the Global Burden of Diseases, Injuries, and Risk Factors Study for sharing such high-value data for free.

## Author contributions

**Data curation**: Yangfan Du.

**Formal analysis**: Yi Shen.

**Investigation**: Linlin Wang, Shuai Jin.

**Supervision**: Hongmei Liu, Song Dong.

**Visualization**: Jia Liu.

**Validation**: Xiaojing Li.

**Writing** – **original draft**: Hongzhou Liu, Lang Xie.

## Supplementary Material
