# Supplementary material for: Burden and trends of thyroid cancer among women of childbearing age, 1990–2021: A serial cross-sectional analysis
Source: Medicine (Baltimore). 2026 May 12;104(49):e45930. doi: 10.1097/MD.0000000000045930 (PMC12688739; doi:10.1097/MD.0000000000045930)
Supplement: Supplementary file 1 [file medi-104-e45930-s001.pdf]

Figure S1: Age-standardized rates of incidence, prevalence, DALYs, and mortality of WCBA thyroid cancer by SDI, 2021 (global and regions).

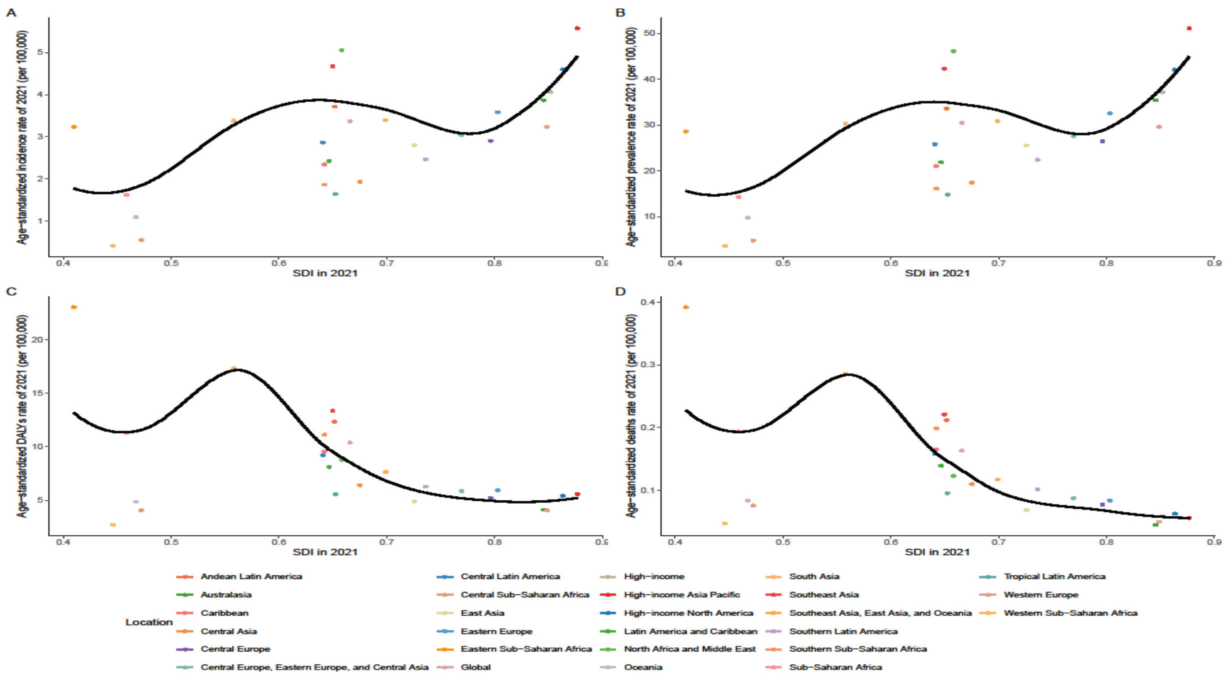

Figure S2: Age-standardized rates of incidence, prevalence, DALYs, and mortality of WCBA thyroid cancer by SDI, 2021 (countries and territories).

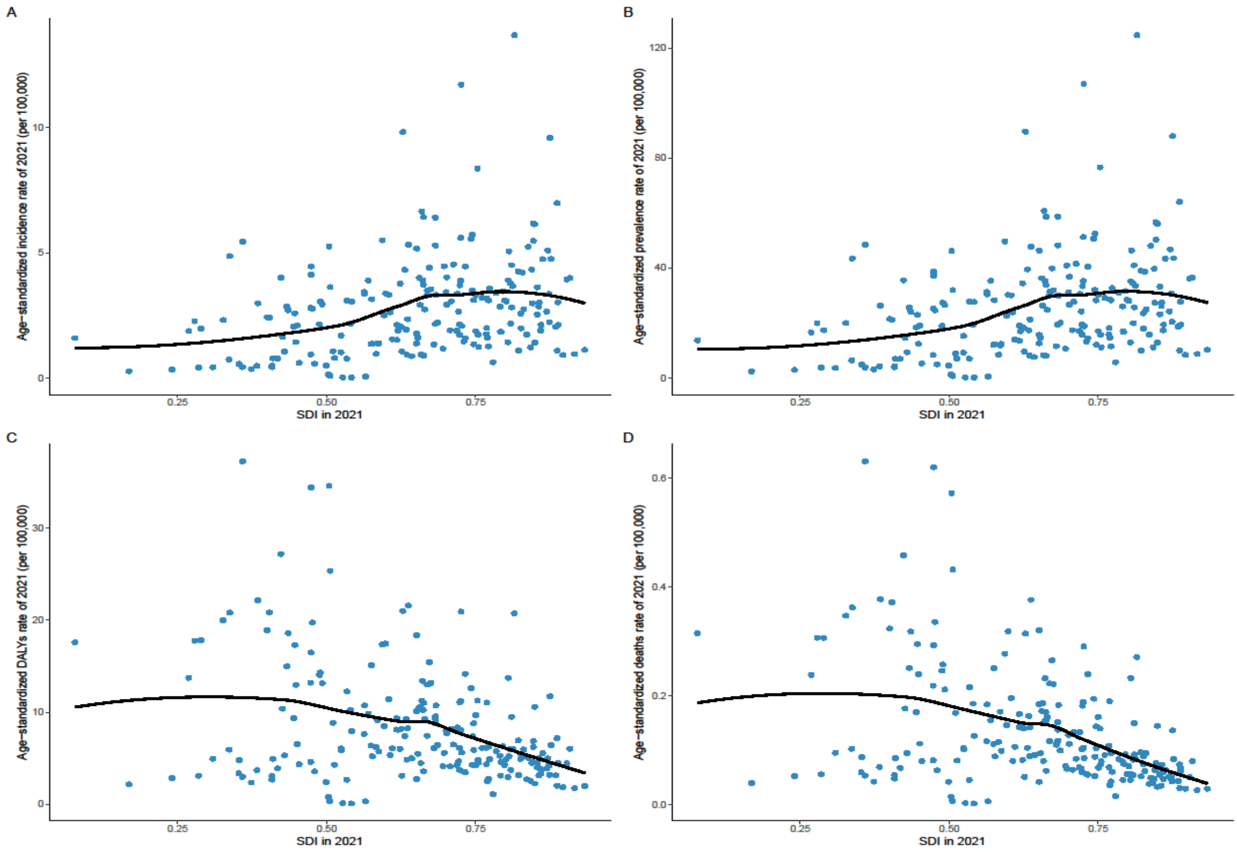

Figure S3: Association of AAPC with SDI (2021) for age-standardized incidence, prevalence, DALYs, and mortality (global and regions).

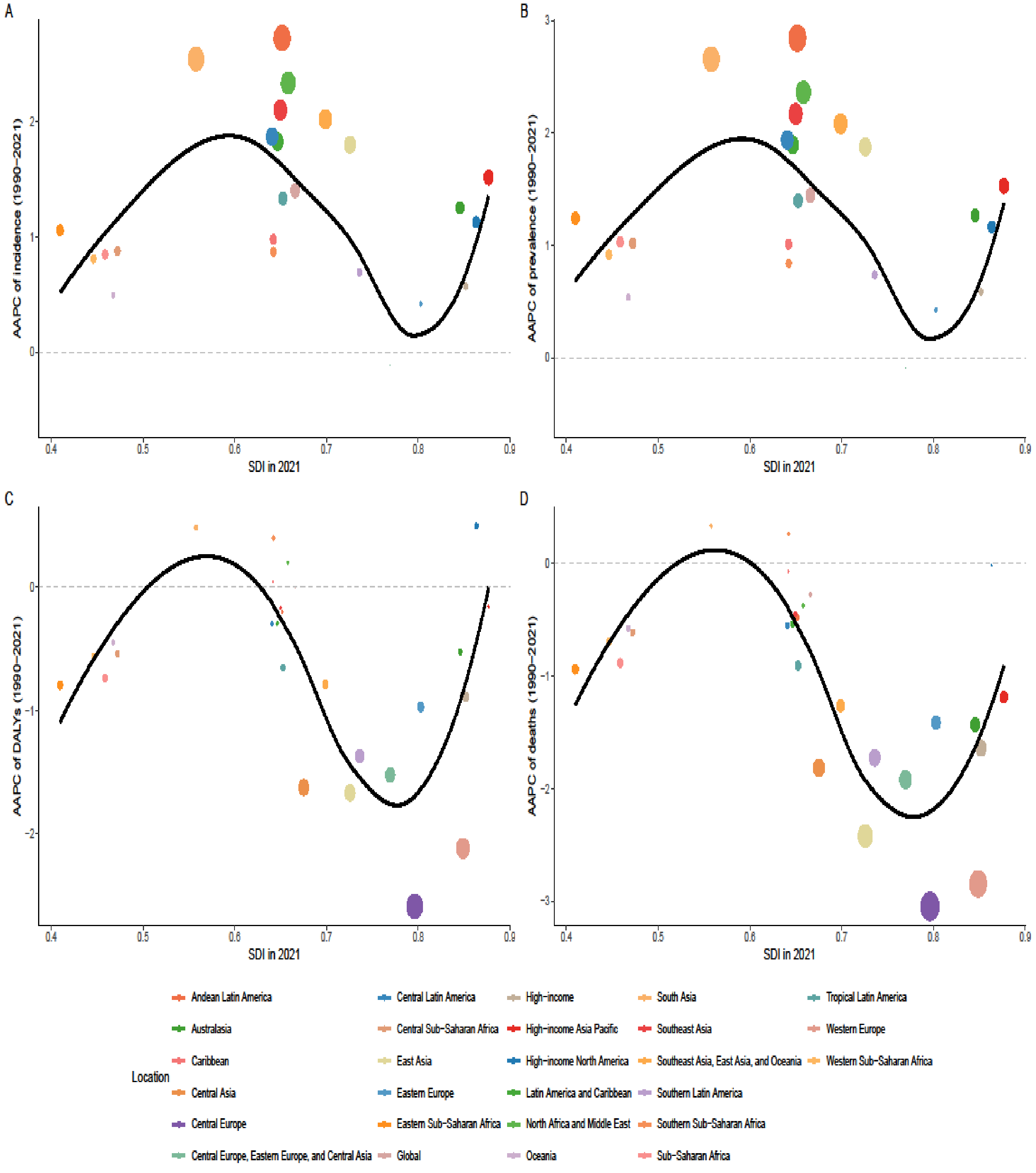

Figure S4: Association of AAPC with SDI (2021) for age-standardized incidence, prevalence, DALYs, and mortality (countries and territories).

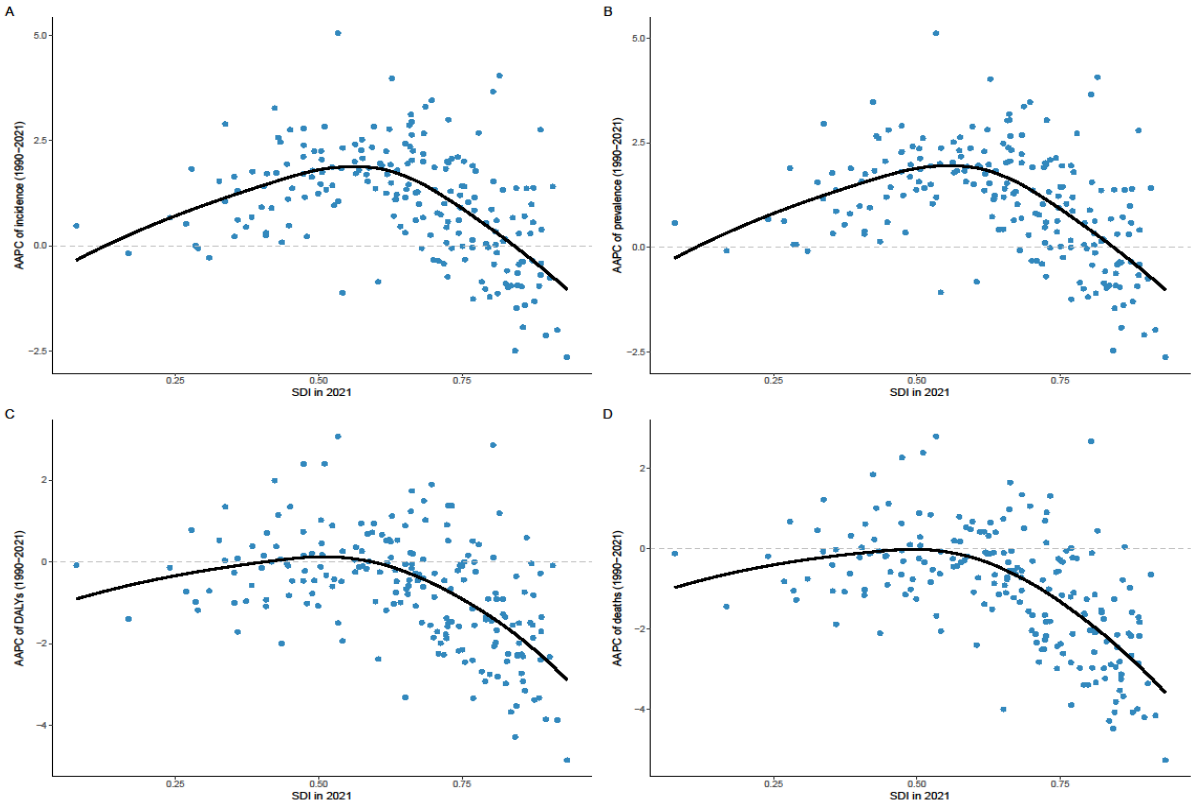

Figure S5: Changes in WCBA thyroid cancer incidence, prevalence, DALYs, and deaths according to population-level determinants of aging, population growth, and epidemiological change from 1990 to 2021.

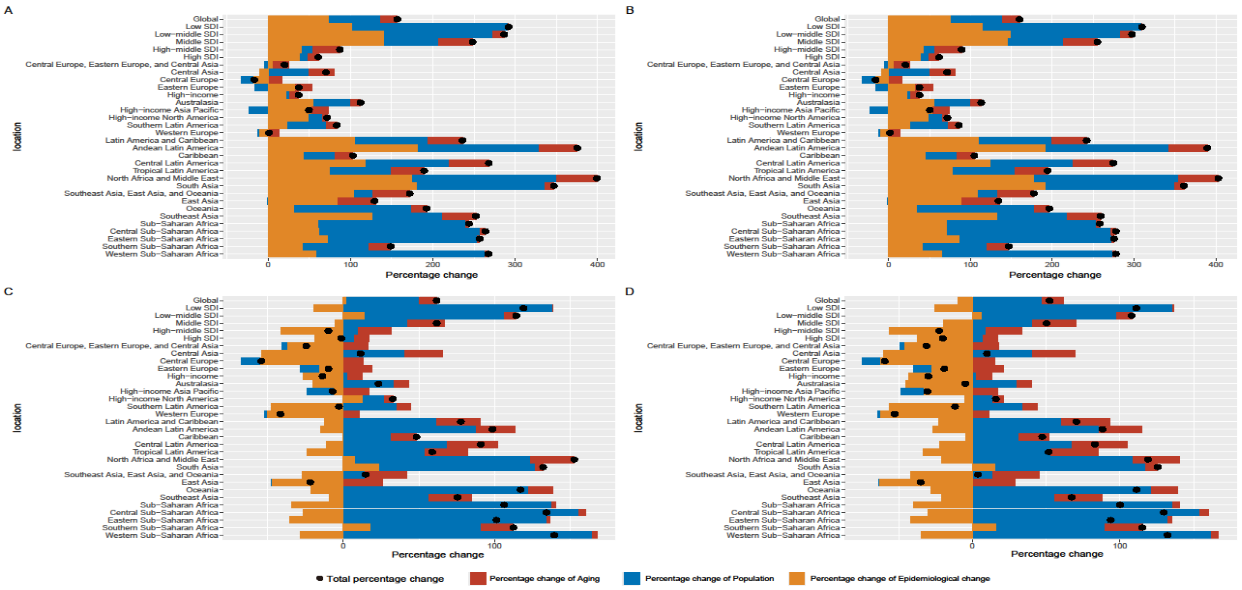

Figure S6: The concentration index for incidence, prevalence, DALYs and deaths of WCBA thyroid cancer worldwide in 1990 and 2021.

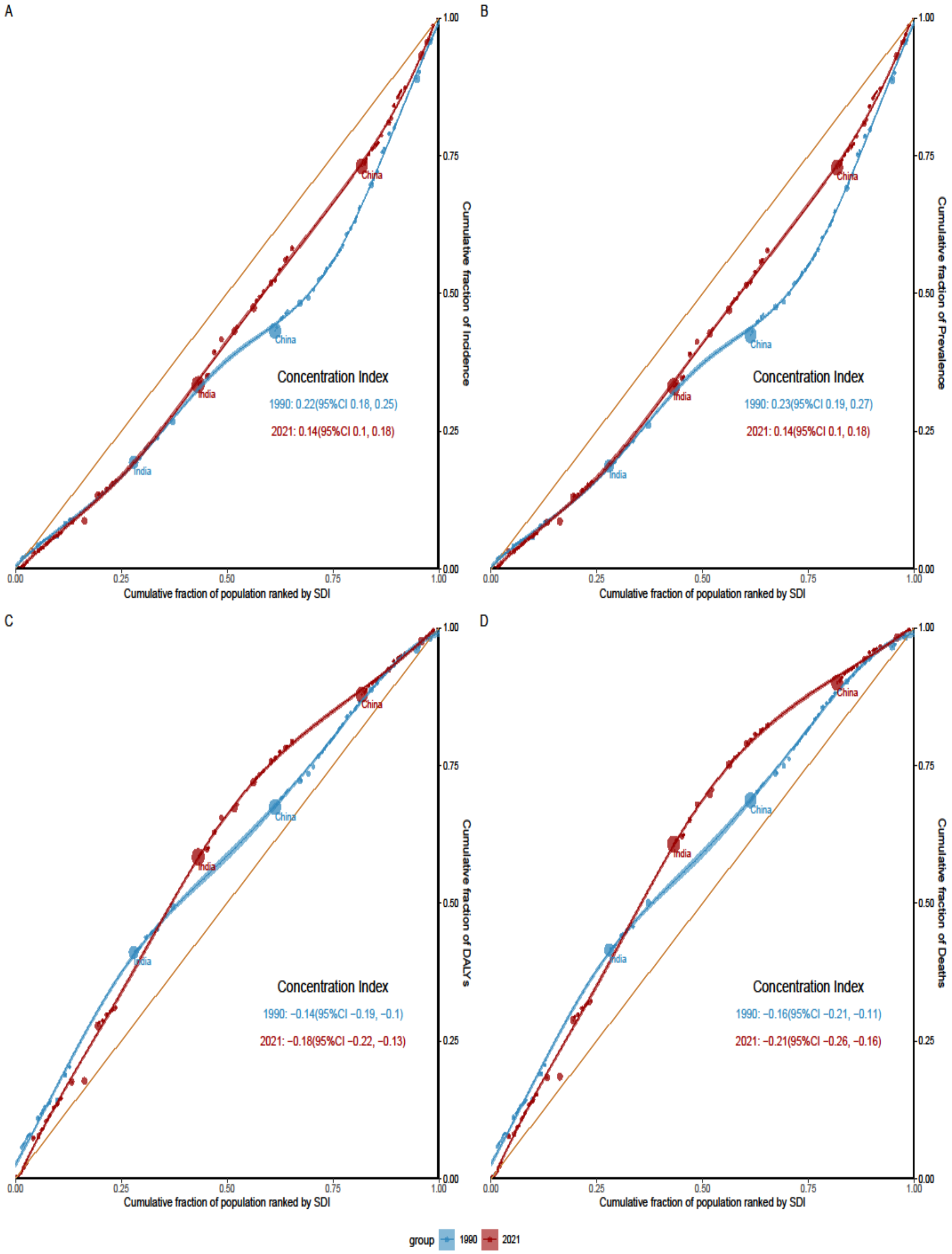

Table S1: Incidence of WCBA thyroid cancer in 1990 and 2021, and its ASR estimated AAPC from 1990 to 2021 (countries and territories).

Location	Number, 95% UI		Age-standardized incidence rate (per 100 000), 95% UI		AAPC of incidence rate, No. (95% CI)
	1990	2021	1990	2021	
Armenia	14 (9 to 21)	25 (16 to 36)	1.83 (1.18 to 2.7)	2.93 (1.86 to 4.34)	1.34 (0.36 to 2.33)*
Azerbaijan	17 (9 to 29)	34 (17 to 63)	1.12 (0.61 to 1.92)	1.17 (0.59 to 2.15)	0.26 (-0.46 to 0.99)
Georgia	29 (18 to 45)	41 (27 to 62)	2.18 (1.38 to 3.31)	4.46 (2.85 to 6.72)	2.07 (0.21 to 3.97)*
Kazakhstan	157 (118 to 205)	202 (144 to 275)	4.22 (3.17 to 5.53)	3.89 (2.76 to 5.3)	-0.74 (-2.88 to 1.45)
Kyrgyzstan	36 (23 to 54)	56 (34 to 85)	3.86 (2.5 to 5.78)	3.32 (2.04 to 5.06)	-0.86 (-2.51 to 0.83)
Mongolia	5 (3 to 10)	19 (10 to 34)	1.45 (0.73 to 2.56)	2.12 (1.13 to 3.68)	1.25 (0.29 to 2.22)*
Tajikistan	0 (0 to 0)	0 (0 to 1)	0.02 (0.01 to 0.04)	0.02 (0.01 to 0.03)	-1.11 (-1.58 to -0.64)*
Turkmenistan	13 (10 to 16)	27 (18 to 39)	1.81 (1.38 to 2.34)	2.15 (1.44 to 3.15)	0.67 (-2.12 to 3.54)
Uzbekistan	15 (9 to 22)	82 (51 to 124)	0.39 (0.25 to 0.59)	0.89 (0.56 to 1.35)	2.64 (1.93 to 3.35)*
Albania	10 (6 to 17)	13 (6 to 23)	1.42 (0.78 to 2.39)	1.91 (0.91 to 3.57)	0.78 (-0.09 to 1.65)
Bosnia and Herzegovina	18 (12 to 26)	15 (9 to 25)	1.59 (1.04 to 2.31)	1.72 (1.03 to 2.78)	0.08 (-0.59 to 0.75)
Bulgaria	39 (27 to 57)	34 (22 to 51)	1.7 (1.14 to 2.45)	1.75 (1.11 to 2.66)	0.14 (-1.54 to 1.85)
Croatia	55 (36 to 80)	34 (21 to 51)	4.22 (2.77 to 6.17)	3.1 (1.93 to 4.64)	-1.21 (-2.51 to 0.11)

Czechia	145 (98 to 210)	130 (82 to 197)	5.05 (3.39 to 7.31)	4.22 (2.63 to 6.4)	-0.59 (-1.64 to 0.47)
Hungary	120 (79 to 171)	81 (54 to 116)	4.15 (2.74 to 5.95)	2.86 (1.87 to 4.14)	-1.02 (-2.35 to 0.32)
Montenegro	6 (4 to 8)	6 (4 to 9)	3.66 (2.37 to 5.54)	3.46 (2.22 to 5.34)	-0.03 (-0.58 to 0.52)
North Macedonia	10 (6 to 14)	12 (7 to 19)	1.94 (1.27 to 2.76)	1.86 (1.13 to 3.02)	-0.01 (-0.82 to 0.81)
Poland	500 (384 to 641)	404 (298 to 536)	5.01 (3.85 to 6.43)	3.67 (2.71 to 4.87)	-1.13 (-2.74 to 0.51)
Romania	122 (81 to 176)	104 (66 to 156)	2.15 (1.42 to 3.11)	1.99 (1.25 to 3.01)	-0.04 (-1.25 to 1.18)
Serbia	38 (20 to 69)	45 (23 to 79)	1.53 (0.82 to 2.8)	1.84 (0.95 to 3.29)	0.55 (-0.07 to 1.17)
Slovakia	41 (23 to 68)	45 (23 to 79)	2.98 (1.66 to 4.93)	2.82 (1.44 to 5.08)	-0.16 (-0.89 to 0.57)
Slovenia	13 (9 to 19)	7 (4 to 10)	2.58 (1.73 to 3.7)	1.22 (0.74 to 1.87)	-2.49 (-3.18 to -1.8)*
Belarus	99 (63 to 148)	92 (54 to 142)	3.82 (2.43 to 5.73)	3.4 (2 to 5.28)	-0.86 (-5.33 to 3.83)
Estonia	19 (12 to 28)	10 (6 to 15)	4.59 (2.95 to 6.88)	2.99 (1.9 to 4.47)	-1.48 (-3.33 to 0.42)
Latvia	25 (16 to 37)	14 (9 to 20)	3.65 (2.35 to 5.41)	2.75 (1.71 to 4.11)	-0.98 (-2.74 to 0.8)
Lithuania	49 (32 to 71)	22 (14 to 32)	5.11 (3.32 to 7.5)	3.02 (1.93 to 4.47)	-1.93 (-3.26 to -0.58)*
Republic of Moldova	15 (12 to 20)	17 (12 to 23)	1.38 (1.04 to 1.82)	1.58 (1.14 to 2.14)	-0.05 (-0.84 to 0.74)
Russian Federation	1048 (984 to 1124)	1603 (1386 to 1828)	2.79 (2.62 to 3)	3.78 (3.27 to 4.32)	0.33 (-1.26 to 1.96)

Ukraine	316 (202 to 473)	400 (197 to 709)	2.38 (1.52 to 3.56)	3.21 (1.58 to 5.7)	1.04 (0.34 to 1.74)*
Australia	135 (91 to 195)	299 (194 to 439)	2.95 (1.97 to 4.26)	4.34 (2.81 to 6.39)	1.37 (0.09 to 2.67)*
New Zealand	14 (10 to 21)	19 (12 to 27)	1.6 (1.07 to 2.32)	1.41 (0.94 to 2.03)	-0.44 (-2.85 to 2.03)
Brunei Darussalam	2 (1 to 4)	6 (3 to 10)	3.71 (1.73 to 6.84)	4.51 (2.39 to 7.75)	0.6 (0.21 to 1)*
Japan	1418 (1092 to 1813)	1628 (1253 to 1985)	3.85 (2.96 to 4.92)	5.1 (3.96 to 6.15)	0.98 (0.66 to 1.31)*
Singapore	32 (21 to 46)	51 (33 to 76)	3.48 (2.29 to 5.03)	2.85 (1.82 to 4.26)	-0.38 (-0.97 to 0.21)
Republic of Korea	345 (185 to 649)	1003 (525 to 1749)	3.01 (1.63 to 5.65)	6.99 (3.61 to 12.29)	2.76 (1.89 to 3.64)*
Canada	304 (202 to 439)	317 (204 to 473)	3.93 (2.61 to 5.67)	3.36 (2.15 to 5.01)	-0.35 (-1.28 to 0.59)
Greenland	0 (0 to 1)	0 (0 to 1)	2.66 (1.25 to 4.78)	1.99 (0.92 to 4.08)	-0.9 (-1.34 to -0.45)*
United States of America	2178 (2027 to 2345)	3938 (3617 to 4288)	3.13 (2.91 to 3.37)	4.75 (4.36 to 5.17)	1.36 (0.37 to 2.37)*
Argentina	156 (104 to 227)	269 (170 to 405)	1.98 (1.31 to 2.87)	2.14 (1.36 to 3.23)	0.44 (-0.65 to 1.54)
Chile	74 (48 to 109)	158 (102 to 236)	2.26 (1.47 to 3.34)	3.1 (1.99 to 4.66)	1.15 (0.26 to 2.04)*
Uruguay	19 (12 to 28)	30 (19 to 45)	2.5 (1.63 to 3.78)	3.36 (2.13 to 5.04)	1 (0.4 to 1.61)*
Andorra	0 (0 to 1)	1 (0 to 1)	2.43 (1.1 to 4.57)	2.65 (1.19 to 4.96)	0.29 (-0.07 to 0.66)
Austria	103 (68 to 149)	86 (56 to 128)	4.94 (3.3 to 7.18)	3.62 (2.34 to 5.42)	-0.97 (-1.55 to -0.38)*

Belgium	68 (45 to 99)	74 (48 to 110)	2.65 (1.74 to 3.86)	2.53 (1.63 to 3.78)	-0.38 (-1.68 to 0.94)
Cyprus	5 (2 to 9)	8 (4 to 16)	2.44 (1.18 to 4.52)	1.73 (0.8 to 3.6)	-0.94 (-1.72 to -0.16)*
Denmark	25 (17 to 36)	13 (9 to 20)	1.72 (1.17 to 2.47)	0.91 (0.58 to 1.37)	-2.12 (-3.55 to -0.68)*
Finland	50 (34 to 72)	28 (17 to 41)	3.45 (2.33 to 4.98)	2.12 (1.32 to 3.16)	-1.4 (-2.87 to 0.09)
France	661 (445 to 942)	860 (540 to 1279)	4.42 (2.97 to 6.3)	5.24 (3.27 to 7.84)	0.67 (0.04 to 1.3)*
Germany	969 (661 to 1384)	792 (510 to 1164)	4.67 (3.19 to 6.68)	3.94 (2.52 to 5.8)	-0.76 (-1.16 to -0.35)*
Greece	48 (35 to 63)	53 (39 to 70)	1.8 (1.33 to 2.38)	1.85 (1.36 to 2.44)	0.05 (-0.82 to 0.93)
Iceland	5 (3 to 7)	4 (3 to 6)	8.06 (5.31 to 11.65)	4.76 (3.01 to 7.09)	-1.31 (-3.14 to 0.54)
Ireland	17 (11 to 24)	32 (20 to 48)	1.95 (1.28 to 2.83)	2.23 (1.42 to 3.34)	0.56 (-0.28 to 1.41)
Israel	27 (18 to 40)	52 (34 to 77)	2.33 (1.54 to 3.44)	2.18 (1.42 to 3.26)	0.03 (-2.48 to 2.59)
Italy	940 (711 to 1238)	832 (619 to 1076)	6.24 (4.72 to 8.22)	5.06 (3.76 to 6.55)	-0.44 (-1.24 to 0.37)
Luxembourg	3 (2 to 4)	4 (3 to 5)	2.7 (2.01 to 3.56)	2.04 (1.49 to 2.71)	-0.95 (-1.79 to -0.12)*
Malta	3 (2 to 4)	4 (2 to 5)	2.39 (1.55 to 3.51)	3.06 (1.95 to 4.66)	0.87 (-0.73 to 2.49)
Monaco	0 (0 to 0)	0 (0 to 1)	2.61 (1.29 to 4.79)	3.99 (1.82 to 7.53)	1.41 (1.22 to 1.6)*
Netherlands	75 (50 to 109)	87 (55 to 128)	1.82 (1.21 to 2.63)	2.12 (1.32 to 3.14)	0.38 (0.15 to 0.62)*

Norway	20 (15 to 26)	13 (10 to 18)	1.76 (1.33 to 2.32)	0.95 (0.69 to 1.27)	-2 (-3.87 to -0.09)*
Portugal	78 (52 to 113)	107 (70 to 159)	3.01 (1.99 to 4.35)	3.5 (2.27 to 5.24)	0.31 (-1 to 1.63)
San Marino	0 (0 to 0)	0 (0 to 1)	3.31 (1.65 to 5.76)	3.03 (1.2 to 5.84)	-0.41 (-0.68 to -0.15)*
Spain	210 (140 to 307)	208 (134 to 310)	2.2 (1.46 to 3.21)	1.6 (1.03 to 2.41)	-1.26 (-1.58 to -0.95)*
Sweden	31 (22 to 42)	27 (18 to 39)	1.3 (0.93 to 1.77)	1.09 (0.73 to 1.56)	-0.69 (-2.03 to 0.67)
Switzerland	49 (32 to 70)	27 (17 to 40)	2.51 (1.65 to 3.61)	1.12 (0.71 to 1.66)	-2.64 (-4.23 to -1.02)*
United Kingdom	226 (207 to 248)	333 (302 to 368)	1.52 (1.39 to 1.67)	1.9 (1.72 to 2.1)	0.71 (0.16 to 1.26)*
Bolivia (Plurinational State of)	29 (13 to 53)	100 (48 to 187)	2.24 (1.01 to 4.02)	3.37 (1.61 to 6.26)	1.36 (1.23 to 1.49)*
Ecuador	31 (21 to 45)	180 (106 to 287)	1.46 (0.98 to 2.12)	3.91 (2.3 to 6.22)	3.12 (1.59 to 4.67)*
Peru	75 (42 to 124)	363 (180 to 646)	1.67 (0.95 to 2.72)	3.73 (1.85 to 6.62)	2.94 (1.95 to 3.95)*
Antigua and Barbuda	0 (0 to 0)	1 (1 to 1)	2.35 (1.73 to 3.11)	3.32 (2.45 to 4.43)	1.43 (0.12 to 2.75)*
Bahamas	2 (1 to 2)	4 (3 to 6)	2.4 (1.78 to 3.15)	3.9 (2.59 to 5.58)	1.53 (0.83 to 2.23)*
Barbados	1 (1 to 2)	3 (2 to 4)	2.12 (1.57 to 2.78)	3.11 (2.07 to 4.51)	1.2 (0.44 to 1.97)*
Belize	0 (0 to 0)	2 (1 to 2)	0.77 (0.57 to 1.01)	1.55 (1.13 to 2.09)	1.91 (1.11 to 2.71)*
Bermuda	0 (0 to 1)	1 (0 to 1)	2.6 (1.64 to 3.91)	4.26 (2.55 to 6.69)	1.33 (0.69 to 1.98)*

Cuba	63 (41 to 92)	95 (60 to 146)	2.24 (1.46 to 3.26)	3.3 (2.08 to 5.07)	1.26 (0.37 to 2.16)*
Dominica	0 (0 to 0)	0 (0 to 0)	1.01 (0.58 to 1.65)	1.35 (0.68 to 2.39)	0.91 (0.58 to 1.24)*
Dominican Republic	17 (9 to 29)	53 (26 to 98)	1.11 (0.63 to 1.89)	1.89 (0.94 to 3.47)	1.7 (1.06 to 2.34)*
Grenada	0 (0 to 1)	1 (1 to 1)	2.73 (1.75 to 4.12)	3.5 (2.15 to 5.4)	0.61 (-0.46 to 1.69)
Guyana	1 (1 to 2)	3 (2 to 6)	0.84 (0.53 to 1.26)	1.77 (0.99 to 2.94)	2.43 (2.15 to 2.7)*
Haiti	17 (7 to 32)	48 (21 to 93)	1.3 (0.57 to 2.45)	1.43 (0.65 to 2.76)	0.48 (0.21 to 0.76)*
Jamaica	7 (5 to 10)	25 (14 to 42)	1.48 (0.98 to 2.18)	3.31 (1.87 to 5.45)	2.67 (1.79 to 3.57)*
Puerto Rico	19 (13 to 29)	22 (14 to 34)	2.03 (1.33 to 3.01)	2.58 (1.59 to 4)	0.16 (-0.71 to 1.04)
Saint Kitts and Nevis	0 (0 to 0)	0 (0 to 0)	1.34 (1.01 to 1.75)	1.23 (0.81 to 1.8)	-0.32 (-1.22 to 0.58)
Saint Lucia	1 (1 to 1)	2 (2 to 3)	2.94 (2.23 to 3.84)	4.41 (3 to 6.16)	1.28 (0.28 to 2.29)*
Saint Vincent and the Grenadines	1 (0 to 1)	2 (1 to 2)	2.89 (2.15 to 3.8)	5.33 (3.8 to 7.21)	1.8 (1.02 to 2.59)*
Suriname	1 (1 to 2)	3 (1 to 5)	1.4 (0.78 to 2.31)	1.94 (0.98 to 3.49)	1.1 (0.26 to 1.95)*
Trinidad and Tobago	5 (4 to 6)	12 (8 to 18)	1.74 (1.31 to 2.27)	3.17 (2.06 to 4.71)	1.9 (0.5 to 3.32)*
United States Virgin Islands	0 (0 to 1)	0 (0 to 0)	1.1 (0.57 to 1.96)	1.09 (0.46 to 2.32)	0.11 (-0.19 to 0.42)
Colombia	165 (111 to 236)	532 (327 to 821)	2.25 (1.52 to 3.2)	4 (2.46 to 6.17)	1.96 (1.64 to 2.27)*

Costa Rica	23 (15 to 33)	46 (29 to 70)	3.38 (2.24 to 4.86)	3.43 (2.16 to 5.21)	-0.34 (-1.59 to 0.94)
El Salvador	16 (11 to 23)	59 (37 to 90)	1.58 (1.07 to 2.23)	3.37 (2.12 to 5.14)	2.41 (1.27 to 3.56)*
Guatemala	19 (15 to 25)	84 (61 to 115)	1.29 (0.99 to 1.66)	2.18 (1.56 to 2.96)	1.84 (0.83 to 2.86)*
Honduras	5 (2 to 8)	19 (9 to 38)	0.54 (0.29 to 0.97)	0.79 (0.37 to 1.53)	1.33 (0.98 to 1.69)*
Mexico	242 (224 to 260)	999 (811 to 1195)	1.41 (1.3 to 1.51)	2.74 (2.23 to 3.28)	2.26 (1.9 to 2.62)*
Nicaragua	7 (4 to 11)	29 (15 to 52)	1.03 (0.58 to 1.64)	1.69 (0.89 to 2.99)	1.44 (0.91 to 1.97)*
Panama	19 (13 to 29)	33 (20 to 51)	3.5 (2.27 to 5.17)	3.06 (1.84 to 4.8)	-0.36 (-1.64 to 0.94)
Venezuela (Bolivarian Republic of)	45 (34 to 59)	190 (122 to 281)	1.12 (0.86 to 1.47)	2.5 (1.61 to 3.69)	2.83 (2.25 to 3.42)*
Brazil	359 (314 to 413)	1036 (911 to 1193)	1.07 (0.93 to 1.22)	1.62 (1.42 to 1.86)	1.34 (1.12 to 1.56)*
Paraguay	14 (7 to 22)	42 (21 to 76)	1.7 (0.95 to 2.79)	2.35 (1.16 to 4.22)	1.22 (0.45 to 2)*
Afghanistan	37 (8 to 112)	284 (87 to 615)	2.01 (0.43 to 6.02)	4.87 (1.5 to 10.44)	2.89 (2.65 to 3.14)*
Algeria	158 (80 to 292)	794 (379 to 1556)	3.28 (1.7 to 5.97)	6.66 (3.18 to 13.08)	2.36 (2.21 to 2.5)*
Bahrain	4 (2 to 8)	29 (14 to 53)	4.77 (2.37 to 8.52)	8.37 (4.14 to 15.44)	2.01 (0.62 to 3.42)*
Egypt	163 (83 to 283)	644 (328 to 1133)	1.42 (0.73 to 2.45)	2.62 (1.34 to 4.6)	1.96 (1.86 to 2.07)*
Iran (Islamic Republic of)	153 (78 to 254)	1174 (445 to 1806)	1.52 (0.76 to 2.51)	4.47 (1.73 to 6.87)	3.45 (3.1 to 3.81)*

Iraq	114 (51 to 216)	628 (305 to 1190)	3.43 (1.55 to 6.47)	6.43 (3.14 to 12.12)	2.04 (1.48 to 2.6)*
Jordan	31 (15 to 57)	161 (77 to 314)	4.82 (2.33 to 8.76)	5.6 (2.68 to 10.89)	0.57 (-0.46 to 1.62)
Kuwait	27 (17 to 40)	108 (66 to 165)	7.43 (4.67 to 11.02)	6.17 (3.79 to 9.47)	-0.93 (-5.95 to 4.35)
Lebanon	33 (15 to 62)	92 (46 to 168)	4.78 (2.22 to 8.89)	5.73 (2.86 to 10.5)	0.61 (0.34 to 0.88)*
Libya	48 (23 to 91)	247 (112 to 472)	6.7 (3.24 to 12.6)	11.73 (5.26 to 22.48)	1.97 (0.78 to 3.18)*
Morocco	101 (49 to 193)	341 (156 to 712)	1.83 (0.91 to 3.45)	3.44 (1.57 to 7.2)	2 (1.72 to 2.28)*
Oman	6 (3 to 12)	38 (18 to 73)	2.11 (0.99 to 4.04)	3.57 (1.71 to 6.82)	1.77 (0.89 to 2.67)*
Palestine	11 (5 to 22)	49 (25 to 88)	3.37 (1.61 to 6.48)	4.32 (2.2 to 7.78)	0.71 (0.39 to 1.03)*
Qatar	5 (2 to 9)	34 (15 to 73)	6.34 (3.09 to 11.97)	5.48 (2.47 to 11.64)	-0.65 (-2.07 to 0.8)
Saudi Arabia	110 (48 to 217)	1517 (732 to 2866)	4.03 (1.76 to 7.95)	13.7 (6.62 to 25.92)	4.04 (3.75 to 4.33)*
Sudan	57 (14 to 166)	303 (113 to 619)	1.48 (0.37 to 4.17)	3.05 (1.15 to 6.16)	2.33 (2.06 to 2.6)*
Syrian Arab Republic	35 (12 to 98)	146 (60 to 293)	1.63 (0.54 to 4.49)	3.77 (1.52 to 7.68)	2.77 (1.97 to 3.59)*
Tunisia	62 (30 to 115)	217 (102 to 414)	3.46 (1.68 to 6.4)	6.41 (3 to 12.34)	2 (1.79 to 2.22)*
Turkey	442 (201 to 811)	1068 (510 to 1940)	3.58 (1.65 to 6.52)	4.53 (2.15 to 8.29)	0.74 (0.42 to 1.06)*
United Arab Emirates	15 (6 to 29)	125 (61 to 236)	5.48 (2.22 to 10.55)	6.15 (2.93 to 11.6)	0.29 (-0.74 to 1.33)

Yemen	19 (6 to 46)	148 (66 to 286)	0.91 (0.31 to 2.25)	2.1 (0.95 to 4)	2.76 (2.05 to 3.47)*
Bangladesh	329 (148 to 619)	1338 (515 to 3411)	1.51 (0.7 to 2.8)	2.94 (1.14 to 7.45)	2.25 (1.91 to 2.59)*
Bhutan	2 (1 to 3)	6 (2 to 14)	1.44 (0.6 to 2.75)	2.8 (1.07 to 6.91)	2.12 (1.86 to 2.38)*
India	2725 (2002 to 3983)	11727 (8484 to 16205)	1.42 (1.05 to 2.07)	3.15 (2.28 to 4.34)	2.52 (1.99 to 3.06)*
Nepal	57 (23 to 114)	247 (103 to 517)	1.35 (0.55 to 2.66)	2.85 (1.2 to 5.87)	2.46 (2.23 to 2.7)*
Pakistan	561 (325 to 939)	3101 (1618 to 5426)	2.59 (1.52 to 4.27)	5.26 (2.76 to 9.16)	2.25 (2.01 to 2.5)*
China	4324 (2991 to 5696)	10017 (7004 to 15723)	1.53 (1.06 to 2.01)	2.65 (1.85 to 4.18)	1.83 (1.6 to 2.06)*
Democratic People's Republic of Korea	119 (55 to 226)	274 (120 to 517)	2.26 (1.04 to 4.27)	3.89 (1.7 to 7.36)	1.8 (1.66 to 1.94)*
Taiwan (Province of China)	334 (220 to 482)	658 (419 to 973)	6.2 (4.11 to 8.92)	9.6 (6.07 to 14.32)	1.38 (0.97 to 1.79)*
American Samoa	0 (0 to 0)	0 (0 to 1)	1.92 (0.98 to 3.49)	3.7 (1.87 to 6.65)	2.01 (1.77 to 2.25)*
Cook Islands	0 (0 to 0)	0 (0 to 0)	0.27 (0.12 to 0.55)	0.63 (0.27 to 1.22)	2.68 (1.59 to 3.79)*
Micronesia (Federated States of)	0 (0 to 0)	0 (0 to 1)	0.78 (0.36 to 1.46)	1.39 (0.65 to 2.74)	1.85 (1.77 to 1.94)*
Fiji	5 (2 to 9)	8 (4 to 16)	2.62 (1.31 to 4.82)	3.55 (1.64 to 7.02)	0.99 (0.37 to 1.62)*
Guam	0 (0 to 0)	1 (0 to 1)	0.48 (0.28 to 1.05)	1.43 (0.93 to 2.06)	3.66 (1.42 to 5.96)*

Kiribati	0 (0 to 0)	0 (0 to 0)	0.02 (0.01 to 0.05)	0.02 (0.01 to 0.07)	0.96 (0.76 to 1.17)*
Marshall Islands	0 (0 to 0)	0 (0 to 0)	0.67 (0.33 to 1.19)	1.35 (0.59 to 2.61)	2.27 (2.12 to 2.42)*
Nauru	0 (0 to 0)	0 (0 to 0)	1.12 (0.45 to 2.2)	2.04 (0.73 to 4.12)	1.93 (1.83 to 2.03)*
Niue	0 (0 to 0)	0 (0 to 0)	0.85 (0.4 to 1.6)	2.07 (1.04 to 3.97)	3 (2.58 to 3.41)*
Northern Mariana Islands	0 (0 to 0)	0 (0 to 0)	0.88 (0.41 to 1.66)	1.27 (0.65 to 2.34)	0.85 (0.24 to 1.45)*
Palau	0 (0 to 0)	0 (0 to 0)	1.1 (0.52 to 2.06)	1.72 (0.78 to 3.28)	1.41 (1.32 to 1.5)*
Papua New Guinea	5 (2 to 10)	19 (8 to 46)	0.59 (0.24 to 1.25)	0.78 (0.31 to 1.87)	0.9 (0.67 to 1.12)*
Samoa	1 (0 to 2)	2 (1 to 5)	2.7 (1.24 to 5.15)	5.5 (2.33 to 11.05)	2.34 (2.17 to 2.51)*
Solomon Islands	0 (0 to 1)	2 (1 to 3)	0.47 (0.15 to 1.04)	1.05 (0.48 to 2.11)	2.57 (2.32 to 2.83)*
Tokelau	0 (0 to 0)	0 (0 to 0)	0.85 (0.39 to 1.56)	2.22 (1.12 to 4.27)	3.3 (2.75 to 3.86)*
Tonga	0 (0 to 0)	0 (0 to 0)	0.66 (0.32 to 1.23)	1.06 (0.48 to 2.12)	1.5 (1.25 to 1.74)*
Tuvalu	0 (0 to 0)	0 (0 to 0)	0.72 (0.35 to 1.32)	1.36 (0.65 to 2.62)	2.08 (1.9 to 2.26)*
Vanuatu	0 (0 to 0)	1 (0 to 1)	0.54 (0.23 to 1.09)	0.94 (0.46 to 1.77)	1.71 (1.49 to 1.93)*
Cambodia	37 (16 to 70)	177 (84 to 345)	1.78 (0.79 to 3.28)	4.13 (1.96 to 8.03)	2.79 (2.7 to 2.87)*
Indonesia	771 (453 to 1078)	2332 (1391 to 3575)	1.88 (1.11 to 2.62)	2.92 (1.74 to 4.48)	1.45 (1.25 to 1.66)*

Lao People's Democratic Republic	14 (5 to 27)	56 (26 to 110)	1.76 (0.67 to 3.4)	3.06 (1.41 to 5.94)	1.79 (1.63 to 1.95)*
Malaysia	117 (57 to 222)	463 (229 to 861)	2.9 (1.45 to 5.41)	5.57 (2.77 to 10.31)	2.11 (1.64 to 2.58)*
Maldives	0 (0 to 1)	2 (1 to 3)	1.2 (0.36 to 2.38)	1.38 (0.66 to 2.73)	0.31 (0.15 to 0.48)*
Mauritius	5 (3 to 6)	7 (5 to 9)	1.72 (1.27 to 2.27)	1.88 (1.36 to 2.49)	-0.42 (-5.23 to 4.63)
Myanmar	195 (82 to 382)	466 (224 to 872)	2.22 (0.96 to 4.27)	3.08 (1.48 to 5.77)	1.06 (0.92 to 1.2)*
Philippines	471 (311 to 674)	1415 (923 to 2149)	3.6 (2.38 to 5.13)	5.17 (3.38 to 7.82)	1.15 (0.82 to 1.49)*
Seychelles	0 (0 to 0)	0 (0 to 1)	1.06 (0.55 to 1.82)	1.46 (0.72 to 2.54)	1.3 (0.99 to 1.6)*
Sri Lanka	97 (50 to 171)	239 (104 to 468)	2.31 (1.19 to 4.04)	4.03 (1.75 to 7.89)	1.87 (1.12 to 2.62)*
Thailand	389 (205 to 668)	1025 (500 to 1915)	2.72 (1.45 to 4.64)	5.3 (2.54 to 9.95)	2.19 (1.53 to 2.85)*
Timor-Leste	2 (1 to 3)	6 (3 to 11)	1.12 (0.51 to 2.19)	2.05 (0.98 to 3.89)	1.95 (1.48 to 2.41)*
Viet Nam	434 (198 to 846)	2734 (1226 to 5279)	2.94 (1.39 to 5.6)	9.83 (4.36 to 19.09)	3.98 (3.79 to 4.16)*
Angola	8 (3 to 15)	39 (15 to 84)	0.4 (0.17 to 0.78)	0.6 (0.23 to 1.29)	1.37 (0.68 to 2.06)*
Central African Republic	2 (1 to 4)	5 (2 to 10)	0.45 (0.21 to 0.84)	0.43 (0.18 to 0.87)	-0.28 (-0.37 to -0.19)*
Congo	3 (1 to 5)	13 (5 to 27)	0.6 (0.27 to 1.13)	0.96 (0.38 to 1.99)	1.69 (0.99 to 2.41)*

Democratic Republic of the Congo	27 (11 to 55)	84 (31 to 190)	0.39 (0.16 to 0.79)	0.48 (0.18 to 1.08)	0.68 (0.43 to 0.93)*
Equatorial Guinea	0 (0 to 1)	3 (1 to 7)	0.4 (0.18 to 0.78)	0.94 (0.32 to 2.11)	2.86 (1.99 to 3.74)*
Gabon	1 (1 to 2)	4 (2 to 8)	0.67 (0.31 to 1.26)	0.93 (0.37 to 1.96)	1.1 (0.84 to 1.37)*
Burundi	23 (10 to 45)	56 (24 to 120)	2 (0.89 to 3.88)	1.98 (0.85 to 4.2)	-0.06 (-0.26 to 0.15)
Comoros	2 (1 to 4)	5 (2 to 12)	1.89 (0.79 to 4.06)	2.77 (1.07 to 6.39)	1.16 (0.48 to 1.84)*
Djibouti	1 (0 to 2)	7 (2 to 17)	1.33 (0.53 to 2.9)	2.14 (0.75 to 5.4)	1.63 (1.47 to 1.8)*
Eritrea	10 (5 to 19)	37 (15 to 76)	1.55 (0.75 to 2.83)	2.41 (1.02 to 4.98)	1.41 (1.21 to 1.61)*
Ethiopia	448 (236 to 762)	1371 (708 to 2909)	4.47 (2.38 to 7.57)	5.45 (2.84 to 11.58)	0.62 (0.44 to 0.79)*
Kenya	25 (15 to 44)	122 (69 to 232)	0.56 (0.33 to 1)	1.03 (0.59 to 1.93)	1.87 (1.31 to 2.43)*
Madagascar	44 (21 to 81)	162 (70 to 320)	1.82 (0.89 to 3.31)	2.43 (1.07 to 4.8)	0.92 (0.81 to 1.03)*
Malawi	36 (17 to 68)	133 (55 to 291)	1.77 (0.85 to 3.28)	2.99 (1.27 to 6.35)	1.76 (1.55 to 1.98)*
Mozambique	41 (17 to 95)	155 (60 to 390)	1.43 (0.61 to 3.25)	2.31 (0.9 to 5.78)	1.54 (1.28 to 1.79)*
Rwanda	38 (18 to 70)	89 (37 to 197)	2.62 (1.24 to 4.8)	2.71 (1.13 to 5.93)	0.09 (-0.08 to 0.25)
Somalia	21 (9 to 42)	68 (27 to 139)	1.38 (0.58 to 2.7)	1.59 (0.63 to 3.26)	0.48 (0.33 to 0.62)*
South Sudan	15 (6 to 32)	48 (20 to 104)	1.32 (0.55 to 2.77)	2.27 (0.92 to 4.91)	1.83 (1.54 to 2.12)*

United Republic of Tanzania	95 (43 to 186)	350 (147 to 725)	1.83 (0.85 to 3.52)	2.59 (1.11 to 5.28)	1.11 (0.94 to 1.29)*
Uganda	47 (21 to 89)	376 (166 to 758)	1.41 (0.66 to 2.67)	4.01 (1.82 to 7.93)	3.27 (3 to 3.54)*
Zambia	33 (16 to 60)	161 (51 to 492)	2.11 (1.03 to 3.77)	3.63 (1.2 to 10.76)	1.75 (1.59 to 1.91)*
Botswana	2 (1 to 4)	6 (2 to 14)	0.75 (0.3 to 1.7)	0.87 (0.31 to 2.09)	0.46 (-0.87 to 1.81)
Eswatini	1 (1 to 2)	4 (1 to 8)	0.84 (0.36 to 1.65)	1.38 (0.51 to 3.02)	1.54 (1.08 to 1.99)*
Lesotho	1 (1 to 3)	4 (2 to 9)	0.45 (0.17 to 1.03)	1.07 (0.44 to 2.15)	2.83 (2.14 to 3.52)*
Namibia	2 (1 to 4)	9 (4 to 19)	0.78 (0.37 to 1.48)	1.52 (0.65 to 3.07)	2.25 (1.88 to 2.62)*
South Africa	110 (83 to 159)	208 (154 to 324)	1.35 (1.02 to 1.92)	1.34 (0.99 to 2.07)	-0.05 (-1.26 to 1.17)
Zimbabwe	41 (20 to 76)	161 (69 to 326)	2.15 (1.09 to 3.97)	4.46 (1.95 to 8.97)	2.39 (1.19 to 3.6)*
Benin	3 (1 to 6)	10 (4 to 21)	0.3 (0.14 to 0.63)	0.35 (0.15 to 0.76)	0.45 (0.27 to 0.63)*
Burkina Faso	8 (3 to 16)	19 (8 to 40)	0.42 (0.18 to 0.86)	0.42 (0.18 to 0.84)	0 (-0.2 to 0.2)
Cabo Verde	0 (0 to 0)	1 (0 to 2)	0.17 (0.07 to 0.34)	0.77 (0.18 to 1.63)	5.05 (4.53 to 5.57)*
Cameroon	10 (5 to 20)	38 (16 to 76)	0.52 (0.23 to 1.01)	0.57 (0.24 to 1.11)	0.22 (-0.09 to 0.54)
Chad	3 (1 to 6)	10 (5 to 20)	0.27 (0.12 to 0.57)	0.33 (0.15 to 0.64)	0.67 (0.51 to 0.83)*
Coted'Ivoire	23 (11 to 44)	102 (46 to 201)	0.96 (0.47 to 1.81)	1.64 (0.75 to 3.18)	1.74 (1.33 to 2.15)*

Gambia	1 (0 to 2)	4 (2 to 9)	0.46 (0.21 to 0.87)	0.78 (0.33 to 1.55)	1.72 (0.56 to 2.89)*
Ghana	1 (0 to 2)	5 (2 to 10)	0.03 (0.02 to 0.06)	0.06 (0.02 to 0.12)	1.72 (1.56 to 1.88)*
Guinea	7 (3 to 12)	22 (9 to 47)	0.52 (0.25 to 0.96)	0.73 (0.31 to 1.54)	1.06 (0.86 to 1.25)*
Guinea-Bissau	1 (0 to 2)	3 (1 to 5)	0.52 (0.23 to 1.01)	0.56 (0.26 to 1.06)	0.22 (0.06 to 0.38)*
Liberia	2 (1 to 3)	7 (3 to 14)	0.34 (0.14 to 0.68)	0.55 (0.24 to 1.08)	1.64 (1.3 to 1.99)*
Mali	27 (14 to 49)	88 (37 to 182)	1.59 (0.82 to 2.82)	1.87 (0.81 to 3.83)	0.53 (0.32 to 0.73)*
Mauritania	1 (1 to 3)	4 (2 to 9)	0.34 (0.14 to 0.66)	0.5 (0.21 to 1.04)	1.24 (0.96 to 1.52)*
Niger	4 (2 to 9)	11 (5 to 23)	0.28 (0.12 to 0.59)	0.27 (0.12 to 0.56)	-0.18 (-0.59 to 0.24)
Nigeria	14 (8 to 26)	69 (35 to 133)	0.09 (0.05 to 0.16)	0.14 (0.07 to 0.27)	1.47 (1.17 to 1.77)*
Sao Tome and Principe	0 (0 to 0)	0 (0 to 0)	0.04 (0.02 to 0.08)	0.07 (0.03 to 0.17)	2.05 (1.41 to 2.69)*
Senegal	6 (2 to 12)	15 (6 to 33)	0.38 (0.16 to 0.81)	0.43 (0.18 to 0.97)	0.25 (-0.39 to 0.89)
Sierra Leone	2 (1 to 5)	8 (3 to 17)	0.29 (0.12 to 0.62)	0.43 (0.18 to 0.9)	1.3 (0.9 to 1.71)*
Togo	3 (1 to 6)	10 (4 to 20)	0.44 (0.19 to 0.89)	0.48 (0.2 to 1.01)	0.31 (0.09 to 0.54)*

Table S2: Prevalence of WCBA thyroid cancer in 1990 and 2021, and its ASR estimated AAPC from 1990 to 2021.

Location	Number, 95% UI		Age-standardized prevalence rate (per 100 000), 95% UI		AAPC of prevalence rate, No. (95% CI)
	1990	2021	1990	2021	
Global	235219 (207596 to 271552)	611351 (506694 to 754720)	19.47 (17.25 to 22.38)	30.46 (25.21 to 37.68)	1.44 (1.37 to 1.52)*
**SDI regions**					
Low SDI	13039 (9174 to 18287)	53408 (37555 to 84058)	12.93 (9.15 to 18.14)	21.44 (15.12 to 33.52)	1.6 (1.34 to 1.85)*
Low-middle SDI	30787 (23610 to 42371)	122385 (90137 to 173681)	12.47 (9.63 to 17.04)	25.01 (18.49 to 35.24)	2.23 (1.99 to 2.47)*
Middle SDI	56989 (45921 to 71184)	202409 (157807 to 252974)	14.69 (11.9 to 18.34)	30.68 (23.88 to 38.42)	2.4 (2.29 to 2.52)*
High-middle SDI	58300 (49814 to 66901)	110248 (90157 to 139698)	22.57 (19.34 to 25.84)	30.47 (24.84 to 38.82)	0.95 (0.67 to 1.23)*
High SDI	75817 (69714 to 82180)	122438 (110262 to 139877)	32.09 (29.51 to 34.79)	43.39 (38.98 to 49.78)	1.03 (0.81 to 1.25)*
**GBD super regions**					
Central Europe, Eastern Europe, and Central Asia	27057 (25022 to 29420)	32578 (29093 to 36531)	26.25 (24.29 to 28.56)	27.58 (24.61 to 30.95)	-0.09 (-0.9 to 0.72)
High-income	75543 (69394 to 82257)	103952 (95556 to 113902)	30.98 (28.45 to 33.74)	37.14 (34.13 to 40.72)	0.59 (0.22 to 0.96)*
Latin America and Caribbean	10557 (9681 to 11583)	36107 (31451 to 41663)	12.44 (11.43 to 13.61)	21.86 (19.04 to 25.23)	1.89 (1.59 to 2.2)*

North Africa and Middle East	14778 (10605 to 21827)	74345 (54887 to 98935)	22.4 (16.14 to 33.08)	46.1 (34.07 to 61.38)	2.36 (2.21 to 2.51)*
South Asia	31895 (23827 to 44963)	147029 (106471 to 208758)	13.27 (9.96 to 18.65)	30.27 (21.95 to 42.78)	2.66 (2.24 to 3.08)*
Southeast Asia, East Asia, and Oceania	65239 (47583 to 79929)	180993 (137885 to 243919)	16.36 (11.98 to 20.02)	30.86 (23.46 to 41.72)	2.08 (1.93 to 2.23)*
Sub-Saharan Africa	10149 (7315 to 13835)	36348 (24601 to 60702)	10.36 (7.53 to 14.06)	14.29 (9.74 to 23.65)	1.03 (0.9 to 1.16)*
**GBD regions**					
Central Asia	2565 (2152 to 3067)	4396 (3575 to 5364)	18.57 (15.57 to 22.25)	17.44 (14.18 to 21.27)	-0.54 (-1.52 to 0.45)
Central Europe	10251 (8760 to 11806)	8597 (7083 to 10129)	31.63 (27.05 to 36.42)	26.46 (21.77 to 31.23)	-0.46 (-1.19 to 0.27)
Eastern Europe	14241 (12934 to 15925)	19585 (16716 to 23023)	25.09 (22.78 to 28.07)	32.55 (27.75 to 38.35)	0.43 (-0.82 to 1.69)
Australasia	1364 (951 to 1914)	2906 (1941 to 4212)	24.83 (17.32 to 34.84)	35.42 (23.59 to 51.45)	1.27 (0.06 to 2.49)*
High-income Asia Pacific	16404 (12906 to 20703)	24643 (19126 to 32147)	33.46 (26.28 to 42.35)	51.1 (39.58 to 67.26)	1.53 (0.98 to 2.09)*
High-income North America	22643 (20918 to 24598)	38870 (35728 to 42288)	29.27 (27.05 to 31.8)	42.06 (38.65 to 45.77)	1.16 (0.63 to 1.7)*
Southern Latin America	2233 (1675 to 2928)	4148 (3073 to 5521)	18.7 (14.05 to 24.51)	22.39 (16.56 to 29.83)	0.74 (-0.24 to 1.73)
Western Europe	32900 (28540 to 37993)	33384 (28499 to 39211)	32.89 (28.53 to 38)	29.61 (25.21 to 34.9)	-0.32 (-0.83 to 0.2)
Andean Latin America	1188 (826 to 1688)	5816 (3786 to 8755)	14.94 (10.47 to 21.12)	33.57 (21.88 to 50.48)	2.84 (1.91 to 3.79)*
Caribbean	1263 (1004 to 1586)	2584 (1946 to 3386)	15.39 (12.29 to 19.28)	21.01 (15.82 to 27.55)	1.01 (0.45 to 1.57)*

Central Latin America	4796 (4228 to 5479)	17963 (14855 to 21467)	14.08 (12.45 to 16.03)	25.8 (21.34 to 30.84)	1.94 (1.67 to 2.21)*
Tropical Latin America	3311 (2901 to 3808)	9744 (8588 to 11191)	9.59 (8.41 to 11.01)	14.81 (13.04 to 17.01)	1.4 (1.18 to 1.61)*
East Asia	42633 (30436 to 55021)	99860 (71944 to 152974)	14.52 (10.35 to 18.67)	25.53 (18.36 to 39.29)	1.88 (1.66 to 2.09)*
Oceania	108 (60 to 175)	321 (168 to 590)	8.23 (4.56 to 13.18)	9.77 (5.12 to 17.94)	0.54 (0.37 to 0.71)*
Southeast Asia	22497 (15392 to 29102)	80811 (56217 to 109927)	21.64 (15.02 to 27.77)	42.28 (29.36 to 57.61)	2.17 (1.92 to 2.42)*
Central Sub-Saharan Africa	344 (178 to 647)	1300 (607 to 2609)	3.43 (1.8 to 6.37)	4.77 (2.24 to 9.47)	1.02 (0.85 to 1.18)*
Eastern Sub-Saharan Africa	7427 (4995 to 10611)	27875 (17805 to 50176)	19.47 (13.18 to 27.82)	28.6 (18.38 to 51.3)	1.24 (1.18 to 1.31)*
Southern Sub-Saharan Africa	1379 (1016 to 1853)	3401 (2341 to 4954)	12.42 (9.15 to 16.57)	16.12 (11.15 to 23.37)	0.84 (0.11 to 1.58)*
Western Sub-Saharan Africa	999 (671 to 1445)	3772 (2459 to 5890)	2.71 (1.84 to 3.85)	3.6 (2.37 to 5.57)	0.92 (0.81 to 1.03)*
**Countries and territories**					
Armenia	130 (83 to 194)	224 (142 to 332)	16.48 (10.59 to 24.38)	26.64 (16.92 to 39.46)	1.37 (0.39 to 2.35)*
Azerbaijan	151 (80 to 265)	310 (155 to 571)	9.99 (5.41 to 17.19)	10.61 (5.33 to 19.47)	0.3 (-0.43 to 1.03)
Georgia	264 (166 to 402)	375 (240 to 563)	19.71 (12.45 to 29.87)	40.44 (25.79 to 60.95)	2.09 (0.23 to 3.99)*
Kazakhstan	1405 (1058 to 1835)	1830 (1300 to 2493)	37.84 (28.47 to 49.52)	35.22 (25.02 to 47.98)	-0.69 (-2.83 to 1.49)
Kyrgyzstan	321 (204 to 486)	503 (307 to 767)	34.52 (22.33 to 51.8)	30.05 (18.42 to 45.7)	-0.82 (-2.49 to 0.87)

Mongolia	47 (23 to 85)	174 (92 to 302)	12.54 (6.3 to 22.13)	19.03 (10.11 to 33.12)	1.36 (0.42 to 2.31)*
Tajikistan	2 (1 to 3)	4 (2 to 6)	0.21 (0.1 to 0.39)	0.15 (0.08 to 0.27)	-1.07 (-1.55 to -0.6)*
Turkmenistan	113 (86 to 147)	240 (160 to 351)	16.11 (12.31 to 20.86)	19.38 (12.93 to 28.34)	0.7 (-2.09 to 3.57)
Uzbekistan	131 (83 to 200)	736 (459 to 1116)	3.46 (2.23 to 5.25)	8.01 (5 to 12.14)	2.66 (1.96 to 3.37)*
Albania	91 (50 to 154)	115 (55 to 214)	12.79 (7.07 to 21.56)	17.37 (8.31 to 32.51)	0.82 (-0.05 to 1.69)
Bosnia and Herzegovina	162 (106 to 235)	139 (83 to 224)	14.35 (9.38 to 20.84)	15.67 (9.35 to 25.31)	0.11 (-0.55 to 0.78)
Bulgaria	349 (236 to 503)	304 (195 to 460)	15.1 (10.18 to 21.83)	15.77 (10.02 to 23.92)	0.17 (-1.53 to 1.9)
Croatia	496 (326 to 725)	313 (195 to 465)	38.38 (25.22 to 56.13)	28.37 (17.62 to 42.4)	-1.19 (-2.49 to 0.13)
Czechia	1322 (890 to 1909)	1191 (749 to 1798)	45.9 (30.8 to 66.47)	38.61 (24.06 to 58.57)	-0.57 (-1.62 to 0.49)
Hungary	1078 (715 to 1543)	740 (492 to 1061)	37.41 (24.71 to 53.7)	26.03 (17.06 to 37.73)	-0.99 (-2.31 to 0.36)
Montenegro	51 (33 to 77)	53 (34 to 81)	33.28 (21.56 to 50.37)	31.59 (20.25 to 48.71)	-0.03 (-0.57 to 0.52)
North Macedonia	89 (58 to 127)	108 (65 to 174)	17.52 (11.48 to 24.9)	16.94 (10.3 to 27.49)	0.03 (-0.79 to 0.85)
Poland	4523 (3474 to 5793)	3688 (2722 to 4892)	45.29 (34.81 to 58.07)	33.47 (24.7 to 44.44)	-1.1 (-2.71 to 0.54)
Romania	1098 (729 to 1585)	948 (600 to 1421)	19.35 (12.82 to 27.96)	18.14 (11.39 to 27.4)	0 (-1.2 to 1.22)
Serbia	336 (179 to 614)	405 (211 to 718)	13.74 (7.32 to 25.13)	16.69 (8.6 to 29.87)	0.59 (-0.03 to 1.2)

Slovakia	370 (206 to 613)	405 (210 to 724)	26.85 (14.97 to 44.45)	25.72 (13.11 to 46.32)	-0.13 (-0.85 to 0.6)
Slovenia	122 (82 to 175)	63 (38 to 96)	23.33 (15.67 to 33.5)	11.09 (6.73 to 17.02)	-2.46 (-3.15 to -1.77)*
Belarus	894 (567 to 1344)	834 (494 to 1290)	34.68 (22.02 to 51.94)	30.91 (18.24 to 48.02)	-0.84 (-5.31 to 3.84)
Estonia	168 (108 to 252)	92 (59 to 138)	41.58 (26.72 to 62.39)	27.27 (17.37 to 40.82)	-1.45 (-3.31 to 0.44)
Latvia	228 (147 to 337)	124 (78 to 185)	32.94 (21.22 to 48.87)	24.93 (15.54 to 37.31)	-0.97 (-2.72 to 0.81)
Lithuania	441 (287 to 648)	196 (126 to 290)	46.34 (30.08 to 68.12)	27.45 (17.59 to 40.68)	-1.92 (-3.24 to -0.58)*
Republic of Moldova	139 (104 to 182)	155 (112 to 210)	12.41 (9.34 to 16.31)	14.34 (10.35 to 19.41)	-0.01 (-0.79 to 0.78)
Russian Federation	9504 (8921 to 10194)	14552 (12580 to 16595)	25.31 (23.77 to 27.14)	34.37 (29.73 to 39.21)	0.34 (-1.25 to 1.95)
Ukraine	2867 (1833 to 4296)	3631 (1783 to 6439)	21.54 (13.76 to 32.31)	29.13 (14.31 to 51.79)	1.04 (0.35 to 1.75)*
Australia	1235 (826 to 1784)	2736 (1777 to 4025)	26.92 (18.02 to 38.9)	39.72 (25.72 to 58.58)	1.38 (0.1 to 2.68)*
New Zealand	129 (87 to 188)	170 (114 to 245)	14.44 (9.69 to 20.98)	12.89 (8.61 to 18.55)	-0.41 (-2.82 to 2.05)
Brunei Darussalam	19 (9 to 35)	55 (29 to 94)	33.38 (15.58 to 61.7)	40.99 (21.71 to 70.51)	0.63 (0.24 to 1.02)*
Japan	12962 (9983 to 16568)	14924 (11485 to 18198)	35.18 (27.1 to 45)	46.74 (36.32 to 56.37)	0.99 (0.67 to 1.32)*
Singapore	291 (192 to 423)	469 (300 to 700)	31.7 (20.93 to 45.91)	26.14 (16.65 to 39.02)	-0.36 (-0.95 to 0.23)
Republic of Korea	3132 (1681 to 5891)	9195 (4809 to 16029)	27.31 (14.78 to 51.2)	64.04 (33.1 to 112.69)	2.79 (1.93 to 3.67)*

Canada	2772 (1840 to 4011)	2905 (1862 to 4329)	35.82 (23.82 to 51.78)	30.74 (19.63 to 45.9)	-0.34 (-1.27 to 0.6)
Greenland	3 (2 to 6)	2 (1 to 5)	23.69 (11.17 to 42.61)	17.94 (8.35 to 36.84)	-0.85 (-1.3 to -0.41)*
United States of America	19867 (18484 to 21385)	35962 (33021 to 39164)	28.55 (26.57 to 30.73)	43.38 (39.84 to 47.26)	1.37 (0.38 to 2.38)*
Argentina	1399 (927 to 2031)	2435 (1545 to 3669)	17.71 (11.73 to 25.71)	19.4 (12.29 to 29.27)	0.48 (-0.61 to 1.59)
Chile	663 (431 to 980)	1437 (927 to 2155)	20.32 (13.24 to 29.89)	28.27 (18.19 to 42.48)	1.2 (0.31 to 2.1)*
Uruguay	170 (110 to 257)	276 (175 to 413)	22.54 (14.64 to 34.04)	30.55 (19.33 to 45.81)	1.03 (0.43 to 1.65)*
Andorra	3 (1 to 6)	7 (3 to 12)	22.1 (10.02 to 41.5)	24.21 (10.83 to 45.35)	0.31 (-0.06 to 0.69)
Austria	933 (623 to 1355)	787 (510 to 1172)	45 (30.01 to 65.39)	33.2 (21.45 to 49.59)	-0.95 (-1.53 to -0.36)*
Belgium	613 (404 to 894)	674 (435 to 1002)	23.98 (15.8 to 34.98)	23.15 (14.89 to 34.52)	-0.35 (-1.65 to 0.97)
Cyprus	45 (22 to 83)	70 (33 to 145)	22.12 (10.7 to 40.93)	15.8 (7.29 to 32.91)	-0.91 (-1.69 to -0.13)*
Denmark	225 (153 to 321)	122 (78 to 181)	15.57 (10.6 to 22.29)	8.34 (5.33 to 12.46)	-2.09 (-3.51 to -0.66)*
Finland	453 (307 to 654)	253 (158 to 376)	31.26 (21.12 to 45.2)	19.39 (12.05 to 28.9)	-1.38 (-2.85 to 0.11)
France	6028 (4057 to 8587)	7877 (4944 to 11719)	40.33 (27.1 to 57.45)	48.03 (29.96 to 71.9)	0.69 (0.05 to 1.32)*
Germany	8830 (6026 to 12614)	7258 (4669 to 10663)	42.57 (29.05 to 60.86)	36.1 (23.12 to 53.17)	-0.74 (-1.15 to -0.34)*
Greece	433 (320 to 573)	482 (353 to 635)	16.42 (12.12 to 21.71)	16.94 (12.42 to 22.31)	0.06 (-0.81 to 0.95)

Iceland	46 (30 to 66)	39 (24 to 57)	73.33 (48.37 to 106.02)	43.54 (27.58 to 64.9)	-1.3 (-3.12 to 0.56)
Ireland	151 (99 to 220)	292 (187 to 436)	17.68 (11.61 to 25.74)	20.41 (12.97 to 30.59)	0.59 (-0.25 to 1.44)
Israel	245 (161 to 362)	472 (308 to 706)	21.13 (13.94 to 31.12)	19.91 (12.95 to 29.86)	0.06 (-2.45 to 2.63)
Italy	8573 (6479 to 11279)	7621 (5675 to 9859)	56.93 (43.02 to 74.88)	46.38 (34.5 to 60.05)	-0.42 (-1.22 to 0.39)
Luxembourg	26 (19 to 34)	35 (25 to 46)	24.46 (18.23 to 32.27)	18.67 (13.6 to 24.81)	-0.92 (-1.76 to -0.08)*
Malta	23 (15 to 34)	33 (21 to 50)	21.65 (14.05 to 31.89)	28 (17.83 to 42.62)	0.89 (-0.71 to 2.52)
Monaco	2 (1 to 4)	3 (1 to 6)	23.73 (11.72 to 43.61)	36.5 (16.61 to 68.85)	1.42 (1.23 to 1.61)*
Netherlands	680 (450 to 980)	794 (499 to 1170)	16.47 (10.91 to 23.74)	19.31 (12.09 to 28.61)	0.42 (0.18 to 0.65)*
Norway	177 (134 to 233)	121 (88 to 161)	15.97 (12.06 to 21)	8.71 (6.32 to 11.63)	-1.97 (-3.83 to -0.07)*
Portugal	708 (469 to 1022)	981 (641 to 1460)	27.34 (18.09 to 39.5)	32.01 (20.74 to 47.98)	0.37 (-0.7 to 1.46)
San Marino	2 (1 to 3)	3 (1 to 5)	30.15 (15.07 to 52.57)	27.67 (10.98 to 53.39)	-0.4 (-0.66 to -0.14)*
Spain	1905 (1269 to 2781)	1899 (1227 to 2831)	19.91 (13.27 to 29.09)	14.67 (9.38 to 22.03)	-1.24 (-1.55 to -0.92)*
Sweden	281 (203 to 381)	250 (169 to 358)	11.74 (8.44 to 16.01)	9.94 (6.68 to 14.26)	-0.66 (-2 to 0.69)
Switzerland	444 (293 to 638)	248 (158 to 369)	22.81 (15.02 to 32.82)	10.21 (6.49 to 15.23)	-2.62 (-4.22 to -1)*
United Kingdom	2049 (1872 to 2249)	3034 (2747 to 3350)	13.79 (12.6 to 15.14)	17.29 (15.65 to 19.1)	0.73 (0.18 to 1.27)*

Bolivia (Plurinational State of)	251 (111 to 453)	897 (425 to 1671)	19.11 (8.59 to 34.29)	30.15 (14.36 to 55.97)	1.51 (1.38 to 1.65)*
Ecuador	273 (182 to 397)	1624 (956 to 2591)	12.89 (8.68 to 18.65)	35.25 (20.76 to 56.18)	3.19 (1.66 to 4.75)*
Peru	665 (373 to 1092)	3295 (1632 to 5859)	14.67 (8.32 to 23.96)	33.8 (16.76 to 60.07)	3.05 (2.06 to 4.05)*
Antigua and Barbuda	3 (2 to 4)	8 (6 to 11)	21.11 (15.59 to 28)	30.15 (22.23 to 40.21)	1.45 (0.14 to 2.77)*
Bahamas	13 (10 to 18)	40 (26 to 57)	21.36 (15.88 to 28.11)	35.09 (23.32 to 50.2)	1.56 (0.86 to 2.26)*
Barbados	12 (9 to 16)	23 (15 to 33)	19.07 (14.14 to 24.99)	28.22 (18.76 to 40.86)	1.23 (0.46 to 2)*
Belize	2 (2 to 3)	15 (11 to 21)	6.87 (5.11 to 9.04)	13.92 (10.15 to 18.76)	1.93 (1.12 to 2.74)*
Bermuda	4 (3 to 6)	7 (4 to 10)	23.57 (14.81 to 35.45)	38.96 (23.27 to 61.13)	1.36 (0.72 to 2.01)*
Cuba	569 (371 to 832)	867 (549 to 1326)	20.17 (13.19 to 29.4)	30.06 (18.92 to 46.17)	1.29 (0.4 to 2.19)*
Dominica	1 (1 to 2)	2 (1 to 4)	8.97 (5.15 to 14.67)	12.1 (6.07 to 21.45)	0.94 (0.62 to 1.27)*
Dominican Republic	150 (84 to 258)	478 (236 to 880)	9.81 (5.53 to 16.7)	16.99 (8.42 to 31.18)	1.76 (1.12 to 2.4)*
Grenada	4 (3 to 6)	8 (5 to 12)	24.3 (15.54 to 36.65)	31.48 (19.33 to 48.65)	0.65 (-0.42 to 1.72)
Guyana	12 (7 to 18)	30 (17 to 51)	7.28 (4.59 to 10.92)	15.66 (8.77 to 26.1)	2.49 (2.21 to 2.76)*
Haiti	141 (60 to 270)	415 (184 to 801)	10.65 (4.67 to 20.23)	12.36 (5.54 to 23.78)	0.6 (0.13 to 1.07)*
Jamaica	62 (41 to 92)	228 (128 to 376)	13.33 (8.77 to 19.6)	29.93 (16.84 to 49.19)	2.69 (1.81 to 3.58)*

Puerto Rico	175 (115 to 260)	200 (124 to 310)	18.35 (12.01 to 27.22)	23.49 (14.5 to 36.49)	0.19 (-0.68 to 1.07)
Saint Kitts and Nevis	1 (1 to 1)	2 (1 to 3)	11.79 (8.84 to 15.36)	11.13 (7.3 to 16.18)	-0.24 (-1.13 to 0.66)
Saint Lucia	7 (5 to 9)	20 (14 to 29)	26.11 (19.77 to 34.04)	39.84 (27.08 to 55.64)	1.34 (0.34 to 2.34)*
Saint Vincent and the Grenadines	5 (4 to 7)	14 (10 to 19)	25.65 (19.06 to 33.8)	47.79 (34.05 to 64.69)	1.83 (1.05 to 2.62)*
Suriname	10 (6 to 17)	26 (13 to 47)	12.36 (6.89 to 20.4)	17.35 (8.78 to 31.29)	1.15 (0.32 to 1.98)*
Trinidad and Tobago	43 (32 to 56)	110 (71 to 164)	15.46 (11.65 to 20.19)	28.61 (18.53 to 42.42)	1.96 (0.55 to 3.38)*
United States Virgin Islands	3 (2 to 5)	2 (1 to 4)	9.92 (5.14 to 17.6)	9.9 (4.16 to 21.07)	0.14 (-0.16 to 0.45)
Colombia	1473 (987 to 2103)	4822 (2958 to 7427)	19.99 (13.52 to 28.43)	36.28 (22.26 to 55.86)	2.03 (1.72 to 2.34)*
Costa Rica	205 (135 to 297)	421 (265 to 639)	30.48 (20.13 to 43.91)	31.15 (19.62 to 47.31)	-0.32 (-1.58 to 0.96)
El Salvador	142 (97 to 202)	529 (332 to 809)	13.75 (9.37 to 19.46)	30.38 (19.09 to 46.42)	2.52 (1.38 to 3.66)*
Guatemala	163 (126 to 211)	752 (540 to 1022)	10.91 (8.4 to 14.11)	19.38 (13.91 to 26.32)	2 (0.97 to 3.04)*
Honduras	39 (21 to 70)	172 (78 to 335)	4.64 (2.49 to 8.32)	7.02 (3.25 to 13.5)	1.42 (1.07 to 1.78)*
Mexico	2139 (1983 to 2301)	8991 (7294 to 10747)	12.39 (11.5 to 13.33)	24.68 (20.03 to 29.49)	2.33 (1.97 to 2.69)*
Nicaragua	63 (35 to 102)	264 (138 to 468)	9.1 (5.14 to 14.54)	15.26 (8.01 to 26.97)	1.5 (0.98 to 2.04)*
Panama	174 (112 to 258)	296 (178 to 465)	31.36 (20.4 to 46.33)	27.68 (16.66 to 43.47)	-0.32 (-1.6 to 0.97)

Venezuela (Bolivarian Republic of)	399 (303 to 520)	1714 (1100 to 2526)	9.97 (7.58 to 13.02)	22.57 (14.5 to 33.26)	2.89 (2.3 to 3.48)*
Brazil	3189 (2785 to 3675)	9363 (8230 to 10767)	9.45 (8.27 to 10.87)	14.63 (12.85 to 16.83)	1.4 (1.18 to 1.63)*
Paraguay	121 (67 to 200)	380 (187 to 685)	15.19 (8.44 to 24.89)	21.23 (10.49 to 38.13)	1.24 (0.47 to 2.01)*
Afghanistan	327 (69 to 981)	2538 (772 to 5486)	17.57 (3.72 to 52.64)	43.44 (13.41 to 93.09)	2.95 (2.69 to 3.22)*
Algeria	1432 (727 to 2645)	7248 (3460 to 14207)	29.72 (15.35 to 54.03)	60.78 (28.98 to 119.48)	2.38 (2.24 to 2.53)*
Bahrain	41 (20 to 75)	262 (130 to 483)	43.34 (21.5 to 77.41)	76.59 (37.91 to 141.28)	1.95 (0.46 to 3.46)*
Egypt	1467 (744 to 2554)	5869 (2989 to 10319)	12.77 (6.55 to 22.06)	23.89 (12.23 to 41.85)	1.96 (1.76 to 2.16)*
Iran (Islamic Republic of)	1392 (709 to 2315)	10746 (4072 to 16534)	13.82 (6.94 to 22.89)	40.88 (15.79 to 62.92)	3.47 (3.12 to 3.82)*
Iraq	1032 (461 to 1952)	5724 (2779 to 10849)	31.02 (14 to 58.44)	58.56 (28.57 to 110.45)	2.06 (1.51 to 2.62)*
Jordan	283 (135 to 521)	1475 (702 to 2876)	43.72 (21.18 to 79.53)	51.24 (24.46 to 99.58)	0.6 (-0.43 to 1.65)
Kuwait	246 (153 to 369)	987 (606 to 1514)	67.79 (42.58 to 100.52)	56.65 (34.79 to 86.93)	-0.91 (-5.93 to 4.37)
Lebanon	301 (139 to 562)	840 (418 to 1539)	43.43 (20.15 to 80.72)	52.45 (26.15 to 96.12)	0.64 (0.37 to 0.91)*
Libya	432 (206 to 830)	2254 (1018 to 4298)	60.84 (29.42 to 114.37)	106.91 (47.97 to 204.92)	1.99 (0.8 to 3.18)*
Morocco	914 (444 to 1742)	3101 (1422 to 6476)	16.47 (8.15 to 31.05)	31.26 (14.3 to 65.44)	2.03 (1.75 to 2.31)*
Oman	56 (26 to 109)	347 (165 to 667)	19.13 (9.02 to 36.7)	32.67 (15.62 to 62.35)	1.8 (0.92 to 2.68)*

Palestine	103 (48 to 199)	445 (225 to 807)	30.62 (14.58 to 58.81)	39.47 (20.13 to 71.1)	0.73 (0.42 to 1.05)*
Qatar	42 (20 to 80)	312 (139 to 669)	57.69 (28.12 to 108.92)	50.25 (22.68 to 106.68)	-0.62 (-2.04 to 0.82)
Saudi Arabia	995 (433 to 1956)	13803 (6654 to 26075)	36.29 (15.88 to 71.7)	124.62 (60.21 to 235.75)	4.07 (3.77 to 4.37)*
Sudan	513 (125 to 1482)	2751 (1023 to 5614)	13.18 (3.28 to 37.21)	27.59 (10.45 to 55.78)	2.38 (2.11 to 2.65)*
Syrian Arab Republic	320 (105 to 887)	1330 (544 to 2679)	14.77 (4.92 to 40.59)	34.41 (13.89 to 70.18)	2.81 (2.01 to 3.63)*
Tunisia	561 (269 to 1051)	1984 (935 to 3783)	31.53 (15.33 to 58.24)	58.62 (27.45 to 112.88)	2.02 (1.81 to 2.23)*
Turkey	4010 (1818 to 7363)	9778 (4669 to 17761)	32.48 (14.99 to 59.11)	41.52 (19.66 to 75.89)	0.78 (0.46 to 1.1)*
United Arab Emirates	135 (54 to 266)	1141 (560 to 2152)	49.51 (20.03 to 95.33)	56.09 (26.73 to 105.89)	0.32 (-0.71 to 1.36)
Yemen	168 (56 to 414)	1339 (598 to 2584)	8.06 (2.72 to 19.96)	18.96 (8.53 to 36.12)	2.81 (2.09 to 3.53)*
Bangladesh	2841 (1276 to 5361)	12086 (4646 to 30845)	12.98 (5.97 to 24.14)	26.58 (10.29 to 67.36)	2.42 (2.07 to 2.76)*
Bhutan	16 (6 to 30)	52 (20 to 129)	12.43 (5.17 to 23.82)	25.22 (9.64 to 62.37)	2.27 (2.02 to 2.53)*
India	23753 (17464 to 34733)	105355 (76158 to 145618)	12.36 (9.13 to 18)	28.28 (20.45 to 38.97)	2.63 (2.1 to 3.17)*
Nepal	494 (197 to 984)	2224 (924 to 4650)	11.58 (4.7 to 22.94)	25.56 (10.79 to 52.72)	2.61 (2.38 to 2.84)*
Pakistan	4792 (2769 to 8028)	27312 (14259 to 47650)	22.03 (12.9 to 36.42)	46.23 (24.28 to 80.28)	2.36 (2.13 to 2.59)*
China	38529 (26621 to 50759)	91354 (63878 to 143464)	13.65 (9.43 to 17.91)	24.2 (16.87 to 38.18)	1.91 (1.69 to 2.14)*

Democratic People's Republic of Korea	1068 (488 to 2031)	2485 (1088 to 4679)	20.24 (9.29 to 38.28)	35.29 (15.37 to 66.7)	1.84 (1.7 to 1.97)*
Taiwan (Province of China)	3036 (2000 to 4392)	6021 (3837 to 8899)	56.36 (37.34 to 81.16)	87.91 (55.58 to 131.07)	1.4 (0.99 to 1.81)*
American Samoa	2 (1 to 3)	4 (2 to 7)	17.21 (8.75 to 31.34)	33.36 (16.85 to 59.91)	2.02 (1.78 to 2.26)*
Cook Islands	0 (0 to 0)	0 (0 to 0)	2.44 (1.07 to 4.95)	5.72 (2.48 to 11.13)	2.73 (1.63 to 3.84)*
Micronesia (Federated States of)	1 (1 to 2)	3 (1 to 6)	6.8 (3.11 to 12.72)	12.36 (5.83 to 24.39)	1.93 (1.85 to 2.02)*
Fiji	41 (20 to 76)	74 (34 to 146)	23.32 (11.61 to 42.85)	31.9 (14.72 to 63.16)	1.03 (0.42 to 1.65)*
Guam	1 (1 to 3)	5 (3 to 7)	4.37 (2.55 to 9.52)	12.92 (8.42 to 18.71)	3.66 (1.42 to 5.95)*
Kiribati	0 (0 to 0)	0 (0 to 0)	0.14 (0.05 to 0.39)	0.19 (0.06 to 0.61)	1.05 (0.95 to 1.14)*
Marshall Islands	0 (0 to 1)	2 (1 to 3)	5.84 (2.84 to 10.4)	12.04 (5.26 to 23.18)	2.32 (2.16 to 2.47)*
Nauru	0 (0 to 0)	0 (0 to 1)	9.87 (3.96 to 19.4)	18.18 (6.46 to 36.75)	1.98 (1.88 to 2.08)*
Niue	0 (0 to 0)	0 (0 to 0)	7.56 (3.55 to 14.33)	18.8 (9.44 to 35.97)	3.04 (2.63 to 3.46)*
Northern Mariana Islands	1 (0 to 2)	1 (1 to 2)	7.88 (3.72 to 14.98)	11.53 (5.87 to 21.25)	0.87 (0.27 to 1.47)*
Palau	0 (0 to 1)	1 (0 to 1)	9.78 (4.68 to 18.45)	15.6 (7.02 to 29.7)	1.45 (1.36 to 1.54)*
Papua New Guinea	42 (17 to 89)	172 (70 to 411)	5.13 (2.05 to 10.94)	6.93 (2.8 to 16.67)	0.95 (0.68 to 1.23)*

Samoa	7 (3 to 14)	22 (9 to 45)	24.05 (11.02 to 46.01)	49.6 (20.97 to 99.77)	2.37 (2.21 to 2.54)*
Solomon Islands	2 (1 to 5)	15 (7 to 30)	4.12 (1.27 to 9.05)	9.37 (4.27 to 18.82)	2.66 (2.43 to 2.9)*
Tokelau	0 (0 to 0)	0 (0 to 0)	7.52 (3.47 to 13.8)	20.05 (10.09 to 38.54)	3.37 (2.82 to 3.93)*
Tonga	1 (1 to 2)	2 (1 to 4)	5.85 (2.84 to 11.01)	9.58 (4.36 to 19.18)	1.54 (1.29 to 1.78)*
Tuvalu	0 (0 to 0)	0 (0 to 1)	6.29 (3.03 to 11.5)	12.24 (5.82 to 23.55)	2.17 (2.01 to 2.34)*
Vanuatu	1 (1 to 3)	6 (3 to 11)	4.71 (2.03 to 9.56)	8.34 (4.05 to 15.72)	1.81 (1.45 to 2.17)*
Cambodia	323 (141 to 606)	1594 (753 to 3108)	15.37 (6.87 to 28.44)	37.16 (17.61 to 72.22)	2.91 (2.82 to 3)*
Indonesia	6780 (3986 to 9491)	20982 (12520 to 32188)	16.48 (9.76 to 22.99)	26.27 (15.68 to 40.39)	1.54 (1.34 to 1.74)*
Lao People's Democratic Republic	118 (44 to 230)	503 (229 to 981)	14.83 (5.63 to 28.65)	27.3 (12.57 to 53.07)	1.98 (1.81 to 2.14)*
Malaysia	1056 (514 to 1998)	4209 (2082 to 7834)	26.07 (12.99 to 48.62)	50.63 (25.16 to 93.84)	2.14 (1.68 to 2.61)*
Maldives	4 (1 to 7)	15 (7 to 31)	10.56 (3.17 to 20.92)	12.61 (6.05 to 24.94)	0.44 (0.27 to 0.6)*
Mauritius	41 (31 to 55)	60 (43 to 79)	15.52 (11.46 to 20.4)	17.07 (12.37 to 22.6)	-0.4 (-5.2 to 4.66)
Myanmar	1693 (710 to 3308)	4185 (2006 to 7836)	19.12 (8.24 to 36.83)	27.71 (13.27 to 51.89)	1.2 (1.05 to 1.35)*
Philippines	4160 (2762 to 5966)	12676 (8267 to 19289)	31.69 (21.02 to 45.34)	46.32 (30.23 to 70.12)	1.2 (0.87 to 1.54)*
Seychelles	1 (1 to 2)	4 (2 to 6)	9.49 (4.97 to 16.33)	13.25 (6.51 to 23.01)	1.33 (1.02 to 1.63)*

Sri Lanka	875 (446 to 1538)	2184 (949 to 4267)	20.76 (10.7 to 36.24)	36.74 (15.92 to 72.03)	1.92 (1.17 to 2.67)*
Thailand	3492 (1841 to 6014)	9325 (4551 to 17415)	24.42 (12.99 to 41.68)	48.22 (23.14 to 90.56)	2.23 (1.58 to 2.89)*
Timor-Leste	15 (7 to 30)	51 (24 to 98)	9.68 (4.37 to 18.93)	18.38 (8.78 to 34.83)	2.06 (1.6 to 2.53)*
Viet Nam	3905 (1784 to 7618)	24911 (11167 to 48106)	26.41 (12.47 to 50.21)	89.58 (39.67 to 173.96)	4.03 (3.85 to 4.21)*
Angola	63 (27 to 125)	342 (128 to 737)	3.34 (1.45 to 6.55)	5.26 (2 to 11.33)	1.44 (0.85 to 2.04)*
Central African Republic	19 (9 to 36)	42 (17 to 86)	3.66 (1.71 to 6.86)	3.6 (1.48 to 7.33)	-0.09 (-0.24 to 0.07)
Congo	21 (9 to 41)	111 (44 to 233)	5.02 (2.23 to 9.49)	8.4 (3.36 to 17.47)	1.83 (1.12 to 2.54)*
Democratic Republic of the Congo	228 (93 to 468)	745 (270 to 1677)	3.28 (1.37 to 6.67)	4.23 (1.55 to 9.48)	0.8 (0.56 to 1.05)*
Equatorial Guinea	3 (1 to 5)	25 (8 to 58)	3.38 (1.47 to 6.54)	8.25 (2.79 to 18.61)	3.04 (2.2 to 3.88)*
Gabon	10 (4 to 19)	35 (14 to 74)	5.82 (2.68 to 10.9)	8.22 (3.29 to 17.44)	1.15 (0.9 to 1.41)*
Burundi	194 (84 to 378)	496 (210 to 1058)	16.83 (7.41 to 32.56)	17.37 (7.42 to 37)	0.06 (-0.11 to 0.24)
Comoros	15 (6 to 34)	47 (18 to 108)	16.33 (6.82 to 35.13)	24.61 (9.5 to 56.86)	1.24 (0.56 to 1.93)*
Djibouti	10 (4 to 21)	61 (21 to 154)	11.49 (4.55 to 25.19)	18.94 (6.61 to 47.82)	1.69 (1.52 to 1.85)*
Eritrea	87 (41 to 163)	320 (133 to 667)	12.84 (6.19 to 23.55)	21.06 (8.89 to 43.5)	1.58 (1.35 to 1.82)*
Ethiopia	3728 (1961 to 6325)	12227 (6313 to 25899)	36.8 (19.59 to 62.22)	48.49 (25.24 to 102.77)	0.87 (0.69 to 1.04)*

Kenya	218 (128 to 382)	1086 (612 to 2071)	4.88 (2.87 to 8.66)	9.15 (5.22 to 17.18)	1.95 (1.38 to 2.53)*
Madagascar	377 (180 to 695)	1428 (623 to 2833)	15.59 (7.61 to 28.41)	21.43 (9.46 to 42.34)	0.99 (0.88 to 1.09)*
Malawi	307 (145 to 582)	1175 (483 to 2577)	14.99 (7.24 to 27.82)	26.3 (11.12 to 56.12)	1.89 (1.59 to 2.2)*
Mozambique	355 (149 to 814)	1346 (519 to 3392)	12.27 (5.21 to 27.91)	19.95 (7.77 to 49.93)	1.56 (1.28 to 1.84)*
Rwanda	322 (151 to 594)	790 (324 to 1757)	21.96 (10.36 to 40.22)	24.01 (10 to 52.65)	0.14 (-0.52 to 0.8)
Somalia	178 (73 to 353)	583 (230 to 1200)	11.51 (4.79 to 22.61)	13.68 (5.43 to 28.09)	0.59 (0.45 to 0.73)*
South Sudan	126 (50 to 274)	422 (172 to 918)	11.31 (4.66 to 23.79)	19.89 (8.1 to 43.17)	1.89 (1.6 to 2.19)*
United Republic of Tanzania	828 (371 to 1618)	3110 (1307 to 6461)	15.82 (7.27 to 30.5)	22.94 (9.83 to 46.98)	1.2 (1.05 to 1.36)*
Uganda	395 (180 to 756)	3341 (1475 to 6732)	11.86 (5.52 to 22.4)	35.52 (16.1 to 70.28)	3.48 (3.22 to 3.74)*
Zambia	283 (134 to 512)	1419 (449 to 4355)	17.89 (8.65 to 31.94)	31.93 (10.5 to 94.84)	1.87 (1.72 to 2.03)*
Botswana	16 (6 to 36)	50 (18 to 121)	6.48 (2.54 to 14.63)	7.6 (2.72 to 18.22)	0.42 (-0.87 to 1.74)
Eswatini	11 (5 to 22)	31 (11 to 69)	7.23 (3.11 to 14.33)	11.85 (4.37 to 25.85)	1.57 (1.35 to 1.79)*
Lesotho	13 (5 to 30)	37 (15 to 77)	3.84 (1.48 to 8.92)	8.91 (3.68 to 18.08)	2.61 (1.88 to 3.35)*
Namibia	19 (9 to 37)	82 (34 to 168)	6.76 (3.23 to 12.83)	13.48 (5.74 to 27.26)	2.3 (1.91 to 2.68)*
South Africa	968 (729 to 1396)	1802 (1331 to 2814)	11.83 (8.92 to 16.75)	11.56 (8.56 to 17.92)	-0.07 (-1.26 to 1.13)

Zimbabwe	353 (172 to 659)	1398 (598 to 2839)	18.66 (9.37 to 34.3)	38.64 (16.83 to 77.87)	2.29 (1.15 to 3.44)*
Benin	24 (11 to 51)	85 (35 to 184)	2.61 (1.18 to 5.42)	3.11 (1.3 to 6.71)	0.55 (0.36 to 0.73)*
Burkina Faso	65 (27 to 135)	172 (73 to 352)	3.59 (1.51 to 7.44)	3.7 (1.59 to 7.48)	0.07 (-0.2 to 0.33)
Cabo Verde	1 (0 to 2)	10 (2 to 22)	1.48 (0.65 to 3.02)	6.96 (1.65 to 14.76)	5.12 (4.62 to 5.62)*
Cameroon	89 (39 to 179)	340 (143 to 673)	4.48 (1.97 to 8.82)	4.99 (2.13 to 9.82)	0.36 (0.11 to 0.6)*
Chad	26 (11 to 56)	89 (40 to 174)	2.33 (1.03 to 4.87)	2.88 (1.33 to 5.6)	0.67 (0.53 to 0.81)*
Coted'Ivoire	197 (93 to 381)	908 (408 to 1788)	8.28 (4 to 15.56)	14.56 (6.66 to 28.29)	1.83 (1.4 to 2.27)*
Gambia	8 (3 to 15)	38 (15 to 76)	4 (1.82 to 7.61)	6.94 (2.95 to 13.76)	1.75 (0.59 to 2.93)*
Ghana	9 (4 to 17)	42 (17 to 93)	0.29 (0.14 to 0.55)	0.49 (0.2 to 1.08)	1.79 (1.64 to 1.94)*
Guinea	56 (27 to 105)	194 (81 to 415)	4.42 (2.13 to 8.22)	6.41 (2.7 to 13.5)	1.16 (0.98 to 1.34)*
Guinea-Bissau	9 (4 to 17)	22 (10 to 42)	4.37 (1.96 to 8.49)	4.85 (2.22 to 9.21)	0.34 (0.17 to 0.5)*
Liberia	14 (6 to 28)	61 (26 to 121)	2.86 (1.19 to 5.78)	4.86 (2.09 to 9.57)	1.78 (1.45 to 2.12)*
Mali	235 (120 to 423)	776 (327 to 1608)	13.56 (7 to 24.02)	16.52 (7.08 to 33.8)	0.63 (0.42 to 0.84)*
Mauritania	11 (5 to 23)	40 (17 to 85)	2.88 (1.23 to 5.69)	4.45 (1.89 to 9.34)	1.38 (1.1 to 1.66)*
Niger	35 (15 to 74)	97 (41 to 204)	2.42 (1.05 to 5)	2.36 (1.02 to 4.92)	-0.08 (-0.49 to 0.33)

Nigeria	124 (68 to 222)	606 (308 to 1175)	0.75 (0.41 to 1.35)	1.25 (0.64 to 2.39)	1.57 (1.27 to 1.87)*
Sao Tome and Principe	0 (0 to 0)	0 (0 to 1)	0.34 (0.15 to 0.72)	0.67 (0.26 to 1.53)	2.13 (1.48 to 2.78)*
Senegal	48 (20 to 103)	131 (54 to 297)	3.35 (1.41 to 7.05)	3.86 (1.61 to 8.67)	0.32 (-0.32 to 0.97)
Sierra Leone	21 (8 to 47)	73 (30 to 154)	2.49 (0.99 to 5.4)	3.81 (1.59 to 7.97)	1.37 (0.96 to 1.79)*
Togo	27 (12 to 57)	87 (36 to 180)	3.8 (1.67 to 7.78)	4.3 (1.78 to 8.93)	0.39 (0.14 to 0.64)*

Table S3: DALYs of WCBA thyroid cancer in 1990 and 2021, and its ASR estimated AAPC from 1990 to 2021.

Location	Number, 95% UI		Age-standardized DALYs rate (per 100 000), 95% UI		AAPC of DALYs rate, No. (95% CI)
	1990	2021	1990	2021	
Global	127692 (105583 to 157611)	206508 (161917 to 272323)	10.31 (8.58 to 12.66)	10.38 (8.13 to 13.74)	-0.01 (-0.18 to 0.17)
**SDI regions**					
Low SDI	18192 (12728 to 25298)	39857 (27990 to 63165)	17.76 (12.5 to 24.75)	15.7 (11.07 to 24.73)	-0.43 (-0.52 to -0.34)*
Low-middle SDI	32136 (24376 to 44917)	68877 (49898 to 98395)	12.78 (9.78 to 17.76)	13.97 (10.17 to 19.82)	0.28 (0.12 to 0.44)*
Middle SDI	37089 (30013 to 46485)	59997 (46706 to 75041)	9.51 (7.72 to 11.87)	9.14 (7.1 to 11.47)	-0.15 (-0.3 to -0.01)*

High-middle SDI	23383 (19074 to 27804)	21086 (16910 to 27230)	9.02 (7.38 to 10.69)	5.88 (4.7 to 7.65)	-1.38 (-1.6 to -1.16)*
High SDI	16763 (15073 to 18850)	16546 (13790 to 20260)	7.13 (6.41 to 8.02)	5.89 (4.9 to 7.24)	-0.62 (-0.77 to -0.47)*
**GBD super regions**					
Central Europe, Eastern Europe, and Central Asia	9114 (8408 to 9914)	6884 (6051 to 7913)	8.93 (8.24 to 9.71)	5.86 (5.15 to 6.74)	-1.52 (-2.22 to -0.83)*
High-income	15952 (14327 to 17994)	13767 (11756 to 16282)	6.57 (5.9 to 7.41)	4.94 (4.21 to 5.84)	-0.89 (-1.06 to -0.72)*
Latin America and Caribbean	7531 (6916 to 8279)	13385 (11710 to 15451)	8.88 (8.17 to 9.72)	8.12 (7.1 to 9.37)	-0.29 (-0.48 to -0.11)*
North Africa and Middle East	5585 (3971 to 8623)	14110 (10369 to 19187)	8.41 (5.99 to 12.95)	8.8 (6.47 to 11.97)	0.2 (-0.01 to 0.41)
South Asia	36363 (26975 to 51820)	84402 (60511 to 120598)	14.96 (11.15 to 21.27)	17.31 (12.43 to 24.61)	0.48 (0.11 to 0.86)*
Southeast Asia, East Asia, and Oceania	39020 (28027 to 48321)	44792 (33692 to 58869)	9.8 (7.07 to 12.11)	7.63 (5.74 to 10.09)	-0.79 (-0.92 to -0.65)*
Sub-Saharan Africa	14127 (10014 to 19525)	29169 (19807 to 48936)	14.15 (10.12 to 19.47)	11.27 (7.7 to 18.78)	-0.74 (-0.88 to -0.6)*

**GBD regions**					
Central Asia	1445 (1285 to 1632)	1610 (1343 to 1910)	10.48 (9.33 to 11.83)	6.42 (5.36 to 7.61)	-1.62 (-2.96 to -0.27)*
Central Europe	3715 (3357 to 4129)	1705 (1434 to 2010)	11.55 (10.44 to 12.84)	5.24 (4.39 to 6.19)	-2.59 (-3.37 to -1.8)*
Eastern Europe	3955 (3549 to 4451)	3568 (2937 to 4375)	7.06 (6.33 to 7.93)	5.94 (4.89 to 7.3)	-0.97 (-2.05 to 0.12)
Australasia	274 (196 to 377)	337 (226 to 501)	5.01 (3.59 to 6.9)	4.12 (2.74 to 6.14)	-0.52 (-1.64 to 0.61)
High-income Asia Pacific	2908 (2348 to 3751)	2702 (2024 to 3700)	5.94 (4.77 to 7.72)	5.59 (4.15 to 7.7)	-0.16 (-0.54 to 0.22)
High-income North America	3762 (3303 to 4332)	4996 (4259 to 5943)	4.9 (4.3 to 5.63)	5.43 (4.63 to 6.45)	0.5 (0.37 to 0.62)*
Southern Latin America	1196 (943 to 1508)	1163 (886 to 1527)	10.01 (7.91 to 12.61)	6.28 (4.78 to 8.26)	-1.37 (-2.24 to -0.49)*
Western Europe	7812 (6898 to 8929)	4569 (3787 to 5557)	7.82 (6.91 to 8.95)	4.06 (3.35 to 4.95)	-2.12 (-2.24 to -1.99)*
Andean Latin America	1074 (766 to 1490)	2134 (1456 to 3051)	13.49 (9.69 to 18.61)	12.33 (8.42 to 17.59)	-0.2 (-0.51 to 0.11)
Caribbean	790 (615 to 1033)	1172 (842 to 1637)	9.54 (7.48 to 12.39)	9.55 (6.84 to 13.36)	0.04 (-0.45 to 0.54)
Central Latin America	3366 (3060 to 3730)	6427 (5430 to 7589)	9.92 (9.05 to 10.94)	9.22 (7.79 to 10.89)	-0.3 (-0.54 to -0.05)*

Tropical Latin America	2300 (2063 to 2586)	3652 (3245 to 4137)	6.68 (6.01 to 7.5)	5.57 (4.95 to 6.31)	-0.65 (-0.86 to -0.45)*
East Asia	24353 (17201 to 32120)	19022 (13145 to 28903)	8.26 (5.84 to 10.85)	4.9 (3.38 to 7.49)	-1.67 (-1.98 to -1.35)*
Oceania	74 (40 to 118)	160 (83 to 305)	5.6 (3.06 to 8.94)	4.87 (2.52 to 9.29)	-0.45 (-0.54 to -0.36)*
Southeast Asia	14593 (9838 to 18431)	25611 (17970 to 33778)	14.2 (9.71 to 17.81)	13.35 (9.37 to 17.64)	-0.17 (-0.27 to -0.07)*
Central Sub-Saharan Africa	475 (256 to 862)	1113 (521 to 2242)	4.72 (2.58 to 8.47)	4.06 (1.91 to 8.09)	-0.54 (-0.66 to -0.42)*
Eastern Sub-Saharan Africa	11351 (7630 to 16343)	22843 (14804 to 40928)	29.33 (19.84 to 42.17)	23.01 (14.98 to 41.12)	-0.8 (-0.88 to -0.71)*
Southern Sub-Saharan Africa	1100 (818 to 1471)	2336 (1514 to 3582)	9.85 (7.35 to 13.09)	11.12 (7.25 to 16.96)	0.4 (-0.22 to 1.02)
Western Sub-Saharan Africa	1201 (812 to 1717)	2877 (1870 to 4504)	3.22 (2.21 to 4.52)	2.71 (1.77 to 4.2)	-0.56 (-0.77 to -0.36)*
**Countries and territories**					
Armenia	60 (41 to 85)	64 (44 to 91)	7.76 (5.34 to 10.77)	7.71 (5.24 to 10.92)	-0.28 (-1.29 to 0.74)

Azerbaijan	97 (54 to 160)	120 (64 to 212)	6.45 (3.68 to 10.42)	4.14 (2.21 to 7.32)	-1.55 (-2.11 to -0.98)*
Georgia	114 (79 to 164)	131 (89 to 183)	8.54 (5.91 to 12.23)	14.19 (9.6 to 19.88)	1.37 (-0.41 to 3.19)
Kazakhstan	765 (664 to 884)	582 (457 to 730)	20.8 (18.01 to 24.1)	11.24 (8.84 to 14.1)	-1.58 (-4.25 to 1.17)
Kyrgyzstan	202 (137 to 286)	191 (127 to 276)	21.72 (14.97 to 30.42)	11.44 (7.63 to 16.47)	-2.38 (-4.15 to -0.57)*
Mongolia	48 (24 to 84)	85 (48 to 140)	12.63 (6.52 to 21.67)	9.34 (5.29 to 15.45)	-1.18 (-2.07 to -0.29)*
Tajikistan	1 (1 to 2)	2 (1 to 3)	0.16 (0.08 to 0.28)	0.09 (0.04 to 0.15)	-1.94 (-2.36 to -1.51)*
Turkmenistan	77 (67 to 87)	109 (79 to 148)	10.94 (9.57 to 12.46)	8.77 (6.37 to 11.95)	-0.61 (-3.27 to 2.13)
Uzbekistan	80 (55 to 113)	327 (221 to 472)	2.12 (1.48 to 2.95)	3.58 (2.42 to 5.15)	1.74 (1.04 to 2.44)*
Albania	47 (28 to 76)	27 (14 to 50)	6.68 (3.92 to 10.64)	4.12 (2.03 to 7.61)	-1.71 (-2.29 to -1.12)*
Bosnia and Herzegovina	72 (50 to 99)	33 (21 to 52)	6.4 (4.42 to 8.84)	3.75 (2.35 to 5.91)	-1.87 (-2.44 to -1.29)*
Bulgaria	209 (151 to 283)	117 (81 to 163)	9.11 (6.53 to 12.37)	6.15 (4.25 to 8.66)	-1.15 (-2.88 to 0.62)
Croatia	121 (85 to 168)	47 (30 to 71)	9.42 (6.59 to 13.07)	4.28 (2.68 to 6.46)	-2.75 (-4.12 to -1.35)*
Czechia	316 (224 to 434)	158 (101 to 236)	11.05 (7.79 to 15.22)	5.15 (3.23 to 7.8)	-2.43 (-3.33 to -1.52)*

Hungary	403 (294 to 535)	151 (105 to 208)	13.98 (10.16 to 18.66)	5.28 (3.61 to 7.4)	-2.92 (-4.17 to -1.66)*
Montenegro	13 (10 to 19)	11 (7 to 16)	8.8 (6.26 to 12.73)	6.33 (4.24 to 9.7)	-0.9 (-1.48 to -0.32)*
North Macedonia	41 (29 to 55)	27 (17 to 42)	8.16 (5.77 to 10.75)	4.25 (2.71 to 6.71)	-2.15 (-2.8 to -1.5)*
Poland	1515 (1390 to 1676)	660 (537 to 805)	15.46 (14.2 to 17.08)	6 (4.88 to 7.32)	-2.8 (-4.11 to -1.48)*
Romania	537 (385 to 728)	229 (152 to 331)	9.5 (6.82 to 12.9)	4.35 (2.85 to 6.36)	-2.4 (-3.74 to -1.05)*
Serbia	182 (99 to 313)	111 (60 to 192)	7.43 (4.06 to 12.83)	4.58 (2.43 to 8.02)	-1.33 (-1.76 to -0.9)*
Slovakia	151 (90 to 242)	94 (50 to 162)	11.02 (6.59 to 17.71)	5.99 (3.09 to 10.4)	-1.67 (-2.56 to -0.77)*
Slovenia	48 (35 to 66)	14 (9 to 21)	9.29 (6.69 to 12.64)	2.53 (1.56 to 3.78)	-4.28 (-5.59 to -2.95)*
Belarus	282 (189 to 407)	155 (94 to 242)	11.03 (7.41 to 15.89)	5.76 (3.48 to 9.03)	-2.68 (-7.09 to 1.94)
Estonia	51 (35 to 72)	16 (10 to 23)	12.63 (8.66 to 18)	4.62 (2.98 to 6.77)	-3.53 (-4.78 to -2.26)*
Latvia	81 (56 to 113)	30 (20 to 44)	11.66 (8.05 to 16.38)	5.96 (3.95 to 8.75)	-2.25 (-4 to -0.47)*
Lithuania	111 (78 to 158)	36 (24 to 51)	11.66 (8.13 to 16.59)	5.03 (3.36 to 7.13)	-2.92 (-4.13 to -1.69)*
Republic of Moldova	55 (46 to 66)	38 (29 to 49)	5.01 (4.21 to 6)	3.49 (2.68 to 4.57)	-1.46 (-2.15 to -0.77)*

Russian Federation	2622 (2404 to 2899)	2523 (2131 to 3019)	7.1 (6.52 to 7.84)	5.99 (5.06 to 7.16)	-0.76 (-3.17 to 1.71)
Ukraine	753 (508 to 1078)	771 (396 to 1351)	5.66 (3.82 to 8.12)	6.11 (3.12 to 10.74)	0.16 (-0.84 to 1.17)
Australia	237 (160 to 340)	308 (198 to 469)	5.22 (3.52 to 7.47)	4.48 (2.87 to 6.85)	-0.35 (-1.73 to 1.04)
New Zealand	36 (26 to 49)	30 (20 to 42)	4.04 (2.93 to 5.48)	2.25 (1.53 to 3.16)	-1.99 (-4.24 to 0.32)
Brunei Darussalam	7 (3 to 12)	13 (7 to 21)	12.46 (6.14 to 22.18)	9.51 (5.3 to 15.89)	-0.91 (-1.47 to -0.36)*
Japan	1939 (1646 to 2337)	1601 (1275 to 2033)	5.23 (4.43 to 6.31)	4.99 (3.97 to 6.32)	-0.04 (-0.35 to 0.27)
Singapore	62 (43 to 87)	55 (34 to 85)	6.81 (4.73 to 9.47)	3.15 (1.94 to 4.85)	-2.31 (-2.9 to -1.72)*
Republic of Korea	900 (492 to 1649)	1033 (540 to 1820)	7.88 (4.36 to 14.36)	7.17 (3.67 to 12.79)	-0.28 (-0.55 to -0.01)*
Canada	538 (368 to 742)	396 (250 to 602)	7.02 (4.81 to 9.67)	4.2 (2.64 to 6.42)	-1.54 (-2.43 to -0.65)*
Greenland	2 (1 to 3)	1 (0 to 1)	13.29 (6.41 to 22.97)	5.41 (2.6 to 11.23)	-2.98 (-3.52 to -2.43)*
United States of America	3222 (2838 to 3705)	4600 (3933 to 5471)	4.66 (4.11 to 5.35)	5.57 (4.76 to 6.63)	0.59 (0.09 to 1.09)*
Argentina	779 (564 to 1050)	769 (528 to 1074)	9.85 (7.14 to 13.28)	6.14 (4.2 to 8.58)	-1.38 (-2.34 to -0.41)*
Chile	338 (237 to 467)	318 (215 to 457)	10.37 (7.28 to 14.25)	6.25 (4.22 to 8.99)	-1.55 (-2.27 to -0.82)*

Uruguay	79 (55 to 112)	75 (51 to 106)	10.42 (7.22 to 14.8)	8.35 (5.66 to 11.81)	-0.46 (-0.85 to -0.06)*
Andorra	1 (0 to 2)	1 (0 to 2)	6.01 (2.85 to 10.82)	3.89 (1.71 to 7.35)	-1.49 (-1.71 to -1.27)*
Austria	214 (152 to 295)	106 (68 to 157)	10.31 (7.29 to 14.22)	4.46 (2.84 to 6.69)	-2.72 (-3.31 to -2.13)*
Belgium	188 (136 to 261)	117 (76 to 168)	7.4 (5.34 to 10.25)	3.99 (2.59 to 5.79)	-2.26 (-3.41 to -1.09)*
Cyprus	18 (9 to 33)	12 (5 to 26)	9.07 (4.55 to 16.18)	2.71 (1.23 to 5.98)	-3.67 (-4.29 to -3.04)*
Denmark	85 (62 to 113)	27 (18 to 40)	5.9 (4.27 to 7.86)	1.85 (1.2 to 2.7)	-3.85 (-5.11 to -2.57)*
Finland	140 (102 to 187)	43 (28 to 65)	9.81 (7.1 to 13.18)	3.36 (2.13 to 5.04)	-3.15 (-4.75 to -1.51)*
France	1264 (886 to 1732)	826 (500 to 1262)	8.51 (5.96 to 11.66)	5.04 (3.02 to 7.78)	-1.55 (-1.79 to -1.3)*
Germany	1869 (1350 to 2518)	891 (562 to 1341)	8.99 (6.48 to 12.13)	4.44 (2.78 to 6.72)	-2.33 (-2.8 to -1.85)*
Greece	119 (98 to 147)	89 (69 to 116)	4.53 (3.73 to 5.58)	3.12 (2.41 to 4.09)	-1.41 (-2.19 to -0.63)*
Iceland	12 (8 to 16)	6 (4 to 8)	18.82 (13.51 to 25.78)	6.42 (4.19 to 9.42)	-3.37 (-4.91 to -1.81)*
Ireland	50 (36 to 69)	46 (30 to 68)	5.86 (4.21 to 8.06)	3.19 (2.07 to 4.77)	-1.86 (-2.66 to -1.05)*
Israel	93 (65 to 127)	89 (59 to 127)	8.07 (5.69 to 11.05)	3.73 (2.48 to 5.36)	-1.97 (-4.79 to 0.92)

Italy	1572 (1345 to 1867)	859 (676 to 1099)	10.44 (8.93 to 12.4)	5.23 (4.1 to 6.71)	-2.07 (-2.67 to -1.45)*
Luxembourg	9 (7 to 10)	6 (4 to 8)	8.44 (7.07 to 9.98)	3.15 (2.36 to 4.22)	-3.33 (-4.36 to -2.29)*
Malta	8 (6 to 11)	6 (4 to 8)	7.58 (5.31 to 10.41)	4.91 (3.22 to 7.12)	-1.44 (-3.06 to 0.2)
Monaco	1 (0 to 1)	1 (0 to 1)	6.17 (3.18 to 11)	6.04 (2.79 to 11.07)	-0.09 (-0.2 to 0.03)
Netherlands	271 (194 to 368)	183 (123 to 262)	6.59 (4.73 to 8.97)	4.44 (2.97 to 6.41)	-1.35 (-1.54 to -1.16)*
Norway	63 (55 to 72)	24 (20 to 30)	5.66 (4.96 to 6.46)	1.75 (1.44 to 2.16)	-3.87 (-5.47 to -2.24)*
Portugal	207 (146 to 286)	131 (85 to 195)	7.99 (5.64 to 11.09)	4.31 (2.77 to 6.45)	-2.17 (-3.24 to -1.09)*
San Marino	0 (0 to 1)	0 (0 to 1)	7.39 (3.79 to 12.5)	4.51 (1.74 to 8.77)	-1.7 (-2.02 to -1.38)*
Spain	645 (461 to 879)	329 (214 to 480)	6.73 (4.81 to 9.17)	2.52 (1.61 to 3.75)	-3.33 (-3.62 to -3.05)*
Sweden	96 (72 to 128)	51 (35 to 72)	4.03 (3.02 to 5.36)	2.03 (1.37 to 2.89)	-2.4 (-3.43 to -1.36)*
Switzerland	166 (120 to 225)	47 (30 to 70)	8.7 (6.27 to 11.77)	1.97 (1.26 to 2.96)	-4.85 (-6.39 to -3.28)*
United Kingdom	714 (665 to 775)	676 (612 to 751)	4.82 (4.49 to 5.23)	3.85 (3.49 to 4.28)	-0.74 (-1.2 to -0.27)*

Bolivia (Plurinational State of)	309 (141 to 545)	519 (255 to 933)	23.58 (10.85 to 41.24)	17.46 (8.62 to 31.26)	-0.97 (-1.14 to -0.8)*
Ecuador	224 (161 to 304)	618 (385 to 942)	10.57 (7.68 to 14.25)	13.41 (8.36 to 20.42)	1.24 (0.78 to 1.69)*
Peru	541 (320 to 841)	997 (521 to 1690)	11.92 (7.14 to 18.39)	10.24 (5.36 to 17.36)	-0.16 (-1.73 to 1.44)
Antigua and Barbuda	2 (1 to 2)	3 (2 to 3)	10.6 (8.83 to 12.57)	9.68 (8.05 to 11.77)	-0.1 (-0.72 to 0.52)
Bahamas	8 (7 to 9)	16 (11 to 21)	12.5 (10.45 to 14.82)	13.74 (10.07 to 18.48)	0.11 (-0.55 to 0.77)
Barbados	6 (5 to 7)	7 (5 to 10)	9.36 (7.84 to 11.08)	8.8 (6.37 to 11.95)	-0.09 (-0.97 to 0.8)
Belize	1 (1 to 2)	7 (5 to 8)	4.58 (3.85 to 5.39)	6.08 (4.92 to 7.44)	0.66 (-0.05 to 1.38)
Bermuda	2 (1 to 2)	1 (1 to 2)	8.76 (5.95 to 12.22)	6.93 (4.33 to 10.75)	-0.91 (-1.62 to -0.19)*
Cuba	230 (161 to 312)	217 (143 to 312)	8.07 (5.69 to 10.93)	7.44 (4.88 to 10.79)	-0.28 (-1.3 to 0.75)
Dominica	1 (1 to 1)	1 (0 to 2)	6.02 (3.62 to 9.51)	5.9 (3.01 to 10.17)	-0.07 (-0.3 to 0.16)
Dominican Republic	123 (74 to 203)	228 (117 to 405)	7.99 (4.87 to 13.01)	8.1 (4.17 to 14.34)	-0.02 (-0.85 to 0.8)
Grenada	3 (2 to 4)	3 (2 to 5)	17.23 (11.81 to 24.39)	13.01 (8.51 to 19.15)	-1.08 (-2.03 to -0.12)*

Guyana	12 (8 to 18)	20 (12 to 31)	7.51 (5.1 to 10.73)	10.13 (6.1 to 16.07)	0.88 (-0.78 to 2.58)
Haiti	229 (98 to 430)	434 (200 to 813)	17.34 (7.68 to 32.19)	12.99 (6.06 to 24.21)	-0.82 (-1.11 to -0.53)*
Jamaica	32 (22 to 44)	82 (49 to 129)	6.85 (4.79 to 9.42)	10.73 (6.49 to 16.92)	1.49 (0.58 to 2.41)*
Puerto Rico	67 (48 to 92)	42 (27 to 64)	7.06 (4.99 to 9.66)	4.93 (3.14 to 7.49)	-1.34 (-2.57 to -0.1)*
Saint Kitts and Nevis	1 (1 to 1)	1 (1 to 1)	9.73 (8.25 to 11.48)	4.54 (3.24 to 6.1)	-2.45 (-3.36 to -1.54)*
Saint Lucia	5 (4 to 6)	8 (6 to 10)	17.8 (15.24 to 20.89)	15.42 (11.75 to 20.05)	-0.46 (-1.42 to 0.51)
Saint Vincent and the Grenadines	4 (3 to 4)	6 (5 to 8)	17.28 (14.58 to 20.35)	21.58 (17.08 to 26.92)	0.53 (-0.25 to 1.32)
Suriname	9 (5 to 14)	14 (7 to 24)	10.06 (5.84 to 15.98)	9.26 (4.87 to 15.83)	-0.2 (-1.25 to 0.86)
Trinidad and Tobago	28 (24 to 33)	42 (30 to 58)	10.26 (8.79 to 11.97)	11.06 (7.78 to 15.29)	0.51 (-0.21 to 1.23)
United States Virgin Islands	2 (1 to 3)	1 (0 to 1)	4.98 (2.69 to 8.3)	3.16 (1.36 to 6.78)	-1.28 (-1.64 to -0.92)*
Colombia	977 (705 to 1301)	1396 (928 to 2071)	13.3 (9.7 to 17.55)	10.5 (6.99 to 15.57)	-0.79 (-1.1 to -0.48)*
Costa Rica	84 (61 to 114)	107 (72 to 157)	12.59 (9.14 to 16.92)	7.99 (5.34 to 11.65)	-1.85 (-3.16 to -0.53)*

El Salvador	119 (85 to 159)	188 (125 to 271)	11.52 (8.21 to 15.22)	10.74 (7.16 to 15.49)	-0.25 (-0.58 to 0.08)
Guatemala	186 (162 to 212)	399 (318 to 492)	12.42 (10.78 to 14.2)	10.27 (8.16 to 12.68)	-0.48 (-1.4 to 0.45)
Honduras	40 (22 to 68)	105 (49 to 198)	4.76 (2.66 to 8.01)	4.29 (2.05 to 8.02)	-0.33 (-0.65 to -0.01)*
Mexico	1554 (1473 to 1644)	3399 (2775 to 4063)	9.01 (8.54 to 9.53)	9.31 (7.6 to 11.12)	0.2 (-0.14 to 0.53)
Nicaragua	47 (27 to 73)	101 (55 to 174)	6.76 (4.01 to 10.5)	5.86 (3.19 to 10.06)	-0.6 (-1.02 to -0.17)*
Panama	96 (67 to 133)	88 (56 to 132)	17.2 (12.07 to 23.65)	8.2 (5.26 to 12.33)	-2.25 (-3.51 to -0.97)*
Venezuela (Bolivarian Republic of)	265 (228 to 305)	644 (450 to 887)	6.69 (5.76 to 7.73)	8.5 (5.94 to 11.71)	0.94 (0.3 to 1.58)*
Brazil	2222 (1983 to 2500)	3505 (3095 to 3989)	6.61 (5.91 to 7.42)	5.49 (4.85 to 6.26)	-0.66 (-0.87 to -0.45)*
Paraguay	79 (45 to 126)	147 (76 to 254)	9.88 (5.73 to 15.75)	8.25 (4.29 to 14.15)	-0.48 (-1.02 to 0.07)
Afghanistan	263 (58 to 796)	1249 (394 to 2550)	13.71 (3.01 to 41.41)	20.83 (6.6 to 41.89)	1.35 (1.12 to 1.57)*
Algeria	520 (273 to 940)	1329 (649 to 2542)	10.61 (5.69 to 18.82)	11.24 (5.47 to 21.58)	0.22 (0.01 to 0.43)*
Bahrain	12 (6 to 21)	38 (19 to 69)	12.91 (6.77 to 21.98)	11.27 (5.66 to 20.32)	-0.44 (-1.57 to 0.71)

Egypt	664 (352 to 1097)	1310 (696 to 2285)	5.75 (3.1 to 9.39)	5.35 (2.86 to 9.28)	-0.25 (-0.5 to 0)*
Iran (Islamic Republic of)	322 (183 to 501)	1443 (583 to 2153)	3.19 (1.8 to 4.91)	5.59 (2.3 to 8.33)	1.9 (1.44 to 2.35)*
Iraq	377 (174 to 695)	1083 (530 to 1990)	11.17 (5.25 to 20.44)	11.03 (5.44 to 20.15)	-0.06 (-0.33 to 0.21)
Jordan	88 (43 to 154)	234 (112 to 452)	13.25 (6.61 to 22.84)	8.09 (3.87 to 15.57)	-1.51 (-2.36 to -0.66)*
Kuwait	45 (29 to 65)	117 (72 to 182)	12.36 (8.16 to 17.72)	6.87 (4.2 to 10.64)	-2.28 (-7.19 to 2.9)
Lebanon	84 (40 to 152)	117 (57 to 217)	11.99 (5.75 to 21.65)	7.38 (3.62 to 13.68)	-1.58 (-1.93 to -1.22)*
Libya	131 (65 to 245)	440 (205 to 813)	18.13 (9.12 to 33.26)	20.93 (9.69 to 38.85)	0.52 (0.07 to 0.97)*
Morocco	419 (209 to 776)	757 (350 to 1525)	7.45 (3.81 to 13.58)	7.64 (3.53 to 15.45)	0.05 (-0.2 to 0.29)
Oman	17 (8 to 32)	51 (23 to 99)	5.73 (2.83 to 10.79)	4.93 (2.26 to 9.45)	-0.24 (-0.49 to 0.02)
Palestine	34 (16 to 63)	84 (43 to 149)	9.99 (4.92 to 18.41)	7.4 (3.83 to 13.01)	-1.03 (-1.44 to -0.61)*
Qatar	10 (5 to 19)	38 (17 to 82)	14.29 (7.29 to 26.17)	6.23 (2.8 to 13.43)	-2.29 (-4.15 to -0.4)*
Saudi Arabia	395 (182 to 744)	2268 (1086 to 4208)	14.3 (6.64 to 27.03)	20.74 (9.98 to 38.59)	1.19 (1.03 to 1.34)*
Sudan	287 (74 to 832)	794 (300 to 1551)	7.3 (1.92 to 20.71)	7.96 (3.06 to 15.42)	0.26 (0.13 to 0.39)*

Syrian Arab Republic	115 (40 to 311)	236 (98 to 458)	5.21 (1.84 to 14.01)	6.03 (2.45 to 11.88)	0.54 (-0.1 to 1.19)
Tunisia	159 (78 to 292)	312 (150 to 583)	8.87 (4.43 to 15.98)	9.3 (4.43 to 17.53)	0.07 (-0.12 to 0.25)
Turkey	1503 (710 to 2662)	1527 (761 to 2809)	12.1 (5.82 to 21.24)	6.49 (3.2 to 12.03)	-1.99 (-2.39 to -1.58)*
United Arab Emirates	46 (19 to 87)	211 (104 to 386)	17.03 (7.15 to 31.66)	10.6 (5.07 to 19.41)	-1.5 (-2.12 to -0.87)*
Yemen	91 (33 to 219)	459 (210 to 842)	4.39 (1.61 to 10.62)	6.55 (3.02 to 11.89)	1.35 (0.84 to 1.87)*
Bangladesh	3547 (1663 to 6375)	6010 (2407 to 14936)	15.87 (7.6 to 28.16)	13.16 (5.31 to 32.47)	-0.46 (-0.84 to -0.08)*
Bhutan	19 (8 to 37)	27 (10 to 67)	15.05 (6.56 to 28.08)	13.21 (5.19 to 32.59)	-0.46 (-0.63 to -0.3)*
India	26499 (19154 to 39198)	56440 (40730 to 78843)	13.68 (9.93 to 20.13)	15.12 (10.92 to 21.05)	0.26 (-0.26 to 0.78)
Nepal	635 (260 to 1225)	1311 (556 to 2732)	14.71 (6.16 to 28.13)	14.98 (6.48 to 30.66)	0.07 (-0.05 to 0.18)
Pakistan	5662 (3439 to 9383)	20614 (11308 to 34853)	25.48 (15.7 to 41.5)	34.6 (19.05 to 58.09)	1.02 (0.89 to 1.15)*
China	23020 (15988 to 30733)	17517 (11855 to 27200)	8.1 (5.63 to 10.77)	4.68 (3.16 to 7.31)	-1.77 (-2.07 to -1.47)*
Democratic People's Republic of Korea	548 (266 to 1014)	698 (323 to 1295)	10.26 (5 to 18.93)	9.83 (4.53 to 18.31)	-0.13 (-0.18 to -0.08)*

Taiwan (Province of China)	785 (549 to 1076)	806 (518 to 1177)	14.95 (10.55 to 20.39)	11.73 (7.45 to 17.36)	-0.81 (-1.22 to -0.41)*
American Samoa	1 (0 to 1)	1 (1 to 2)	8.3 (4.43 to 14.53)	11.09 (5.81 to 19.21)	0.91 (0.32 to 1.5)*
Cook Islands	0 (0 to 0)	0 (0 to 0)	0.95 (0.45 to 1.85)	1.09 (0.48 to 2.06)	0.42 (-0.54 to 1.4)
Micronesia (Federated States of)	1 (1 to 2)	1 (1 to 3)	5.74 (2.74 to 10.61)	5.73 (2.91 to 10.68)	0.01 (-0.04 to 0.06)
Fiji	25 (13 to 44)	30 (15 to 56)	14.32 (7.74 to 25.14)	13.18 (6.33 to 24.53)	-0.31 (-0.53 to -0.08)*
Guam	0 (0 to 1)	1 (1 to 2)	1.4 (0.88 to 2.98)	2.96 (2.03 to 4.09)	2.86 (1.18 to 4.56)*
Kiribati	0 (0 to 0)	0 (0 to 0)	0.15 (0.06 to 0.41)	0.13 (0.05 to 0.4)	-0.43 (-0.52 to -0.33)*
Marshall Islands	0 (0 to 1)	1 (0 to 2)	4.75 (2.47 to 8.28)	6.39 (2.88 to 12.2)	0.94 (0.87 to 1.01)*
Nauru	0 (0 to 0)	0 (0 to 0)	6.98 (2.95 to 13.33)	8.19 (3.04 to 15.96)	0.5 (0.4 to 0.59)*
Niue	0 (0 to 0)	0 (0 to 0)	3.89 (1.94 to 7.24)	5.62 (2.89 to 10.65)	1.38 (1.2 to 1.55)*
Northern Mariana Islands	0 (0 to 1)	0 (0 to 1)	2.73 (1.35 to 5.03)	2.76 (1.41 to 5.01)	0 (-0.35 to 0.35)
Palau	0 (0 to 0)	0 (0 to 0)	4.74 (2.4 to 8.36)	4.79 (2.24 to 8.85)	0 (-0.14 to 0.14)

Papua New Guinea	32 (13 to 67)	97 (41 to 229)	3.98 (1.63 to 8.18)	3.9 (1.67 to 9.29)	0 (-0.19 to 0.19)
Samoa	4 (2 to 8)	8 (3 to 15)	13.97 (6.71 to 25.82)	17.37 (7.7 to 33.77)	0.72 (0.64 to 0.8)*
Solomon Islands	2 (1 to 4)	8 (4 to 17)	3.72 (1.14 to 7.86)	5.31 (2.54 to 10.56)	1.15 (0.89 to 1.41)*
Tokelau	0 (0 to 0)	0 (0 to 0)	4.96 (2.43 to 9.03)	6.46 (3.28 to 12.23)	1.02 (0.67 to 1.38)*
Tonga	1 (0 to 1)	1 (0 to 1)	2.98 (1.52 to 5.34)	3.11 (1.45 to 6.09)	0.11 (-0.03 to 0.25)
Tuvalu	0 (0 to 0)	0 (0 to 0)	5.42 (2.73 to 9.91)	5.21 (2.66 to 9.5)	-0.11 (-0.21 to -0.01)*
Vanuatu	1 (0 to 2)	3 (2 to 6)	3.63 (1.6 to 7.18)	4.61 (2.41 to 8.51)	0.74 (0.4 to 1.07)*
Cambodia	331 (149 to 595)	697 (341 to 1300)	15.95 (7.36 to 28.29)	16.48 (8.11 to 30.59)	0.15 (-0.03 to 0.33)
Indonesia	5225 (3058 to 7213)	8499 (5054 to 12935)	12.79 (7.55 to 17.58)	10.6 (6.31 to 16.17)	-0.59 (-0.67 to -0.51)*
Lao People's Democratic Republic	143 (54 to 278)	261 (122 to 483)	18.13 (6.97 to 35.04)	14.32 (6.75 to 26.36)	-0.77 (-0.9 to -0.64)*
Malaysia	551 (280 to 996)	1041 (537 to 1913)	13.8 (7.19 to 24.42)	12.62 (6.56 to 23.04)	-0.21 (-0.42 to 0)*
Maldives	3 (1 to 5)	3 (2 to 6)	7.5 (2.33 to 14.72)	2.72 (1.34 to 5.39)	-3.31 (-3.43 to -3.19)*

Mauritius	18 (15 to 21)	15 (12 to 19)	6.78 (5.69 to 8.11)	4.26 (3.36 to 5.31)	-2.27 (-6.6 to 2.26)
Myanmar	1693 (736 to 3287)	1861 (930 to 3355)	19.33 (8.64 to 37.02)	12.26 (6.12 to 22.12)	-1.5 (-1.6 to -1.39)*
Philippines	2419 (1732 to 3199)	5008 (3639 to 7174)	18.65 (13.24 to 24.67)	18.35 (13.35 to 26.12)	-0.05 (-0.31 to 0.21)
Seychelles	1 (0 to 1)	1 (0 to 2)	4.83 (2.69 to 7.94)	3.54 (1.8 to 6.02)	-0.66 (-0.83 to -0.49)*
Sri Lanka	438 (236 to 746)	459 (203 to 875)	10.49 (5.73 to 17.77)	7.69 (3.39 to 14.73)	-0.86 (-1.74 to 0.03)
Thailand	1572 (895 to 2658)	1885 (972 to 3403)	11.12 (6.39 to 18.6)	9.55 (4.84 to 17.36)	-0.39 (-1.06 to 0.27)
Timor-Leste	15 (7 to 29)	26 (13 to 49)	9.54 (4.48 to 18.27)	9.38 (4.57 to 17.51)	-0.04 (-0.6 to 0.52)
Viet Nam	2164 (1020 to 4086)	5818 (2712 to 10880)	14.86 (7.28 to 27.22)	21.02 (9.67 to 39.57)	1.12 (1.01 to 1.22)*
Angola	94 (43 to 179)	284 (110 to 611)	4.97 (2.33 to 9.35)	4.34 (1.71 to 9.28)	-0.46 (-1.03 to 0.11)
Central African Republic	32 (15 to 59)	57 (24 to 116)	6.2 (3.01 to 11.27)	4.95 (2.07 to 9.87)	-0.71 (-0.95 to -0.48)*
Congo	28 (13 to 52)	82 (34 to 165)	6.58 (3.05 to 12.02)	6.22 (2.62 to 12.41)	-0.17 (-1.08 to 0.75)
Democratic Republic of the Congo	306 (131 to 600)	656 (244 to 1446)	4.38 (1.91 to 8.53)	3.7 (1.39 to 8.15)	-0.57 (-0.77 to -0.38)*

Equatorial Guinea	4 (2 to 8)	14 (5 to 30)	5.21 (2.35 to 10.08)	4.48 (1.62 to 9.92)	-0.44 (-1.23 to 0.35)
Gabon	11 (5 to 20)	21 (9 to 43)	6.3 (3.08 to 11.61)	4.85 (2.05 to 10.14)	-0.74 (-1.01 to -0.48)*
Burundi	296 (134 to 562)	518 (225 to 1123)	25.51 (11.77 to 48.19)	17.82 (7.85 to 38.78)	-1.18 (-1.48 to -0.87)*
Comoros	19 (8 to 41)	38 (15 to 85)	20.11 (8.68 to 41.9)	19.74 (7.86 to 44.19)	-0.22 (-2.15 to 1.75)
Djibouti	11 (4 to 25)	45 (16 to 111)	13.03 (5.35 to 28.41)	14.05 (5.03 to 34.87)	0.21 (-0.13 to 0.54)
Eritrea	134 (67 to 241)	319 (138 to 650)	19.56 (10 to 34.6)	20.84 (9.13 to 41.98)	0.15 (-0.08 to 0.38)
Ethiopia	6477 (3505 to 10795)	9581 (5236 to 19917)	63.16 (34.63 to 104.89)	37.27 (20.52 to 77.83)	-1.71 (-1.86 to -1.56)*
Kenya	210 (124 to 368)	729 (416 to 1398)	4.62 (2.74 to 8.26)	6.07 (3.51 to 11.48)	0.89 (0.72 to 1.06)*
Madagascar	488 (241 to 860)	1283 (588 to 2435)	19.87 (10.11 to 34.52)	18.91 (8.8 to 35.59)	-0.14 (-0.24 to -0.05)*
Malawi	421 (212 to 790)	1011 (438 to 2200)	20.13 (10.35 to 36.95)	22.16 (9.91 to 46.99)	0.38 (0.23 to 0.54)*
Mozambique	500 (226 to 1125)	1380 (540 to 3411)	17.11 (7.83 to 37.99)	20 (7.92 to 49.5)	0.53 (0.33 to 0.73)*
Rwanda	516 (247 to 920)	617 (270 to 1350)	34.81 (16.75 to 61.6)	18.61 (8.26 to 39.99)	-2 (-2.15 to -1.85)*
Somalia	279 (119 to 532)	765 (314 to 1514)	18.07 (7.86 to 34.18)	17.6 (7.25 to 34.69)	-0.08 (-0.21 to 0.05)

South Sudan	161 (67 to 337)	383 (168 to 800)	14.15 (6.15 to 28.69)	17.76 (7.74 to 37.22)	0.78 (0.48 to 1.08)*
United Republic of Tanzania	955 (446 to 1841)	2389 (1050 to 5040)	17.92 (8.58 to 34.04)	17.32 (7.77 to 35.9)	-0.09 (-0.22 to 0.04)
Uganda	485 (233 to 889)	2621 (1207 to 5220)	14.26 (6.98 to 25.82)	27.21 (12.88 to 52.73)	1.99 (1.58 to 2.4)*
Zambia	390 (193 to 687)	1145 (381 to 3610)	23.97 (12.08 to 41.76)	25.37 (8.83 to 77.16)	0.17 (0.03 to 0.32)*
Botswana	16 (6 to 36)	33 (12 to 78)	6.6 (2.7 to 14.41)	4.99 (1.91 to 11.83)	-0.99 (-2.35 to 0.39)
Eswatini	11 (5 to 22)	24 (9 to 50)	7.34 (3.32 to 14.01)	9.15 (3.53 to 18.86)	0.69 (0.36 to 1.02)*
Lesotho	14 (6 to 32)	37 (16 to 75)	4.21 (1.72 to 9.66)	8.86 (3.94 to 17.72)	2.4 (1.83 to 2.98)*
Namibia	20 (10 to 38)	50 (21 to 100)	6.98 (3.46 to 12.88)	8.12 (3.55 to 16.2)	0.53 (0.09 to 0.96)*
South Africa	720 (547 to 1040)	942 (711 to 1443)	8.78 (6.68 to 12.44)	6.09 (4.6 to 9.27)	-1.49 (-2.7 to -0.26)*
Zimbabwe	318 (161 to 579)	1250 (541 to 2453)	16.64 (8.73 to 29.5)	34.41 (15.25 to 66.68)	2.39 (1.66 to 3.13)*
Benin	29 (13 to 60)	65 (28 to 137)	3.17 (1.46 to 6.37)	2.36 (1.02 to 4.96)	-0.96 (-1.15 to -0.78)*
Burkina Faso	77 (33 to 157)	146 (63 to 290)	4.25 (1.84 to 8.58)	3.11 (1.37 to 6.13)	-0.98 (-1.17 to -0.79)*

Cabo Verde	1 (0 to 1)	4 (1 to 8)	1.07 (0.49 to 2.12)	2.67 (0.63 to 5.51)	3.07 (2.66 to 3.48)*
Cameroon	98 (45 to 189)	242 (105 to 468)	4.9 (2.23 to 9.24)	3.56 (1.56 to 6.83)	-1.02 (-1.15 to -0.88)*
Chad	34 (15 to 70)	88 (42 to 165)	3.04 (1.37 to 6.14)	2.84 (1.36 to 5.31)	-0.14 (-0.34 to 0.07)
Coted'Ivoire	228 (112 to 423)	651 (303 to 1267)	9.41 (4.73 to 16.99)	10.39 (4.94 to 19.86)	0.38 (-0.1 to 0.85)
Gambia	8 (4 to 15)	27 (12 to 53)	4.1 (1.97 to 7.52)	4.94 (2.2 to 9.38)	0.71 (-0.56 to 1.98)
Ghana	9 (4 to 16)	27 (11 to 59)	0.29 (0.14 to 0.53)	0.32 (0.14 to 0.68)	0.36 (0.11 to 0.61)*
Guinea	75 (36 to 138)	182 (81 to 379)	5.87 (2.91 to 10.7)	5.94 (2.67 to 12.16)	0.03 (-0.16 to 0.22)
Guinea-Bissau	13 (6 to 25)	22 (10 to 41)	6.62 (3.07 to 12.51)	4.81 (2.24 to 8.89)	-1 (-1.17 to -0.83)*
Liberia	17 (7 to 35)	42 (19 to 81)	3.67 (1.55 to 7.27)	3.36 (1.49 to 6.39)	-0.26 (-0.53 to 0.01)
Mali	303 (160 to 537)	659 (284 to 1360)	17.34 (9.24 to 30.13)	13.74 (6.04 to 28.08)	-0.73 (-1.02 to -0.44)*
Mauritania	13 (6 to 26)	22 (10 to 45)	3.35 (1.52 to 6.4)	2.39 (1.08 to 4.91)	-1.08 (-1.31 to -0.84)*
Niger	49 (21 to 102)	91 (40 to 191)	3.33 (1.47 to 6.79)	2.17 (0.97 to 4.53)	-1.39 (-1.75 to -1.03)*
Nigeria	139 (81 to 239)	398 (221 to 739)	0.83 (0.49 to 1.44)	0.81 (0.45 to 1.48)	-0.12 (-0.29 to 0.05)

Sao Tome and Principe	0 (0 to 0)	0 (0 to 0)	0.32 (0.14 to 0.66)	0.36 (0.14 to 0.81)	0.45 (-0.17 to 1.08)
Senegal	54 (23 to 115)	91 (39 to 197)	3.71 (1.6 to 7.75)	2.68 (1.18 to 5.71)	-1.08 (-1.7 to -0.46)*
Sierra Leone	25 (10 to 52)	58 (25 to 117)	2.92 (1.17 to 5.94)	2.99 (1.29 to 6.03)	0.08 (-0.22 to 0.39)
Togo	30 (14 to 60)	62 (26 to 124)	4.08 (1.88 to 8.18)	3.07 (1.32 to 6.15)	-0.93 (-1.17 to -0.68)*

Table S4: Deaths of WCBA thyroid cancer in 1990 and 2021, and its ASR estimated AAPC from 1990 to 2021.

Location	Number, 95% UI		Age-standardized deaths rate (per 100 000), 95% UI		AAPC of incidence rate, No. (95% CI)
	1990	2021	1990	2021	
Global	2140 (1778 to 2648)	3260 (2578 to 4215)	0.18 (0.15 to 0.22)	0.16 (0.13 to 0.21)	-0.28 (-0.4 to -0.16)*
**SDI regions**					
Low SDI	310 (218 to 432)	655 (462 to 1030)	0.31 (0.22 to 0.44)	0.27 (0.19 to 0.42)	-0.57 (-0.66 to -0.48)*
Low-middle SDI	548 (418 to 761)	1140 (832 to 1599)	0.23 (0.17 to 0.31)	0.23 (0.17 to 0.33)	0.13 (-0.05 to 0.3)
Middle SDI	638 (520 to 792)	959 (751 to 1172)	0.17 (0.14 to 0.21)	0.14 (0.11 to 0.18)	-0.53 (-0.61 to -0.46)*
High-middle SDI	392 (321 to 457)	304 (252 to 381)	0.15 (0.13 to 0.18)	0.08 (0.07 to 0.1)	-2.01 (-2.27 to -1.74)*

High SDI	250 (235 to 267)	200 (180 to 229)	0.11 (0.1 to 0.11)	0.07 (0.06 to 0.08)	-1.37 (-1.54 to -1.21)*
**GBD super regions**					
Central Europe, Eastern Europe, and Central Asia	152 (144 to 160)	105 (95 to 116)	0.15 (0.14 to 0.16)	0.09 (0.08 to 0.1)	-1.92 (-2.74 to -1.1)*
High-income	237 (223 to 252)	166 (158 to 176)	0.1 (0.09 to 0.1)	0.06 (0.06 to 0.06)	-1.64 (-1.77 to -1.51)*
Latin America and Caribbean	135 (125 to 147)	230 (203 to 262)	0.16 (0.15 to 0.18)	0.14 (0.12 to 0.16)	-0.54 (-0.74 to -0.35)*
North Africa and Middle East	90 (64 to 139)	98 (75 to 132)	0.14 (0.1 to 0.22)	0.12 (0.09 to 0.16)	-0.38 (-0.57 to -0.18)*
South Asia	612 (455 to 872)	1380 (1003 to 1941)	0.26 (0.19 to 0.37)	0.29 (0.21 to 0.4)	0.33 (-0.03 to 0.7)
Southeast Asia, East Asia, and Oceania	675 (483 to 829)	701 (534 to 896)	0.17 (0.13 to 0.21)	0.12 (0.09 to 0.15)	-1.26 (-1.39 to -1.14)*
Sub-Saharan Africa	241 (172 to 332)	482 (330 to 801)	0.25 (0.18 to 0.35)	0.19 (0.13 to 0.32)	-0.88 (-0.95 to -0.82)*
**GBD regions**					
Central Asia	25 (22 to 28)	27 (23 to 32)	0.19 (0.17 to 0.21)	0.11 (0.09 to 0.13)	-1.82 (-3.12 to -0.5)*
Central Europe	63 (59 to 69)	26 (23 to 29)	0.2 (0.18 to 0.21)	0.08 (0.07 to 0.09)	-3.05 (-3.92 to -2.17)*
Eastern Europe	63 (59 to 69)	51 (44 to 61)	0.11 (0.11 to 0.12)	0.08 (0.07 to 0.1)	-1.41 (-2.42 to -0.4)*
Australasia	4 (3 to 5)	4 (3 to 5)	0.07 (0.06 to 0.09)	0.04 (0.03 to 0.06)	-1.43 (-2.38 to -0.47)*

High-income Asia Pacific	41 (34 to 52)	28 (23 to 36)	0.08 (0.07 to 0.11)	0.06 (0.05 to 0.07)	-1.19 (-1.52 to -0.85)*
High-income North America	50 (48 to 53)	58 (55 to 61)	0.07 (0.06 to 0.07)	0.06 (0.06 to 0.07)	-0.02 (-0.17 to 0.13)
Southern Latin America	21 (17 to 27)	19 (15 to 24)	0.18 (0.14 to 0.22)	0.1 (0.08 to 0.13)	-1.73 (-2.63 to -0.82)*
Western Europe	121 (111 to 131)	57 (53 to 62)	0.12 (0.11 to 0.13)	0.05 (0.05 to 0.05)	-2.84 (-2.95 to -2.74)*
Andean Latin America	19 (14 to 27)	36 (25 to 51)	0.25 (0.18 to 0.34)	0.21 (0.15 to 0.3)	-0.48 (-0.79 to -0.17)*
Caribbean	14 (11 to 18)	20 (15 to 28)	0.17 (0.13 to 0.22)	0.17 (0.12 to 0.23)	-0.07 (-0.58 to 0.43)
Central Latin America	60 (55 to 66)	110 (94 to 128)	0.18 (0.17 to 0.2)	0.16 (0.13 to 0.18)	-0.55 (-0.81 to -0.3)*
Tropical Latin America	41 (38 to 46)	63 (57 to 70)	0.12 (0.11 to 0.14)	0.1 (0.09 to 0.11)	-0.91 (-1.12 to -0.69)*
East Asia	418 (296 to 547)	272 (195 to 412)	0.15 (0.1 to 0.19)	0.07 (0.05 to 0.1)	-2.42 (-2.7 to -2.13)*
Oceania	1 (1 to 2)	3 (1 to 5)	0.1 (0.06 to 0.16)	0.08 (0.04 to 0.16)	-0.58 (-0.62 to -0.53)*
Southeast Asia	255 (174 to 320)	426 (299 to 551)	0.26 (0.18 to 0.32)	0.22 (0.15 to 0.29)	-0.45 (-0.54 to -0.36)*
Central Sub-Saharan Africa	9 (5 to 16)	20 (9 to 40)	0.09 (0.05 to 0.16)	0.08 (0.04 to 0.15)	-0.61 (-0.73 to -0.5)*
Eastern Sub-Saharan Africa	192 (129 to 277)	373 (244 to 664)	0.52 (0.35 to 0.75)	0.39 (0.26 to 0.69)	-0.94 (-1.03 to -0.85)*

Southern Sub-Saharan Africa	19 (14 to 25)	41 (27 to 62)	0.18 (0.13 to 0.23)	0.2 (0.13 to 0.3)	0.26 (-0.82 to 1.35)
Western Sub-Saharan Africa	21 (14 to 29)	48 (32 to 75)	0.06 (0.04 to 0.08)	0.05 (0.03 to 0.07)	-0.7 (-0.91 to -0.49)*
**Countries and territories**					
Armenia	1 (1 to 1)	1 (1 to 1)	0.14 (0.1 to 0.19)	0.13 (0.09 to 0.18)	-0.31 (-1 to 0.39)
Azerbaijan	2 (1 to 3)	2 (1 to 4)	0.12 (0.07 to 0.19)	0.07 (0.04 to 0.12)	-1.56 (-2.02 to -1.1)*
Georgia	2 (1 to 3)	2 (2 to 3)	0.15 (0.11 to 0.21)	0.24 (0.17 to 0.33)	1.31 (-0.43 to 3.08)
Kazakhstan	13 (12 to 15)	10 (8 to 12)	0.38 (0.33 to 0.43)	0.19 (0.15 to 0.23)	-1.82 (-4.61 to 1.05)
Kyrgyzstan	3 (2 to 5)	3 (2 to 5)	0.38 (0.26 to 0.52)	0.2 (0.13 to 0.28)	-2.4 (-4.12 to -0.66)*
Mongolia	1 (0 to 1)	2 (1 to 3)	0.24 (0.13 to 0.4)	0.17 (0.1 to 0.27)	-1.39 (-2.26 to -0.5)*
Tajikistan	0 (0 to 0)	0 (0 to 0)	0 (0 to 0)	0 (0 to 0)	-2.06 (-2.38 to -1.73)*
Turkmenistan	1 (1 to 1)	2 (1 to 3)	0.2 (0.17 to 0.22)	0.15 (0.11 to 0.21)	-0.72 (-3.35 to 1.98)
Uzbekistan	1 (1 to 2)	6 (4 to 8)	0.04 (0.03 to 0.05)	0.06 (0.04 to 0.09)	1.65 (0.92 to 2.37)*
Albania	1 (0 to 1)	0 (0 to 1)	0.12 (0.07 to 0.19)	0.06 (0.03 to 0.11)	-2.13 (-2.69 to -1.57)*

Bosnia and Herzegovina	1 (1 to 2)	1 (0 to 1)	0.11 (0.08 to 0.15)	0.06 (0.04 to 0.09)	-2.27<br>(-2.9 to -1.65)*
Bulgaria	4 (3 to 5)	2 (1 to 3)	0.17 (0.12 to 0.22)	0.11 (0.07 to 0.15)	-1.2<br>(-2.98 to 0.6)
Croatia	2 (1 to 3)	1 (0 to 1)	0.15 (0.11 to 0.2)	0.06 (0.04 to 0.08)	-3.4<br>(-4.76 to -2.02)*
Czechia	5 (4 to 6)	2 (1 to 3)	0.17 (0.13 to 0.22)	0.06 (0.04 to 0.08)	-3.24<br>(-4.12 to -2.35)*
Hungary	7 (5 to 9)	2 (2 to 3)	0.24 (0.18 to 0.32)	0.08 (0.06 to 0.11)	-3.4<br>(-4.45 to -2.33)*
Montenegro	0 (0 to 0)	0 (0 to 0)	0.14 (0.1 to 0.2)	0.09 (0.07 to 0.13)	-1.26<br>(-1.92 to -0.59)*
North Macedonia	1 (1 to 1)	0 (0 to 1)	0.14 (0.1 to 0.19)	0.07 (0.04 to 0.1)	-2.38<br>(-3.09 to -1.65)*
Poland	25 (24 to 27)	10 (8 to 11)	0.26 (0.25 to 0.28)	0.08 (0.07 to 0.1)	-3.34<br>(-4.86 to -1.79)*
Romania	10 (7 to 13)	4 (3 to 5)	0.17 (0.12 to 0.22)	0.07 (0.05 to 0.1)	-2.84<br>(-4.27 to -1.39)*
Serbia	3 (2 to 6)	2 (1 to 3)	0.13 (0.07 to 0.23)	0.07 (0.04 to 0.13)	-1.64<br>(-2.07 to -1.21)*
Slovakia	3 (2 to 4)	1 (1 to 3)	0.19 (0.12 to 0.31)	0.09 (0.05 to 0.16)	-2.1<br>(-2.94 to -1.25)*
Slovenia	1 (1 to 1)	0 (0 to 0)	0.16 (0.12 to 0.21)	0.04 (0.03 to 0.06)	-4.48<br>(-6.13 to -2.81)*
Belarus	4 (3 to 6)	2 (1 to 3)	0.18 (0.12 to 0.24)	0.08 (0.05 to 0.12)	-2.98<br>(-7.5 to 1.77)

Estonia	1 (1 to 1)	0 (0 to 0)	0.2 (0.14 to 0.27)	0.06 (0.04 to 0.09)	-4.08 (-5.25 to -2.9)*
Latvia	1 (1 to 2)	0 (0 to 1)	0.19 (0.13 to 0.26)	0.09 (0.07 to 0.13)	-2.32 (-4.02 to -0.58)*
Lithuania	2 (1 to 2)	1 (0 to 1)	0.18 (0.13 to 0.24)	0.07 (0.05 to 0.1)	-3.13 (-4.33 to -1.91)*
Republic of Moldova	1 (1 to 1)	1 (0 to 1)	0.09 (0.08 to 0.1)	0.05 (0.05 to 0.07)	-1.81 (-2.51 to -1.11)*
Russian Federation	42 (40 to 44)	35 (31 to 40)	0.12 (0.11 to 0.12)	0.08 (0.07 to 0.09)	-1.99 (-3.63 to -0.33)*
Ukraine	12 (9 to 17)	12 (6 to 19)	0.09 (0.06 to 0.13)	0.09 (0.05 to 0.15)	-0.06 (-1.08 to 0.97)
Australia	3 (2 to 4)	3 (2 to 4)	0.07 (0.05 to 0.09)	0.05 (0.03 to 0.06)	-1.26 (-2.57 to 0.06)
New Zealand	1 (0 to 1)	0 (0 to 1)	0.07 (0.05 to 0.09)	0.03 (0.02 to 0.04)	-2.46 (-4.45 to -0.42)*
Brunei Darussalam	0 (0 to 0)	0 (0 to 0)	0.21 (0.11 to 0.38)	0.15 (0.09 to 0.24)	-1.15 (-1.53 to -0.77)*
Japan	25 (24 to 26)	16 (16 to 17)	0.07 (0.06 to 0.07)	0.05 (0.05 to 0.05)	-0.98 (-1.64 to -0.32)*
Singapore	1 (1 to 1)	1 (0 to 1)	0.1 (0.07 to 0.13)	0.03 (0.02 to 0.05)	-3.25 (-3.75 to -2.75)*
Republic of Korea	14 (8 to 26)	11 (6 to 18)	0.13 (0.07 to 0.23)	0.07 (0.04 to 0.12)	-1.71 (-1.94 to -1.48)*
Canada	8 (6 to 10)	5 (3 to 6)	0.1 (0.07 to 0.13)	0.05 (0.04 to 0.07)	-2.18 (-3.09 to -1.26)*

Greenland	0 (0 to 0)	0 (0 to 0)	0.23 (0.11 to 0.38)	0.09 (0.04 to 0.18)	-2.98 (-3.52 to -2.45)*
United States of America	43 (41 to 44)	53 (51 to 56)	0.06 (0.06 to 0.06)	0.06 (0.06 to 0.07)	0.05 (-0.95 to 1.05)
Argentina	14 (10 to 19)	13 (9 to 18)	0.18 (0.13 to 0.24)	0.1 (0.07 to 0.14)	-1.66 (-2.69 to -0.61)*
Chile	6 (4 to 8)	5 (3 to 7)	0.19 (0.13 to 0.25)	0.09 (0.07 to 0.13)	-2.12 (-2.84 to -1.4)*
Uruguay	1 (1 to 2)	1 (1 to 2)	0.18 (0.13 to 0.25)	0.13 (0.09 to 0.18)	-0.78 (-1.16 to -0.4)*
Andorra	0 (0 to 0)	0 (0 to 0)	0.1 (0.05 to 0.17)	0.05 (0.03 to 0.09)	-2.12 (-2.24 to -1.99)*
Austria	3 (2 to 4)	1 (1 to 2)	0.16 (0.12 to 0.21)	0.05 (0.04 to 0.07)	-3.53 (-4.17 to -2.89)*
Belgium	3 (2 to 4)	2 (1 to 2)	0.12 (0.09 to 0.17)	0.06 (0.04 to 0.08)	-2.82 (-3.91 to -1.71)*
Cyprus	0 (0 to 1)	0 (0 to 0)	0.16 (0.08 to 0.27)	0.04 (0.02 to 0.08)	-4.29 (-4.9 to -3.69)*
Denmark	1 (1 to 2)	0 (0 to 1)	0.1 (0.08 to 0.13)	0.03 (0.02 to 0.04)	-4.21 (-5.39 to -3.01)*
Finland	2 (2 to 3)	1 (0 to 1)	0.16 (0.12 to 0.21)	0.05 (0.03 to 0.06)	-3.68 (-5.19 to -2.15)*
France	18 (14 to 24)	8 (6 to 11)	0.13 (0.09 to 0.16)	0.05 (0.03 to 0.07)	-2.89 (-3.08 to -2.71)*
Germany	28 (22 to 36)	10 (7 to 14)	0.13 (0.1 to 0.17)	0.05 (0.04 to 0.07)	-3.36 (-3.87 to -2.85)*

Greece	2 (2 to 2)	1 (1 to 2)	0.07 (0.06 to 0.08)	0.04 (0.04 to 0.05)	-1.62 (-2.46 to -0.77)*
Iceland	0 (0 to 0)	0 (0 to 0)	0.3 (0.22 to 0.4)	0.08 (0.06 to 0.11)	-4.08 (-5.49 to -2.65)*
Ireland	1 (1 to 1)	1 (0 to 1)	0.1 (0.07 to 0.13)	0.04 (0.03 to 0.06)	-2.66 (-3.4 to -1.92)*
Israel	2 (1 to 2)	1 (1 to 2)	0.14 (0.1 to 0.19)	0.05 (0.04 to 0.07)	-2.58 (-5.12 to 0.02)
Italy	23 (21 to 25)	9 (9 to 11)	0.15 (0.14 to 0.16)	0.06 (0.05 to 0.06)	-2.97 (-3.53 to -2.41)*
Luxembourg	0 (0 to 0)	0 (0 to 0)	0.14 (0.12 to 0.16)	0.04 (0.04 to 0.05)	-4 (-4.53 to -3.47)*
Malta	0 (0 to 0)	0 (0 to 0)	0.13 (0.09 to 0.17)	0.07 (0.05 to 0.1)	-2.04 (-3.28 to -0.79)*
Monaco	0 (0 to 0)	0 (0 to 0)	0.1 (0.05 to 0.17)	0.08 (0.04 to 0.14)	-0.65 (-0.78 to -0.52)*
Netherlands	5 (3 to 6)	3 (2 to 4)	0.11 (0.08 to 0.15)	0.07 (0.05 to 0.09)	-1.83 (-2.27 to -1.4)*
Norway	1 (1 to 1)	0 (0 to 0)	0.1 (0.09 to 0.11)	0.03 (0.02 to 0.03)	-4.16 (-5.83 to -2.47)*
Portugal	3 (2 to 5)	2 (1 to 2)	0.13 (0.1 to 0.17)	0.05 (0.04 to 0.07)	-2.99 (-4.05 to -1.92)*
San Marino	0 (0 to 0)	0 (0 to 0)	0.12 (0.06 to 0.19)	0.06 (0.03 to 0.11)	-2.18 (-2.55 to -1.8)*
Spain	11 (8 to 14)	5 (3 to 6)	0.11 (0.08 to 0.15)	0.03 (0.02 to 0.05)	-3.9 (-4.18 to -3.62)*

Sweden	2 (1 to 2)	1 (1 to 1)	0.07 (0.05 to 0.09)	0.03 (0.02 to 0.04)	-2.86 (-3.9 to -1.8)*
Switzerland	3 (2 to 4)	1 (0 to 1)	0.15 (0.11 to 0.19)	0.03 (0.02 to 0.04)	-5.27 (-6.7 to -3.83)*
United Kingdom	12 (11 to 13)	11 (10 to 11)	0.08 (0.08 to 0.08)	0.06 (0.06 to 0.06)	-0.78 (-1.09 to -0.47)*
Bolivia (Plurinational State of)	6 (3 to 10)	9 (5 to 17)	0.45 (0.21 to 0.78)	0.32 (0.16 to 0.56)	-1.08 (-1.24 to -0.93)*
Ecuador	4 (3 to 5)	11 (7 to 16)	0.19 (0.14 to 0.26)	0.23 (0.15 to 0.35)	0.98 (0.53 to 1.44)*
Peru	10 (6 to 15)	16 (9 to 28)	0.22 (0.13 to 0.34)	0.17 (0.09 to 0.29)	-0.54 (-2.07 to 1.01)
Antigua and Barbuda	0 (0 to 0)	0 (0 to 0)	0.18 (0.16 to 0.22)	0.16 (0.14 to 0.19)	-0.29 (-0.9 to 0.33)
Bahamas	0 (0 to 0)	0 (0 to 0)	0.22 (0.19 to 0.26)	0.23 (0.17 to 0.31)	-0.11 (-0.84 to 0.61)
Barbados	0 (0 to 0)	0 (0 to 0)	0.16 (0.14 to 0.19)	0.14 (0.11 to 0.19)	-0.57 (-1.22 to 0.09)
Belize	0 (0 to 0)	0 (0 to 0)	0.08 (0.07 to 0.1)	0.11 (0.09 to 0.13)	0.42 (-0.5 to 1.34)
Bermuda	0 (0 to 0)	0 (0 to 0)	0.15 (0.1 to 0.2)	0.1 (0.06 to 0.14)	-1.53 (-2.29 to -0.76)*
Cuba	4 (3 to 5)	4 (2 to 5)	0.14 (0.1 to 0.18)	0.12 (0.08 to 0.16)	-0.55 (-1.61 to 0.52)
Dominica	0 (0 to 0)	0 (0 to 0)	0.11 (0.07 to 0.17)	0.1 (0.05 to 0.17)	-0.26 (-0.42 to -0.09)*

Dominican Republic	2 (1 to 4)	4 (2 to 7)	0.15 (0.09 to 0.24)	0.14 (0.07 to 0.25)	-0.09 (-0.9 to 0.72)
Grenada	0 (0 to 0)	0 (0 to 0)	0.31 (0.21 to 0.43)	0.22 (0.15 to 0.32)	-1.22 (-2.18 to -0.26)*
Guyana	0 (0 to 0)	0 (0 to 1)	0.14 (0.1 to 0.2)	0.18 (0.11 to 0.29)	0.74 (-0.86 to 2.36)
Haiti	4 (2 to 8)	8 (4 to 15)	0.33 (0.15 to 0.6)	0.24 (0.11 to 0.45)	-0.91 (-1.18 to -0.63)*
Jamaica	1 (0 to 1)	1 (1 to 2)	0.12 (0.09 to 0.17)	0.18 (0.11 to 0.28)	1.34 (0.47 to 2.22)*
Puerto Rico	1 (1 to 2)	1 (0 to 1)	0.12 (0.09 to 0.16)	0.07 (0.05 to 0.11)	-1.76 (-2.99 to -0.51)*
Saint Kitts and Nevis	0 (0 to 0)	0 (0 to 0)	0.18 (0.15 to 0.21)	0.08 (0.06 to 0.11)	-2.61 (-3.6 to -1.62)*
Saint Lucia	0 (0 to 0)	0 (0 to 0)	0.33 (0.28 to 0.38)	0.26 (0.2 to 0.34)	-0.64 (-1.68 to 0.41)
Saint Vincent and the Grenadines	0 (0 to 0)	0 (0 to 0)	0.31 (0.27 to 0.36)	0.38 (0.3 to 0.46)	0.4 (-0.36 to 1.17)
Suriname	0 (0 to 0)	0 (0 to 0)	0.18 (0.11 to 0.29)	0.16 (0.09 to 0.27)	-0.1 (-1.04 to 0.85)
Trinidad and Tobago	1 (0 to 1)	1 (1 to 1)	0.19 (0.16 to 0.22)	0.19 (0.14 to 0.26)	0.01 (-0.42 to 0.44)
United States Virgin Islands	0 (0 to 0)	0 (0 to 0)	0.09 (0.05 to 0.14)	0.05 (0.02 to 0.11)	-1.59 (-2.02 to -1.16)*
Colombia	17 (13 to 22)	23 (15 to 33)	0.24 (0.18 to 0.31)	0.17 (0.12 to 0.25)	-1.11 (-1.43 to -0.78)*

Costa Rica	1 (1 to 2)	2 (1 to 2)	0.22 (0.16 to 0.28)	0.13 (0.09 to 0.18)	-2.04 (-3.27 to -0.8)*
El Salvador	2 (2 to 3)	3 (2 to 5)	0.22 (0.16 to 0.29)	0.18 (0.12 to 0.26)	-0.45 (-1.03 to 0.14)
Guatemala	3 (3 to 4)	7 (6 to 9)	0.23 (0.21 to 0.27)	0.19 (0.15 to 0.23)	-0.63 (-1.56 to 0.31)
Honduras	1 (0 to 1)	2 (1 to 4)	0.09 (0.05 to 0.15)	0.08 (0.04 to 0.15)	-0.34 (-0.66 to -0.01)*
Mexico	28 (27 to 30)	59 (49 to 70)	0.17 (0.16 to 0.18)	0.16 (0.13 to 0.19)	-0.06 (-0.41 to 0.28)
Nicaragua	1 (0 to 1)	2 (1 to 3)	0.13 (0.08 to 0.19)	0.1 (0.06 to 0.17)	-0.83 (-1.25 to -0.41)*
Panama	2 (1 to 2)	1 (1 to 2)	0.29 (0.21 to 0.4)	0.13 (0.09 to 0.2)	-2.35 (-3.68 to -0.99)*
Venezuela (Bolivarian Republic of)	5 (4 to 5)	11 (8 to 15)	0.12 (0.11 to 0.14)	0.15 (0.1 to 0.2)	0.67 (-0.12 to 1.47)
Brazil	40 (36 to 45)	60 (54 to 68)	0.12 (0.11 to 0.14)	0.09 (0.08 to 0.11)	-0.92 (-1.14 to -0.7)*
Paraguay	1 (1 to 2)	2 (1 to 4)	0.18 (0.11 to 0.28)	0.14 (0.08 to 0.24)	-0.65 (-1.18 to -0.12)*
Afghanistan	5 (1 to 14)	21 (7 to 43)	0.25 (0.06 to 0.74)	0.36 (0.11 to 0.72)	1.22 (1.02 to 1.43)*
Algeria	8 (4 to 14)	18 (9 to 33)	0.17 (0.09 to 0.3)	0.15 (0.08 to 0.28)	-0.34 (-0.56 to -0.11)*
Bahrain	0 (0 to 0)	0 (0 to 1)	0.21 (0.11 to 0.34)	0.14 (0.07 to 0.24)	-1.16 (-2.48 to 0.18)

Egypt	11 (6 to 18)	19 (11 to 33)	0.1 (0.06 to 0.16)	0.08 (0.05 to 0.13)	-0.73 (-1.01 to -0.45)*
Iran (Islamic Republic of)	5 (3 to 7)	17 (7 to 24)	0.05 (0.03 to 0.07)	0.07 (0.03 to 0.09)	1.06 (0.68 to 1.44)*
Iraq	6 (3 to 11)	15 (8 to 26)	0.18 (0.09 to 0.33)	0.15 (0.08 to 0.27)	-0.57 (-0.81 to -0.33)*
Jordan	1 (1 to 2)	3 (2 to 6)	0.21 (0.11 to 0.36)	0.1 (0.05 to 0.19)	-2.17 (-2.83 to -1.5)*
Kuwait	1 (0 to 1)	1 (1 to 2)	0.17 (0.12 to 0.23)	0.08 (0.05 to 0.11)	-2.95 (-7.78 to 2.14)
Lebanon	1 (1 to 2)	1 (1 to 2)	0.19 (0.09 to 0.33)	0.09 (0.05 to 0.16)	-2.43 (-2.81 to -2.05)*
Libya	2 (1 to 4)	6 (3 to 11)	0.28 (0.15 to 0.51)	0.29 (0.14 to 0.52)	0.14 (-0.27 to 0.55)
Morocco	7 (3 to 12)	12 (6 to 23)	0.12 (0.07 to 0.22)	0.12 (0.06 to 0.23)	-0.26 (-0.48 to -0.03)*
Oman	0 (0 to 0)	1 (0 to 1)	0.09 (0.05 to 0.17)	0.06 (0.03 to 0.11)	-1.1 (-1.32 to -0.88)*
Palestine	1 (0 to 1)	1 (1 to 2)	0.17 (0.08 to 0.3)	0.1 (0.06 to 0.18)	-1.51 (-1.85 to -1.18)*
Qatar	0 (0 to 0)	0 (0 to 1)	0.22 (0.12 to 0.39)	0.07 (0.03 to 0.14)	-3.82 (-5.03 to -2.59)*
Saudi Arabia	6 (3 to 12)	29 (15 to 54)	0.23 (0.11 to 0.44)	0.27 (0.14 to 0.5)	0.44 (0.3 to 0.59)*
Sudan	5 (1 to 14)	12 (5 to 23)	0.13 (0.03 to 0.36)	0.13 (0.05 to 0.24)	-0.08 (-0.16 to 0)

Syrian Arab Republic	2 (1 to 5)	3 (1 to 6)	0.09 (0.03 to 0.23)	0.08 (0.03 to 0.16)	-0.13 (-0.91 to 0.66)
Tunisia	2 (1 to 4)	4 (2 to 7)	0.14 (0.07 to 0.24)	0.12 (0.06 to 0.21)	-0.56 (-0.82 to -0.29)*
Turkey	24 (12 to 43)	20 (10 to 34)	0.2 (0.1 to 0.35)	0.08 (0.04 to 0.14)	-2.83 (-3.18 to -2.47)*
United Arab Emirates	1 (0 to 1)	3 (2 to 5)	0.29 (0.12 to 0.52)	0.14 (0.07 to 0.26)	-2.15 (-2.69 to -1.6)*
Yemen	2 (1 to 4)	8 (4 to 14)	0.08 (0.03 to 0.2)	0.11 (0.05 to 0.2)	1.12 (0.65 to 1.59)*
Bangladesh	59 (28 to 105)	96 (40 to 234)	0.28 (0.14 to 0.49)	0.21 (0.09 to 0.51)	-0.77 (-1.4 to -0.13)*
Bhutan	0 (0 to 1)	0 (0 to 1)	0.26 (0.12 to 0.49)	0.22 (0.09 to 0.52)	-0.65 (-0.81 to -0.5)*
India	447 (324 to 662)	928 (676 to 1278)	0.24 (0.17 to 0.35)	0.25 (0.18 to 0.34)	0.17 (-0.29 to 0.64)
Nepal	11 (4 to 20)	22 (9 to 44)	0.26 (0.11 to 0.49)	0.25 (0.11 to 0.5)	-0.07 (-0.18 to 0.04)
Pakistan	95 (58 to 154)	334 (185 to 563)	0.44 (0.28 to 0.7)	0.57 (0.32 to 0.96)	0.88 (0.75 to 1.01)*
China	397 (276 to 523)	251 (175 to 389)	0.14 (0.1 to 0.19)	0.07 (0.05 to 0.1)	-2.51 (-2.82 to -2.2)*
Democratic People's Republic of Korea	9 (5 to 17)	11 (5 to 20)	0.18 (0.09 to 0.32)	0.15 (0.07 to 0.28)	-0.44 (-0.49 to -0.39)*
Taiwan (Province of China)	12 (9 to 15)	10 (7 to 13)	0.23 (0.17 to 0.3)	0.14 (0.1 to 0.19)	-1.59 (-2.03 to -1.15)*

American Samoa	0 (0 to 0)	0 (0 to 0)	0.15 (0.08 to 0.25)	0.18 (0.1 to 0.31)	0.69 (0.12 to 1.27)*
Cook Islands	0 (0 to 0)	0 (0 to 0)	0.02 (0.01 to 0.03)	0.01 (0.01 to 0.03)	-0.25 (-1.15 to 0.67)
Micronesia (Federated States of)	0 (0 to 0)	0 (0 to 0)	0.1 (0.05 to 0.19)	0.1 (0.05 to 0.18)	-0.19 (-0.25 to -0.14)*
Fiji	0 (0 to 1)	1 (0 to 1)	0.25 (0.14 to 0.44)	0.22 (0.11 to 0.41)	-0.47 (-0.68 to -0.27)*
Guam	0 (0 to 0)	0 (0 to 0)	0.02 (0.01 to 0.05)	0.05 (0.03 to 0.06)	2.67 (0.9 to 4.48)*
Kiribati	0 (0 to 0)	0 (0 to 0)	0 (0 to 0.01)	0 (0 to 0.01)	-0.53 (-0.61 to -0.44)*
Marshall Islands	0 (0 to 0)	0 (0 to 0)	0.09 (0.05 to 0.15)	0.11 (0.05 to 0.21)	0.79 (0.73 to 0.84)*
Nauru	0 (0 to 0)	0 (0 to 0)	0.13 (0.05 to 0.24)	0.14 (0.05 to 0.27)	0.32 (0.23 to 0.41)*
Niue	0 (0 to 0)	0 (0 to 0)	0.07 (0.03 to 0.12)	0.09 (0.04 to 0.16)	0.9 (0.69 to 1.11)*
Northern Mariana Islands	0 (0 to 0)	0 (0 to 0)	0.05 (0.02 to 0.08)	0.04 (0.02 to 0.07)	-0.23 (-0.6 to 0.15)
Palau	0 (0 to 0)	0 (0 to 0)	0.08 (0.04 to 0.14)	0.07 (0.04 to 0.13)	-0.33 (-0.58 to -0.09)*
Papua New Guinea	1 (0 to 1)	2 (1 to 4)	0.07 (0.03 to 0.15)	0.07 (0.03 to 0.16)	-0.12 (-0.31 to 0.06)
Samoa	0 (0 to 0)	0 (0 to 0)	0.24 (0.12 to 0.43)	0.28 (0.13 to 0.53)	0.48 (0.42 to 0.53)*

Solomon Islands	0 (0 to 0)	0 (0 to 0)	0.07 (0.02 to 0.14)	0.09 (0.05 to 0.18)	1.01 (0.75 to 1.27)*
Tokelau	0 (0 to 0)	0 (0 to 0)	0.09 (0.04 to 0.16)	0.1 (0.05 to 0.18)	0.5 (0.16 to 0.85)*
Tonga	0 (0 to 0)	0 (0 to 0)	0.05 (0.03 to 0.09)	0.05 (0.02 to 0.09)	-0.13 (-0.24 to -0.02)*
Tuvalu	0 (0 to 0)	0 (0 to 0)	0.1 (0.05 to 0.18)	0.09 (0.05 to 0.16)	-0.34 (-0.44 to -0.25)*
Vanuatu	0 (0 to 0)	0 (0 to 0)	0.07 (0.03 to 0.13)	0.08 (0.04 to 0.14)	0.63 (0.29 to 0.97)*
Cambodia	6 (3 to 11)	12 (6 to 22)	0.3 (0.14 to 0.53)	0.29 (0.15 to 0.53)	-0.05 (-0.23 to 0.14)
Indonesia	94 (55 to 128)	150 (89 to 225)	0.24 (0.14 to 0.32)	0.19 (0.11 to 0.28)	-0.77 (-0.84 to -0.7)*
Lao People's Democratic Republic	3 (1 to 5)	5 (2 to 9)	0.35 (0.14 to 0.67)	0.26 (0.12 to 0.48)	-1.01 (-1.14 to -0.88)*
Malaysia	9 (5 to 16)	16 (9 to 28)	0.24 (0.13 to 0.41)	0.19 (0.11 to 0.34)	-0.59 (-0.79 to -0.39)*
Maldives	0 (0 to 0)	0 (0 to 0)	0.14 (0.05 to 0.28)	0.04 (0.02 to 0.08)	-4 (-4.13 to -3.88)*
Mauritius	0 (0 to 0)	0 (0 to 0)	0.12 (0.1 to 0.14)	0.07 (0.06 to 0.08)	-2.51 (-6.96 to 2.15)
Myanmar	30 (14 to 58)	33 (17 to 58)	0.36 (0.17 to 0.68)	0.22 (0.11 to 0.38)	-1.68 (-1.78 to -1.58)*
Philippines	42 (30 to 55)	87 (63 to 120)	0.33 (0.24 to 0.44)	0.32 (0.23 to 0.44)	-0.11 (-0.35 to 0.14)

Seychelles	0 (0 to 0)	0 (0 to 0)	0.09 (0.05 to 0.14)	0.06 (0.03 to 0.09)	-1.01 (-1.2 to -0.83)*
Sri Lanka	8 (4 to 13)	7 (3 to 13)	0.19 (0.1 to 0.31)	0.11 (0.05 to 0.21)	-1.67 (-2.37 to -0.96)*
Thailand	27 (15 to 44)	28 (15 to 49)	0.19 (0.11 to 0.32)	0.14 (0.07 to 0.24)	-1.05 (-1.55 to -0.54)*
Timor-Leste	0 (0 to 1)	0 (0 to 1)	0.18 (0.09 to 0.34)	0.17 (0.09 to 0.31)	-0.19 (-0.69 to 0.31)
Viet Nam	36 (17 to 67)	88 (43 to 161)	0.26 (0.13 to 0.47)	0.31 (0.15 to 0.58)	0.65 (0.54 to 0.76)*
Angola	2 (1 to 3)	5 (2 to 11)	0.09 (0.04 to 0.18)	0.08 (0.03 to 0.17)	-0.58 (-1.15 to -0.02)*
Central African Republic	1 (0 to 1)	1 (0 to 2)	0.12 (0.06 to 0.22)	0.09 (0.04 to 0.19)	-0.75 (-0.98 to -0.53)*
Congo	1 (0 to 1)	2 (1 to 3)	0.13 (0.06 to 0.23)	0.11 (0.05 to 0.23)	-0.29 (-0.9 to 0.33)
Democratic Republic of the Congo	6 (2 to 11)	12 (4 to 26)	0.08 (0.04 to 0.16)	0.07 (0.03 to 0.15)	-0.63 (-0.82 to -0.44)*
Equatorial Guinea	0 (0 to 0)	0 (0 to 1)	0.1 (0.05 to 0.19)	0.08 (0.03 to 0.17)	-0.71 (-1.53 to 0.11)
Gabon	0 (0 to 0)	0 (0 to 1)	0.12 (0.06 to 0.22)	0.09 (0.04 to 0.18)	-0.87 (-1.14 to -0.6)*
Burundi	5 (2 to 9)	8 (4 to 18)	0.45 (0.21 to 0.85)	0.31 (0.14 to 0.66)	-1.28 (-1.58 to -0.98)*
Comoros	0 (0 to 1)	1 (0 to 1)	0.35 (0.16 to 0.72)	0.34 (0.14 to 0.75)	-0.31 (-2.05 to 1.46)

Djibouti	0 (0 to 0)	1 (0 to 2)	0.24 (0.1 to 0.5)	0.25 (0.09 to 0.6)	0.1 (-0.21 to 0.4)
Eritrea	2 (1 to 4)	6 (2 to 11)	0.36 (0.19 to 0.63)	0.37 (0.17 to 0.74)	0.03 (-0.2 to 0.25)
Ethiopia	111 (60 to 184)	155 (85 to 322)	1.13 (0.63 to 1.87)	0.63 (0.35 to 1.32)	-1.89 (-2.03 to -1.74)*
Kenya	3 (2 to 6)	12 (7 to 23)	0.08 (0.05 to 0.14)	0.1 (0.06 to 0.19)	0.84 (0.7 to 1)*
Madagascar	8 (4 to 14)	21 (10 to 40)	0.35 (0.18 to 0.6)	0.32 (0.15 to 0.6)	-0.22 (-0.31 to -0.12)*
Malawi	7 (4 to 13)	16 (7 to 35)	0.35 (0.18 to 0.63)	0.38 (0.17 to 0.78)	0.32 (0.17 to 0.47)*
Mozambique	9 (4 to 19)	23 (9 to 57)	0.3 (0.14 to 0.66)	0.35 (0.14 to 0.86)	0.45 (0.27 to 0.64)*
Rwanda	9 (4 to 15)	10 (5 to 22)	0.61 (0.3 to 1.09)	0.32 (0.14 to 0.67)	-2.11 (-2.26 to -1.97)*
Somalia	5 (2 to 9)	13 (5 to 26)	0.33 (0.14 to 0.62)	0.31 (0.13 to 0.62)	-0.12 (-0.27 to 0.02)
South Sudan	3 (1 to 6)	6 (3 to 13)	0.25 (0.11 to 0.5)	0.31 (0.14 to 0.64)	0.67 (0.38 to 0.97)*
United Republic of Tanzania	16 (8 to 30)	39 (18 to 81)	0.31 (0.15 to 0.59)	0.3 (0.14 to 0.6)	-0.18 (-0.32 to -0.04)*
Uganda	8 (4 to 15)	42 (19 to 81)	0.25 (0.12 to 0.45)	0.46 (0.22 to 0.86)	1.84 (1.43 to 2.25)*
Zambia	6 (3 to 11)	19 (6 to 57)	0.42 (0.21 to 0.73)	0.43 (0.16 to 1.28)	0.07 (-0.07 to 0.21)

Botswana	0 (0 to 1)	1 (0 to 1)	0.13 (0.05 to 0.27)	0.09 (0.04 to 0.21)	-1.14 (-2.52 to 0.25)
Eswatini	0 (0 to 0)	0 (0 to 1)	0.14 (0.06 to 0.26)	0.17 (0.07 to 0.35)	0.53 (0.07 to 1)*
Lesotho	0 (0 to 1)	1 (0 to 1)	0.08 (0.03 to 0.18)	0.17 (0.08 to 0.34)	2.39 (1.83 to 2.95)*
Namibia	0 (0 to 1)	1 (0 to 2)	0.13 (0.06 to 0.23)	0.14 (0.06 to 0.28)	0.42 (0.03 to 0.82)*
South Africa	13 (10 to 18)	17 (13 to 25)	0.16 (0.12 to 0.22)	0.11 (0.08 to 0.16)	-1.34 (-2.74 to 0.08)
Zimbabwe	5 (3 to 10)	22 (10 to 42)	0.3 (0.16 to 0.53)	0.62 (0.28 to 1.18)	2.27 (1.46 to 3.09)*
Benin	1 (0 to 1)	1 (0 to 2)	0.06 (0.03 to 0.12)	0.04 (0.02 to 0.09)	-1.08 (-1.26 to -0.89)*
Burkina Faso	1 (1 to 3)	3 (1 to 5)	0.08 (0.03 to 0.16)	0.06 (0.02 to 0.11)	-1.05 (-1.24 to -0.86)*
Cabo Verde	0 (0 to 0)	0 (0 to 0)	0.02 (0.01 to 0.04)	0.04 (0.01 to 0.09)	2.79 (2.4 to 3.19)*
Cameroon	2 (1 to 3)	4 (2 to 8)	0.09 (0.04 to 0.17)	0.06 (0.03 to 0.12)	-1.15 (-1.26 to -1.04)*
Chad	1 (0 to 1)	2 (1 to 3)	0.06 (0.03 to 0.11)	0.05 (0.03 to 0.1)	-0.19 (-0.39 to 0.01)
Coted'Ivoire	4 (2 to 7)	11 (5 to 20)	0.17 (0.08 to 0.29)	0.18 (0.09 to 0.33)	0.22 (-0.23 to 0.68)
Gambia	0 (0 to 0)	0 (0 to 1)	0.07 (0.04 to 0.13)	0.08 (0.04 to 0.16)	0.61 (-0.63 to 1.86)

Ghana	0 (0 to 0)	0 (0 to 1)	0.01 (0 to 0.01)	0.01 (0 to 0.01)	0.19 (-0.05 to 0.44)
Guinea	1 (1 to 2)	3 (1 to 6)	0.1 (0.05 to 0.19)	0.1 (0.05 to 0.21)	-0.08 (-0.27 to 0.11)
Guinea-Bissau	0 (0 to 0)	0 (0 to 1)	0.12 (0.06 to 0.23)	0.09 (0.04 to 0.16)	-1.06 (-1.21 to -0.9)*
Liberia	0 (0 to 1)	1 (0 to 1)	0.07 (0.03 to 0.13)	0.06 (0.03 to 0.11)	-0.41 (-0.68 to -0.14)*
Mali	5 (3 to 9)	11 (5 to 22)	0.31 (0.17 to 0.53)	0.24 (0.11 to 0.48)	-0.82 (-1.03 to -0.61)*
Mauritania	0 (0 to 0)	0 (0 to 1)	0.06 (0.03 to 0.12)	0.04 (0.02 to 0.08)	-1.25 (-1.48 to -1.03)*
Niger	1 (0 to 2)	2 (1 to 3)	0.06 (0.03 to 0.12)	0.04 (0.02 to 0.08)	-1.45 (-1.77 to -1.13)*
Nigeria	2 (1 to 4)	7 (4 to 12)	0.01 (0.01 to 0.03)	0.01 (0.01 to 0.03)	-0.25 (-0.42 to -0.08)*
Sao Tome and Principe	0 (0 to 0)	0 (0 to 0)	0.01 (0 to 0.01)	0.01 (0 to 0.01)	0.29 (-0.26 to 0.83)
Senegal	1 (0 to 2)	2 (1 to 3)	0.07 (0.03 to 0.14)	0.05 (0.02 to 0.1)	-1.16 (-1.77 to -0.55)*
Sierra Leone	0 (0 to 1)	1 (0 to 2)	0.05 (0.02 to 0.11)	0.05 (0.02 to 0.11)	-0.02 (-0.3 to 0.25)
Togo	1 (0 to 1)	1 (0 to 2)	0.07 (0.03 to 0.15)	0.05 (0.02 to 0.11)	-1.03 (-1.25 to -0.81)*

Table S5 Decomposition analysis of change in WCBA thyroid cancer incidence, prevalence, DALYs, and deaths by SDIs and regions, 1990 to 2021

	Percent change of Aging	Percent change of Population	Percent change of Epidemiological change	Overall percent change
**Incidence**				
Global	21.44	62.72	72.70	156.86
Low SDI	1.33	188.97	101.83	292.13
Low-middle SDI	15.56	130.88	140.27	286.70
Middle SDI	42.77	65.90	139.63	248.30
High-middle SDI	33.97	13.20	39.98	87.15
High SDI	13.37	9.02	38.47	60.87
Central Europe, Eastern Europe, and Central Asia	19.59	-4.95	5.25	19.90
Central Asia	32.06	48.61	-10.46	70.21
Central Europe	16.18	-16.13	-16.93	-16.88
Eastern Europe	22.12	-16.27	31.46	37.31
High-income	12.58	3.23	21.27	37.07
Australasia	13.67	44.03	54.57	112.26
High-income Asia Pacific	21.42	-23.31	51.49	49.61
High-income North America	6.97	16.30	48.17	71.44
Southern Latin America	13.68	46.70	23.05	83.43
Western Europe	13.68	-2.52	-10.23	0.93
Latin America and Caribbean	43.43	88.12	104.47	236.02
Andean Latin America	47.72	146.81	180.82	375.35
Caribbean	22.96	36.98	42.86	102.81
Central Latin America	49.77	99.94	118.37	268.08
Tropical Latin America	40.87	74.39	74.23	189.49

North Africa and Middle East	50.12	175.15	174.03	399.31
South Asia	12.40	154.07	180.50	346.97
Southeast Asia, East Asia, and Oceania	45.57	22.67	103.70	171.94
East Asia	46.73	-1.20	83.69	129.22
Oceania	19.69	142.18	30.59	192.45
Southeast Asia	41.34	84.81	126.17	252.32
Sub-Saharan Africa	5.55	178.25	60.03	243.84
Central Sub-Saharan Africa	7.67	194.78	61.38	263.83
Eastern Sub-Saharan Africa	3.78	180.53	72.51	256.82
Southern Sub-Saharan Africa	27.57	79.28	41.96	148.81
Western Sub-Saharan Africa	5.60	204.24	57.64	267.48
**Prevalence**				
Global	21.51	63.18	75.21	159.91
Low SDI	1.30	194.14	114.17	309.60
Low-middle SDI	15.68	133.31	148.52	297.52
Middle SDI	43.02	66.79	145.36	255.17
High-middle SDI	34.02	13.28	41.80	89.11
High SDI	13.35	9.04	39.10	61.49
Central Europe, Eastern Europe, and Central Asia	19.51	-4.96	5.85	20.40
Central Asia	31.95	48.77	-9.33	71.40
Central Europe	16.14	-16.19	-16.08	-16.14
Eastern Europe	22.00	-16.28	31.81	37.52
High-income	12.56	3.23	21.82	37.61
Australasia	13.66	44.13	55.28	113.07
High-income Asia Pacific	21.42	-23.37	52.17	50.23

High-income North America	6.92	16.32	48.43	71.67
Southern Latin America	13.71	47.03	25.06	85.79
Western Europe	13.68	-2.53	-9.67	1.47
Latin America and Caribbean	43.62	89.13	109.26	242.01
Andean Latin America	48.46	149.86	191.26	389.57
Caribbean	22.93	37.18	44.58	104.69
Central Latin America	49.97	101.09	123.46	274.52
Tropical Latin America	41.00	75.15	78.17	194.33
North Africa and Middle East	50.22	176.05	176.80	403.07
South Asia	12.49	157.40	191.09	360.98
Southeast Asia, East Asia, and Oceania	45.84	22.97	108.62	177.43
East Asia	47.13	-1.22	88.31	134.23
Oceania	19.67	143.19	33.40	196.26
Southeast Asia	41.47	85.91	131.83	259.21
Sub-Saharan Africa	5.50	182.51	70.15	258.15
Central Sub-Saharan Africa	7.72	199.01	70.90	277.63
Eastern Sub-Saharan Africa	3.73	186.01	85.57	275.32
Southern Sub-Saharan Africa	27.05	78.89	40.68	146.61
Western Sub-Saharan Africa	5.54	207.48	64.75	277.77
**DALYs**				
Global	11.90	48.39	1.44	61.72
Low SDI	0.66	137.77	-19.33	119.09
Low-middle SDI	8.28	92.09	13.96	114.33
Middle SDI	25.16	41.68	-5.08	61.77
High-middle SDI	22.33	9.13	-41.27	-9.82

High SDI	10.22	6.99	-18.50	-1.30
Central Europe, Eastern Europe, and Central Asia	16.08	-3.98	-36.57	-24.48
Central Asia	25.47	40.19	-54.20	11.46
Central Europe	13.49	-12.76	-54.84	-54.10
Eastern Europe	18.66	-13.03	-15.40	-9.77
High-income	9.97	2.56	-26.23	-13.70
Australasia	10.04	33.02	-19.75	23.31
High-income Asia Pacific	16.83	-17.87	-6.06	-7.10
High-income North America	6.28	14.12	12.41	32.81
Southern Latin America	9.88	34.63	-47.32	-2.81
Western Europe	10.66	-2.01	-50.16	-41.51
Latin America and Caribbean	29.17	61.20	-12.64	77.73
Andean Latin America	26.33	87.32	-15.06	98.59
Caribbean	17.20	31.21	-0.01	48.41
Central Latin America	33.48	68.25	-10.82	90.92
Tropical Latin America	28.91	53.55	-23.71	58.76
North Africa and Middle East	29.40	116.04	7.21	152.65
South Asia	5.97	102.98	23.15	132.11
Southeast Asia, East Asia, and Oceania	28.01	13.92	-27.13	14.79
East Asia	25.83	-0.68	-47.05	-21.89
Oceania	16.28	122.14	-21.18	117.23
Southeast Asia	28.35	56.31	-9.16	75.50
Sub-Saharan Africa	3.16	137.31	-34.00	106.47
Central Sub-Saharan Africa	5.46	154.98	-26.17	134.27
Eastern Sub-Saharan Africa	2.05	134.32	-35.13	101.24

Southern Sub-Saharan Africa	21.78	72.75	17.87	112.41
Western Sub-Saharan Africa	3.80	163.83	-28.18	139.45
**Deaths**				
Global	14.83	47.01	-9.51	52.33
Low SDI	1.17	135.41	-25.51	111.08
Low-middle SDI	11.17	90.68	6.15	108.00
Middle SDI	29.73	40.29	-19.71	50.32
High-middle SDI	24.84	8.65	-55.92	-22.44
High SDI	11.09	6.39	-37.49	-20.01
Central Europe, Eastern Europe, and Central Asia	18.16	-3.85	-45.42	-31.11
Central Asia	29.60	40.19	-59.85	9.94
Central Europe	15.12	-12.34	-62.16	-59.38
Eastern Europe	21.07	-12.43	-27.62	-18.98
High-income	10.85	2.36	-43.08	-29.87
Australasia	10.66	29.61	-45.03	-4.77
High-income Asia Pacific	17.51	-15.66	-32.38	-30.54
High-income North America	7.68	13.17	-4.96	15.89
Southern Latin America	10.89	33.44	-55.99	-11.67
Western Europe	11.48	-1.88	-62.27	-52.67
Latin America and Caribbean	33.17	60.10	-22.78	70.49
Andean Latin America	29.41	85.26	-26.43	88.23
Caribbean	20.47	31.14	-4.25	47.36
Central Latin America	37.98	67.01	-21.99	83.00
Tropical Latin America	32.73	52.57	-33.56	51.74
North Africa and Middle East	31.92	108.19	-21.12	118.99

South Asia	8.93	101.44	15.28	125.65
Southeast Asia, East Asia, and Oceania	32.04	13.38	-41.52	3.90
East Asia	28.69	-0.64	-63.10	-35.05
Oceania	18.57	120.64	-27.83	111.38
Southeast Asia	32.93	55.13	-20.78	67.28
Sub-Saharan Africa	4.64	135.49	-39.98	100.16
Central Sub-Saharan Africa	6.46	153.67	-30.24	129.89
Eastern Sub-Saharan Africa	3.20	132.17	-41.53	93.85
Southern Sub-Saharan Africa	26.08	73.26	15.88	115.22
Western Sub-Saharan Africa	5.09	161.64	-34.38	132.35
